# Mitochondrial Genome Evolution of Placozoans: Gene Rearrangements and Repeat Expansions

**DOI:** 10.1093/gbe/evaa213

**Published:** 2020-10-08

**Authors:** Hideyuki Miyazawa, Hans-Jürgen Osigus, Sarah Rolfes, Kai Kamm, Bernd Schierwater, Hiroaki Nakano

**Affiliations:** 1 Center for Genome Informatics, Joint Support-Center for Data Science Research, Research Organization of Information and Systems, Mishima, Shizuoka, Japan; 2 Shimoda Marine Research Center, University of Tsukuba, Shimoda, Shizuoka, Japan; 3 Division of Molecular Evolution, Institute of Animal Ecology, University of Veterinary Medicine Hannover, Foundation, Germany

**Keywords:** Placozoa, phylogeny, small inverted repeat, gene order, evolution, mitochondrial genome

## Abstract

Placozoans, nonbilaterian animals with the simplest known metazoan bauplan, are currently classified into 20 haplotypes belonging to three genera, *Polyplacotoma*, *Trichoplax*, and *Hoilungia*. The latter two comprise two and five clades, respectively. In *Trichoplax* and *Hoilungia*, previous studies on six haplotypes belonging to four different clades have shown that their mtDNAs are circular chromosomes of 32–43 kb in size, which encode 12 protein-coding genes, 24 tRNAs, and two rRNAs. These mitochondrial genomes (mitogenomes) also show unique features rarely seen in other metazoans, including open reading frames (ORFs) of unknown function, and group I and II introns. Here, we report seven new mitogenomes, covering the five previously described haplotypes H2, H17, H19, H9, and H11, as well as two new haplotypes, H23 (*clade III*) and H24 (*clade VII*). The overall gene content is shared between all placozoan mitochondrial genomes, but genome sizes, gene orders, and several exon–intron boundaries vary among clades. Phylogenomic analyses strongly support a tree topology different from previous 16S rRNA analyses, with *clade VI* as the sister group to all other *Hoilungia* clades. We found small inverted repeats in all 13 mitochondrial genomes of the *Trichoplax* and *Hoilungia* genera and evaluated their distribution patterns among haplotypes. Because *Polyplacotoma mediterranea* (H0), the sister to the remaining haplotypes, has a small mitochondrial genome with few small inverted repeats and ORFs, we hypothesized that the proliferation of inverted repeats and ORFs substantially contributed to the observed increase in the size and GC content of the *Trichoplax* and *Hoilungia* mitochondrial genomes.

Significance

Placozoans, one of the four nonbilaterian animal phyla, is currently classified into 20 mitochondrial 16S rRNA haplotypes. Although seven complete placozoan mitochondrial genomes (mitogenomes) have been characterized to date, the overall placozoan mitogenome evolution remains poorly understood. We doubled the number of characterized placozoan mitogenomes from 7 to 14 and found their typical mitochondrial gene content to be highly conserved within this phylum, whereas genome sizes, gene orders, and gene fragmentation patterns were highly diverse. We further resolved the higher-level phylogenetic relationships within placozoans and analyzed the proliferation of small inverted repeats in placozoan mtDNAs. In summary, our results provide new insights into the complex evolutionary pathways of placozoan mitogenomes.

Highlights

- The number of known mitochondrial genomes for Placozoa was doubled from 7 to 14.
- The phylogenomic analysis of mitochondrial genes revealed clade-level relationships in placozoans.
- The proliferation of small inverted repeat sequences is a dominant factor in the evolution of placozoan mitochondrial genomes.
- Comparative analyses revealed highly complex gene rearrangements and gene fragmentation patterns.

## Introduction

Mitochondria are double membrane-bound organelles that play a crucial role in generating ATP by oxidative phosphorylation. These organelles possess their own genome and are found in most eukaryotes (e.g., Gray 2012). Although the majority of the originally mitochondrial-encoded genes have been transferred to the nuclear genome, a small set of genes is still encoded by the mitochondrial genome, and their evolution is substantially affected by the nuclear-encoded genes (see e.g., Haig 2016). Early studies on mostly bilaterian mitochondrial genomes (mitogenomes) reasoned that the structure, size, and gene content of animal mtDNA are highly uniform (see e.g., Boore 1999). However, more recent studies on nonbilaterian metazoans (i.e., Cnidaria, Placozoa, Ctenophora, and Porifera) have revealed that their mitogenomes show considerably high variation (see Lavrov and Pett 2016 for overview). For example, tRNA genes are likely absent in mitogenomes from Ctenophora, and linearized mitogenomes have been reported from some lineages in Cnidaria and Porifera. The size of the mitogenome can be as large as >50 kb (e.g., *Clathrina clathrus*, Porifera, Calcinea) (Lavrov et al. 2013) or as small as 10 kb (e.g., *Mnemiopsis leidyi*, Ctenophora, Tentaculata) (Pett et al. 2011). In addition, the rates of sequence evolution can differ substantially and are high, for instance in Ctenophora, but are comparatively low in some lineages of cnidarians and poriferans (see e.g., Osigus et al. 2013).

Placozoans are small free-living marine nonbilaterian animals with a simple two-layer body plan and ameba-like body shape. They have been collected globally from tropical to temperate coastal waters, and mitochondrial 16S rRNA genotyping has revealed substantial genetic diversity within placozoans. In total, 20 placozoan 16S haplotypes (H0–H19) have been reported, with 16S-based phylogenetic analyses supporting the subdivision of H1–H19 into seven distinct 16S *clades I–VII* (see below) (Eitel et al. 2013; Nakano 2014; Miyazawa and Nakano 2018; Osigus et al. 2019). Ultrastructural morphological differences between some of these lineages have been found (Guidi et al. 2011), but *Trichoplax adhaerens* has been the only species described in the phylum for more than 130 years (Eitel et al. 2013). A recent comparative genomics study revealed large nuclear genomic differences between *T. adhaerens* (H1) and haplotype H13, leading to the description of a second placozoan species, *Hoilungia hongkongensis* (Eitel et al. 2018). Furthermore, the new and highly unusual haplotype H0, which forms a sister group to all other haplotypes, was recently described as a new species in a new genus, *Polyplacotoma mediterranea* (Osigus et al. 2019). The 19 haplotypes (excluding *P. mediterranea*) were provisionally divided into *Hoilungia* and *Trichoplax* groups (Eitel et al. 2018). Thus far, *Hoilungia* comprises 15 16S haplotypes and has been further subdivided into two groups: A1 (*clade III*) and A2 (containing *clades IV*–*VII*) (Eitel and Schierwater 2010). The *Trichoplax* group comprises four 16S haplotypes in two clades, namely the 16S *clades I* and *II* (Voigt et al. 2004; Eitel and Schierwater 2010; Eitel et al. 2013).

From the two placozoan genera *Trichoplax* and *Hoilungia*, mitogenomes have been sequenced from six haplotypes: H1 from *clade I*, H3 from *II*, H8 from *III*, and H4, H13, and H15 from *V* (Dellaporta et al. 2006; Signorovitch et al. 2007; Miyazawa et al. 2012; Eitel et al. 2018). All these mitogenomes are circular and not only possess 38 mitochondrial genes (i.e., *cox1*-*3*, *nad1*-*6*, *nad4L*, *cob*, *atp6*, two rRNAs, and 24 tRNAs) but also possess a number of features rarely seen in other animals: 1) open reading frames (ORFs) of unknown function (e.g., Dellaporta et al. 2006; Signorovitch et al. 2007; Miyazawa et al. 2012; Chi et al. 2018; Eitel et al. 2018); 2) *cis-* and *trans-*splicing group I and *trans-*splicing group II introns (Burger et al. 2009); 3) a single nucleotide exon in *cox1* (Osigus et al. 2017; Eitel et al. 2018); and 4) a low nucleotide substitution rate of protein-coding genes reported (e.g., from *clade V*) (Miyazawa et al. 2012). One large inversion of >8 kb in length, which includes several genes, has occurred between *Hoilungia* and *Trichoplax* (Signorovitch et al. 2007). Another inversion of a fragment containing multiple ORFs was found within *clade V* (Miyazawa et al. 2012). The size differences of the *Trichoplax* and *Hoilungia* mitogenomes (up to 11-kb size difference) are related to variations in intergenic region (IGR) lengths (Signorovitch et al. 2007), and several large (>80 bp) insertions and deletions (indels) have been observed even within clades (Miyazawa et al. 2012). The compact mitogenome of *P. mediterranea* is only 23 kb in size and thus by far the smallest among placozoans. It harbors considerably smaller IGRs than all other haplotypes (Osigus et al. 2019). Overall, the evolutionary processes that gave rise to the diversity of placozoan mitogenomes remain unclear.

Short palindromic repeats (inverted repeats or hairpins) are sequences with a total length of 10–100 bp possessing specific characteristics (Smith and Lee 2009). They consist of an initial sequence, a reverse complement sequence downstream, and a loop sequence located between these stem sequences. In this way, they form a secondary hairpin structure with a double-stranded stem. Such repeats have been identified, for instance, in the mitogenomes of yeast (Bullerwell et al. 2003), chytridiomycetes (Paquin et al. 2000), green algae (Smith and Lee 2009), sponges (Lavrov and Lang 2005), and their overall frequencies in mitogenomes vary among eukaryotes (Čechová et al. 2018). In sponge mitogenomes, differences in the number and locations of these repeats and their proliferation in both the IGRs and protein-coding genes have been reported even between closely related species (Lavrov 2010). Based on this observation, these repeats are unlikely to have adaptive relevance and might be mobile DNA (Erpenbeck et al. 2009; Lavrov et al. 2012; Imešek et al. 2013). In placozoan mitogenomes, hairpin-forming sequences have also been reported (Signorovitch et al. 2006, 2007; Eitel and Schierwater 2010) but have yet to be comprehensively analyzed and discussed in the context of placozoan mitogenome evolution.

In this study, we doubled the number of characterized placozoan mtDNAs by adding seven new mitogenomes (two of them from new haplotypes). The phylogenetic relationships within *Hoilungia* were resolved by analyzing different mitochondrial data sets. In addition, we identified an extensive proliferation of repetitive sequences, including large numbers of short palindromic repeats, in all 13 *Hoilungia* and *Trichoplax* mitogenomes. Such an extensive proliferation of small inverted repeats (SIRs) is a highly rare characteristic among metazoans, only sporadically found in a few animal groups, such as freshwater sponges (Lavrov et al. 2012). We show that the proliferation of these repeats can have a substantial impact on placozoan mitogenome characteristics, such as genome size, GC content, and mutations in coding genes. In summary, we provide improved scenarios for both the placozoan phylogeny and the complex evolution of placozoan mitogenomes.

## Materials and Methods

### Mitochondrial Genome Sequencing

For H2, H9, H11, H17, and H19, animal sampling, DNA extraction, and 16S haplotype determination were performed as previously described (Miyazawa and Nakano 2018). Several short regions (<1 kb) of each mitochondrial genome were first amplified by polymerase chain reaction (PCR) using universal primers (supplementary table S1, Supplementary Material online), and the PCR products were Sanger sequenced using FASMAC sequencing service (Atsugi, Japan). Long intermediate regions (>5,000 bp each) were amplified by PCR using specific primers with barcode sequences (supplementary table S1, Supplementary Material online) and sequenced by Macrogen Japan (Kyoto, Japan) using PacBio RS II Multiplexing Targeted Sequencing (Nakano et al. 2017). Short and long PCR amplifications were performed using Ex-Taq and LA-Taq (Takara Bio, Otsu, Japan). Short and long PCR products were purified using exonuclease I and alkaline phosphatase (calf intestine) (Takara Bio), and the QIAquick PCR purification kit (Qiagen, Hilden, Germany), respectively. Long and low-quality reads generated by PacBio RS II were classified according to their barcode sequences using standalone BLAST (BlastN version 2.2.29) and aligned with MAFFT FFT-NS-2 v7.221 with a gap opening penalty of 0.1 (Katoh and Standley 2013). The barcode sequences and ambiguous sites in the alignments were excluded using in-house Perl scripts (https://bitbucket.org/hmiyazawa1984/placozoan_mitogenome/src/master/: last accessed September 27, 2020). The mitochondrial genomes of these five placozoan haplotypes were reconstructed by concatenating the long PCR fragments.

In 2015, the clonal strain H23 “Oberjatzas - OJ Gamma” (*clade III*) was provided by Ulrike and Günter Oberjatzas (Hannover, Germany) from their private seawater aquarium, containing marine samples of unknown geographic origin. The total DNA sample of haplotype H24 “Aq2-1” (*clade VII*) was obtained from the DNA collection at the Institute of Animal Ecology (TiHo Hannover). The geographic origin of haplotype H24 “Aq2-1” is unknown because it originates from an aquarium, containing multiple seawater samples of unknown origin. Total DNA of H23 was extracted from clonal animal cultures using a standard phenol–chloroform protocol, as previously described by Ender and Schierwater (2003), and that of H24 was amplified before sequencing using the REPLI-g Mini Kit (Qiagen) following the manufacturer’s recommendations. The characterization of the diagnostic 16S rDNA fragment by PCR was conducted as previously described (Eitel and Schierwater 2010). As H20–H22 are already provisionally assigned to other placozoan haplotypes (Eitel M, personal communication), the two new haplotypes were named H23 and H24, respectively. The sequencing of total DNA from H23 and H24 was conducted on an Illumina HiSeq2500, as previously described (Osigus et al. 2019). The complete mitochondrial genomes of H23 and H24 were assembled using Geneious version 8.1. By using the “map to reference” function of the Geneious standard mapper, an iterative mapping approach with the diagnostic 16S rRNA gene fragment as the starting point was conducted under the “medium sensitivity” setting. The draft mitochondrial genomes were improved by the subsequent mapping of the paired-end read data sets to the respective mtDNA sequences using the Geneious standard mapper under the “highest sensitivity” setting.

### Annotation of the Mitochondrial Genomes

Mitochondrial protein-coding genes, rRNA genes, and ORFs were annotated with MFannot using the Mold, Protozoan, and Coelenterate Mitochondria genetic code (Beck and Lang 2010; http://megasun.bch.umontreal.ca/cgi-bin/mfannot/mfannotInterface.pl: last accessed Octorber 2, 2019) and improved with standalone BLAST similarity searches (BlastN and BlastX v2.2.29) using the previously published placozoan protein mitogenomes as a reference. The GenBank accession numbers of the placozoan mitogenomes are as follows: H0, MH682141.1; H1, NC_008151.2; H3, NC_008834.2; H4, NC_008833.2; H8, NC_008832.2; and H15, NC_015309.1. The nucleotide sequence and annotation data of H13 mitogenomes were downloaded from the genome repository (https://bitbucket.org/molpalmuc/hoilungia-genome/src/master/mitochondrial_genome/: last accessed August 2, 2018). *Cox1* annotation was conducted using KY310743.1 (Osigus et al. 2017) as a reference. For the identification of the *cox1* single base pair exon, a sequence consisting of a single base pair exon and the surrounding introns was used for similarity search. Annotations of tRNA and group I introns were performed using RNAweasel (http://megasun.bch.umontreal.ca/RNAweasel/: last accessed Octorber 2, 2019). The secondary structure of trnS (uga) of H0 was predicted using the tRNAscan-SE web server (Lowe and Chan 2016; http://lowelab.ucsc.edu/tRNAscan-SE/index.html: last accessed Febrary 5, 2019). The group I introns of previously reported placozoan mitogenomes (H0, H1, H3, H4, H8, H13, and H15) were reannotated using RNAweasel to prevent artifacts caused by different programs. Domain V of the group II introns was also annotated using RNAweasel. Homology searches with NCBI BlastX were performed on ORFs of unknown function identified in H2, H9, H11, H17, H19, H23, and H24. The GC content was calculated in a 100-bp window with a 10-bp step size using in-house Perl scripts (https://bitbucket.org/hmiyazawa1984/placozoan_mitogenome/src/master/: last accessed September 27, 2020). The annotated mitogenomes have been submitted to GenBank and respective accession numbers are given in table 1.

**Table 1:** Summary of General Characteristics of 14 Placozoan Mitogenomes

	*Clade*	Haplo Type	Whole mt-Genome		Coding Region		Intergenic Region		Group I Intron		Group II Intron		Small Inverted Repeat						Tandem Repeat						Citation: Accession Number
			Size (bp)	%GC	Size (bp)	%GC	Size (bp)	%GC	Size (bp)	No.	Size (bp)	No.	Size (bp)			No.			Size (bp)			No.			
													Total	CR	IGR	Total	CR	IGR	Total	CR	IGR	Total	CR	IGR	
*P.*		H0	23,462	32.92	17,690	32.18	5,772	41.30	2,299	4	41	1	192	114	78	15	8	7	343	70	273	5	3	5	Osigus et al. (2019): MH682141.1
*T.*	*I*	H1	43,079	47.01	20,276	38.21	22,803	54.95	5,129	8	80	2	5,862	1,167	4,695	429	104	326	596	71	525	16	1	15	Dellaporta et al. (2006): DQ112541.1 Burger et al. (2009): NC_008151.2
		H17	43,204	47.07	20,313	38.31	22,891	54.95	5,108	8	80	2	5,844	1,227	4,617	428	111	318	525	76	449	13	1	12	This study: LC460470
		H2	44,169	47.93	20,260	38.38	23,909	56.13	5,967	8	80	2	7,034	1,193	5,841	483	87	397	867	68	799	18	2	16	This study: LC460468
	*II*	H3	36,699	44.18	19,742	37.67	16,957	51.77	6,505	7	81	2	1,808	460	1,348	121	33	89	25	0	25	1	0	1	Signorovitch et al. (2007): DQ889458.1 Burger et al. (2009): NC_008834.2
	*III*	H8	32,661	39.22	19,528	36.03	13,133	43.95	4,891	8	40	1	1,748	791	957	115	51	64	168	42	126	6	1	5	Signorovitch et al. (2007): DQ889456.1 Burger et al. (2009): NC_008832.2
*H.*		H23	32,980	39.14	19,522	36.14	13,458	43.49	3,790	7	40	1	2,102	835	1,267	147	56	91	219	77	142	6	2	4	This study: MT957399
	*IV*	H19	31,779	40.23	19,460	36.92	12,319	45.46	4,700	7	82	2	2,433	724	1,709	162	46	118	60	34	26	1	1	1	This study: LC460471
	*V*	H13	36,537	40.32	19,719	36.18	16,818	45.17	4,065	8	41	1	2,916	748	2,168	196	47	151	236	69	167	5	2	4	Eitel et al. (2018)
		H15	36,676	40.40	19,719	36.24	16,957	45.24	3,883	8	41	1	2,793	746	2,047	188	47	143	439	104	335	8	3	6	Miyazawa et al. (2012): NC_015309.1
		H9	36,602	40.27	19,694	36.18	16,908	45.03	4,055	8	41	1	2,863	731	2,132	191	46	147	330	70	260	6	2	5	This study: LC460472
		H4	37,194	39.94	19,616	36.08	17,578	44.25	5,116	8	41	1	2,895	722	2,173	208	48	162	221	72	149	6	2	5	Signorovitch et al. (2007): DQ889457.1 Burger et al. (2009): NC_008833.2
	*VII*	H24	33,532	42.69	19,765	38.14	13,767	49.21	6,178	8	46	1	3,294	1,150	2,144	215	68	148	245	93	152	5	3	3	This study: MT957400
	*VI*	H11	35,188	38.61	19,412	36.12	15,776	41.67	4,209	6	121	3	1,673	539	1,134	106	34	74	94	0	94	2	0	2	This study: LC460469
	Average		35,983	41.42	19,623	36.63	16,360	47.33	4,707	7	61	2	3,104	796	2,308	215	56	160	312	60	252	7	2	6	
	S.D.		5,167	3.89	609	1.54	4,615	4.92	1,067	1	25	1	1,819	301	1,563	132	27	107	222	30	208	5	1	5	

Note.—P., *Polyplacotoma*; T., *Trichoplax*; H., *Hoilungia*; S.D.: standard deviation.

### Identification of Repeat Sequences

Short palindromic sequences were detected using the Palindrome program in the EMBOSS package v6.6.0.0 (minimum length of palindrome, 6; maximum length of palindrome, 100; maximum gap between repeated regions, 10; and number of mismatches allowed, 1) (Rice et al. 2000), and those with a stem length longer than the loop were selected. The identification of short palindromic sequences was performed on 14 placozoan mitogenomes and 11 nonplacozoan metazoan mitogenomes. The taxonomy (phylum and class) and GenBank accession numbers of the other metazoans were as follows: *Vazella pourtalesi* (Porifera; Hexactinellida; GU385217), *Oscarella pearsei* (Porifera; Homoscleromorpha; NC_035983), *Lubomirskia baicalensis* (Porifera; Demospongiae; GU385217), *Favites abdita* (Cnidaria; Anthozoa; NC_035879), *Alatina alata* (Cnidaria; Cubozoa; KJ452776-KJ452783), *Turritopsis dohrnii* (Cnidaria; Hydrozoa; KT020766), *M. leidyi* (Ctenophora; Tentaculata; NC_016117), *Beroe forskalii* (Ctenophora; Nuda; MG655622), *Xenoturbella japonica* (Xenacoelomorpha; Xenoturbellida; LC228485), *Drosophila melanogaster* (Arthropoda; Insecta; KY310613), and *Homo sapiens* (Chordata; Mammalia; MG649324). The selected short palindromic sequences were classified based on the six stem nucleotides adjacent to the loop. We considered short palindromic sequences detected at least ten times in a single mitogenome as “repeat” types. Hereafter, “repeat” type short palindromic sequences were treated as SIRs.

Tandem repeats were identified using the Tandem Repeat Finder program (matching weight, 2; mismatching penalty, 7; indel penalty, 7; match probability, 80; indel probability, 10; minimum alignment score, 50; and maximum period size, 500) (Benson 1999).

Dotplot analyses of placozoan mitogenomes were performed using the Dotmatcher program in EMBOSS package v6.6.0.0 (window size, 50; threshold, 100) (Rice et al. 2000).

### Phylogenetic Analyses, Genetic Distances, and Gene Evolution

The new haplotypes H23 and H24 were initially assigned to existing *Hoilungia* clades based on the analysis of their diagnostic 16S rDNA fragment (Voigt et al. 2004). Briefly, the 16S rDNA fragments of all published *Hoilungia* lineages were aligned with MAFFT v7.017 (Katoh and Standley 2013) as implemented in Geneious using the E-INS-i algorithm. Subsequent phylogenetic analyses were conducted with FastTree 2.1.5 (Price et al. 2010) (as implemented in Geneious) using the default settings (not shown), and results were afterwards verified by phylogenetic analyses using the complete 16S rRNA sequences (see below).

Phylogenetic analyses of 14 placozoan haplotypes were performed on different data sets: 1) 12 protein-coding genes (amino acids), 2) 12 protein-coding genes (nucleotide), 3) 24 tRNAs, 4) two rRNAs, and 5) concatenated nucleotide sequences of data sets 2–4. The *Trichoplax* and *Hoilungia* mitogenomes shared long (>100 bp) IGRs, and we also conducted phylogenetic analyses of 13 haplotypes from the two genera using the concatenated IGR sequences. The nucleotide sequences of each data set were aligned using MAFFT L-INS-i v7.221 with default parameters (gap opening penalty at group-to-group alignment, 1.53) (Katoh and Standley 2013), and phylogenetic analyses were conducted using RAxML v8.2.12 with 100 bootstrap replicates. A maximum-likelihood tree analysis based on the amino acid sequence of 12 proteins (data set 1) was conducted under the JTT model selected by the “PROTGAMMAAUTO” option, whereas phylogenetic analyses on the nucleotide sequences (data sets 2–5) were conducted under the GTR + GAMMA model (Stamatakis 2014). Using the concatenated alignment, the alternative tree topology of placozoan 16S haplotypes, which is in partial disagreement with the topology previously reported by Eitel et al. (2013), was statistically tested using the approximately unbiased (AU), Kishino–Hasegawa (KH), Shimodaira–Hasegawa (SH), weighted Kishino–Hasegawa (wKH), and weighted Shimodaira–Hasegawa (wSH) tests in CONSEL (Shimodaira and Hasegawa 2001).

The pairwise genetic distances (*p*-distances) of the nucleotide sequence of the four following data sets were calculated in MEGAX (pairwise deletion for gaps/missing data treatment option) (Kumar et al. 2018): data sets 1–3 correspond to data sets 2–4 (i.e., the concatenated nucleotide alignments of 12 protein-coding genes, 24 tRNAs, and 2 rRNAs, respectively), which were also used for the phylogenetic analyses (see above). The fourth data set in our pairwise genetic distance analysis was the whole mitogenome alignment generated using MAFFT E-INS-i v7.429 with default parameters (gap opening penalty at group-to-group alignment, 1.53) (Katoh and Standley 2013).

To detect the insertions and deletions in the gene sequences of placozoan mitogenomes, we aligned those of 14 placozoan haplotypes and 11 metazoan species (see above). The alignments of the amino acid sequences of 12 protein-coding genes and the nucleotide sequences of two rRNA genes were generated using MAFFT L-INS-i. The sequence lengths and GC contents were compared using a one-tailed Welch’s *t*-test and one-tailed Mann–Whitney *U* test in R, respectively. Group I introns between *cox1* exons 10 and 11 and the *nad5* introns were independently aligned using MAFFT L-INS-i, respectively.

## Results

### General Features

**Fig. 1:**
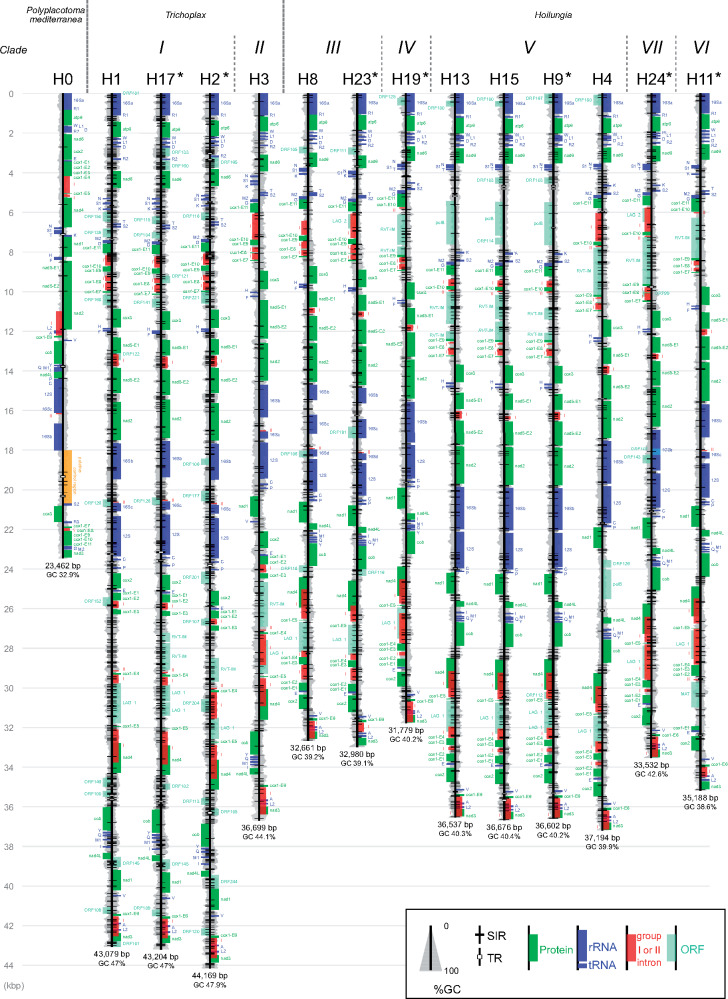
Linearized scaled maps of mitochondrial genomes of 14 placozoan haplotypes. The mitogenomes sequenced in this study are marked with asterisks. tRNA genes are indicated by one letter code for the corresponding amino acids. GC percentages (100-bp window) are indicated by the width of gray area. Abbreviations: SIR, small inverted repeat; TR, tandem repeat; ORF, open reading frame of unknown function.

The de novo assembly of our different data sets yielded seven new and complete circular mitochondrial genomes for H2, H9, H11, H17, H19, H23, and H24. Our 16S-based phylogenetic analyses allowed us to assign the new placozoan haplotypes H23 and H24 to *clades III* and *VII*, respectively. The characterization of the two new haplotypes increased the total number of reported placozoan haplotypes to 22 (i.e., H0–H19, H23, and H24). In agreement with the seven previously reported *Trichoplax* and *Hoilungia* mitogenomes, all newly determined mitogenomes encoded 12 protein-coding genes (*atp6*, *cob*, *cox1*-*3*, *nad1-6*, and *nad4L*), small rRNA (12S), large rRNA (16S), and 24 tRNAs (fig. 1). The molecular characteristics of these mitogenomes are summarized in table 1. H2 has the largest mitochondrial genome among placozoans (44,169 bp), whereas the smallest mitogenome was found in *P. mediterranea* (23,462 bp), followed by the newly sequenced H19 mtDNA (31,779 bp). The standard deviation of the length of the IGRs (4615.16) was larger than that of the coding regions (609.03). The GC contents of the *Trichoplax* haplotypes (H1, H17, H2, and H3) were all above 44.1%, whereas those of the *Hoilungia* haplotypes were all below 42.7%, and that of *Polyplacotoma* was 32.9%. The IGRs had GC contents ranging from 41.3% to 56.1%, which is markedly higher than that of the coding regions (32.1–38.4%). The sizes and GC contents of the whole mitogenome, IGRs, and coding regions of *P. mediterranea* were all smaller than those of the other haplotypes.

The order of typical mitochondrial protein-coding, rRNA, and tRNA genes (which omit ORFs of unknown function) in the 13 sequenced *Trichoplax* and *Hoilungia* mitogenomes was identical at the intraclade level, but not at the interclade level (figs. 1 and 4). In *clade I* of *Trichoplax*, the gene orders of H2 and H17 were identical to those of H1 (Dellaporta et al. 2006; Burger et al. 2009). In *Hoilungia*, the gene order of H23 was consistent with that of H8 (both *clade III*) (Signorovitch et al. 2007). Within *clade V*, haplotype H9 shared a similar gene order with H4, H13, and H15 (Signorovitch et al. 2007; Miyazawa et al. 2012). The gene orders in H19 and H24 mitogenomes, the first completely sequenced genomes in clades *IV* and *VII*, respectively, were identical to those in the mitogenomes of *clade V* in *Hoilungia* (figs. 1 and 4). The gene order of H11, the only known haplotype in *clade VI*, was similar to that of *clade III* (including the shared orientation of the trnT–trnK fragment when compared with the *Trichoplax* group).

### Fragmentation Patterns of *cox1*, *nad5*, and 16S

**Fig. 2:**
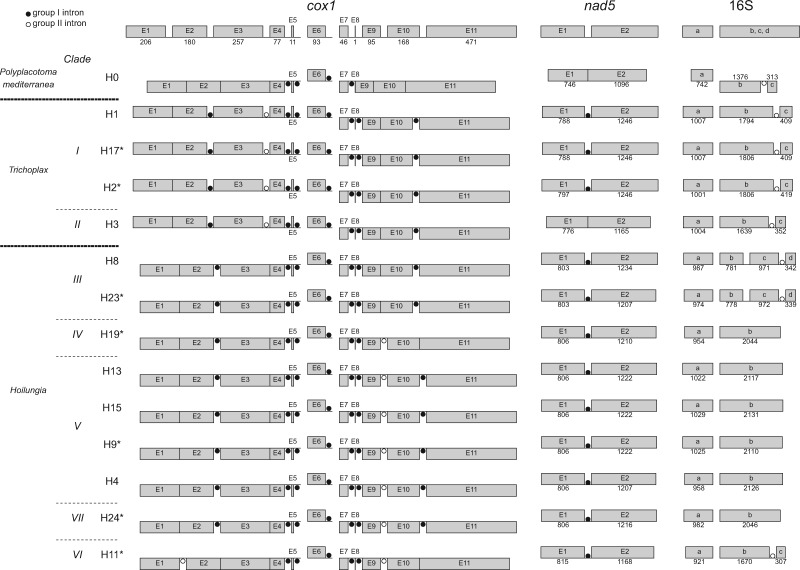
Fragmentation patterns of *cox1, nad5*, and 16S in placozoan mitogenomes. The mitogenomes sequenced in this study are marked with asterisks. Exons above and below the line for each haplotype are transcribed in the left–right and right–left direction, respectively. The general exon structure of the respective genes in placozoans is shown at the top. In *cox1*, each exon length is shown on the top, and in *nad5* and 16S, they are indicated above or below each exon, respectively. Group I and group II introns are indicated by black and white dots, respectively. The exons in 16S are labeled according to Signorovitch et al. (2007).

Although the total length of the *cox1* coding sequence is identical within the phylum Placozoa, the fragmentation patterns of this gene are diverse among clades (Signorovitch et al. 2007) (fig. 2). The *cox1* fragmentation pattern of the newly sequenced H2 and H17 was identical to that of H1 (all *clade I*), whereas that of H23 was consistent with that of H8 (likewise *clade III*) and that of H9 was the same as that of H4, H13, and H15 (*clade V*), implying that the fragmentation patterns are identical within each respective clade. The *cox1* fragmentation pattern of H24 (*clade VII*) was consistent with that of *clade V*. H19 (*clade IV*) had a similar pattern to *clade V*, except for exon 10 and 11, which were connected in H19. In H11 (*clade VI*), exon 1 and exon 2, which are connected in all other haplotypes, were separated by a group II intron containing an ORF. It is worth noting that some predicted introns, such as the intron near *cox1* exon 5 in H1, overlap with the coding sequence of neighboring genes.

A conserved group I intron was detected in the *nad5* gene of all *Trichoplax* and *Hoilungia* haplotypes, except for H3 (*clade II*) (figs. 1 and 2). This specific *nad5* intron, however, was also absent in *P. mediterranea*. The alignment of the placozoan *nad5* intron (supplementary fig. S1*b*, Supplementary Material online) revealed that 120 out of 1,733 positions were the same among the 12 haplotypes.

In the placozoan mitogenomes, the 16S rRNA gene was split into two major parts. With regards to *Trichoplax* and *Hoilungia*, one major part was found to be located between *nad3* and *atp6* (fragment “a” in fig. 2), whereas the other major part was located between *nad2* and 12S (fragments “b,” “c,” and “d” in fig. 2) (Dellaporta et al. 2006; Signorovitch et al. 2007; Miyazawa et al. 2012; Eitel et al. 2018). This characteristic was also observed in all seven newly sequenced mitogenomes (figs. 1 and 2). A group II intron further subdivided the part located between *nad2* and 12S in *clades I*, *II*, and *VI*, as well as haplotype H0 (fig. 1). However, this subdivision was absent in *clades IV*, *V*, and *VII*. A unique case of fragmentation was observed in *clade III*, where the part located between *nad2* and *12S* was subdivided into three exons.

### IGRs, Introns, and ORFs

Placozoan mitogenomes have been reported to possess multiple ORFs of unknown function in the IGRs (Dellaporta et al. 2006; Signorovitch et al. 2007; Miyazawa et al. 2012; Eitel et al. 2018). Although the seven newly sequenced mitogenomes were found to partly share already known ORFs, they were also found to code for additional ORFs (fig. 1 and supplementary table S2, Supplementary Material online).

In the newly sequenced mitogenome of H9, an ORF (*polB*), which was previously detected between trnT and trnK in H13 and H15 (fig. 1) (Miyazawa et al. 2012; Eitel et al. 2018), and between *nad1* and *nad4L* in H4, was found between trnT and trnK (fig. 1 and supplementary fig. S2, Supplementary Material online; blue circles), with high a sequence similarity to the corresponding ORF in H15 (BlastX search, 98% identity) (supplementary table S2, Supplementary Material online). An ORF containing a reverse transcriptase domain and a group II intron maturase domain (RVT-IM), which had been previously identified between *cox1* exons 9 and 10 in the mitogenome of *clade V* (H4, H13, and H15) (fig. 1; Signorovitch et al. 2007; Miyazawa et al. 2012; Eitel et al. 2018), was found at a similar position between *cox1* exons 9 and 10 in the mitogenomes of H11, H19, and H24 (fig. 1). ORFs showing high similarities to group I intron-encoded LAGLIDADG endonuclease domains were found between *cox1* exons 4 and 5 (named LAG_1 in fig. 1 and supplementary table S2, Supplementary Material online) in the mitogenomes of H11, H19, H23, and H24. Similar ORFs have previously been reported in the mitogenomes of H1, H3, H4, H8, and H15 (Signorovitch et al. 2007; Miyazawa et al. 2012).

An overview of previously unknown ORFs is provided in figure 1 and supplementary table S2, Supplementary Material online. Briefly, the H11 mitogenome harbors a group II intron between *cox1* exons 1 and 2 (figs. 1 and 2), and the ORF in this intron (a putative MAT; *cox1I1a* maturase) showed similarity in BlastX searches (supplementary table S2, Supplementary Material online) with the mitochondrial-encoded reverse transcriptase found in *Halamphora coffeaeformis* (Chromista). The mitogenome of H2 was ∼1,000 bp longer than that of H1 and H17, respectively, mostly due to the presence of orf201 found between trnP and *cox2* (fig. 1). Similarity searches of orf201 using NCBI BlastX showed a low score (bit score, 37.7; identity, 80%), but a recognizable similarity was still observed with the respective alignment to the orf126 located between *nad1* and *nad4L* in H4 (fig. 1). A previously unknown LAGLIDADG endonuclease candidate was detected in this region in H23 (LAG_2) and H24 (LAG_2) (fig. 1), respectively. Interestingly, LAG_2 of H23 and H24 showed high similarities to the intron sequence between *cox1* exons 10 and 11 of H4 in BlastN searches (*e*-value, 0.0 and 4e-153, respectively; see supplementary fig. S1*a*, Supplementary Material online), but not with the nucleotide sequence of LAG_1 in H4.

### Gene Cluster and Gene Order Analyses

The availability of mitochondrial genome data from all placozoan genera and clades allowed us to conduct the first comprehensive analysis of mitochondrial gene order evolution in Placozoa. Our pairwise comparison of placozoan mtDNAs (omitting nonconserved mitochondrial ORFs) revealed the existence of 14 placozoan mitochondrial gene sections (named A–N in fig. 4). These sections comprise five single-gene sections, as well as nine multigene-gene clusters (fig. 4). The gene linkages within the clusters are shared between all the placozoan genera and clades. Given the fragmentation of the placozoan 16S rRNA and *cox1* genes, several different sections/clusters may comprise partial fragments of respective genes, which in turn potentially comprise one or multiple exons. In order to analyze the evolution of mitochondrial gene arrangements in placozoans, changes to gene cluster sequences were mapped to the placozoan phylogenetic tree (fig. 4). *Polyplacotoma mediterranea* possesses a highly unique mitochondrial gene order, but nevertheless shares the block B–C* (fig. 4; depicted by dotted lines) with *clades IV*, *V*, and *VII* (all *Hoilungia* group). On the other hand, several long and conserved blocks could be identified between and within the *Trichoplax* and *Hoilungia* groups, respectively. Briefly, the blocks M–A–B and D–E–F–G–H are shared between all members of the *Trichoplax* and *Hoilungia* groups. Within the *Trichoplax* group, the block M–A–B–C–D–E–F–G–H and block I–J (fig. 4; the latter depicted by dotted lines) are shared between *clades I* and *II*. The former block M–A–B–C–D–E–F–G–H, however, can also be found in *clades VI* and *III* in the *Hoilungia* group. *Nad1* (section K*) in *clade II* and trnV (section L) in *clade I* share their relative positions with all members of the *Hoilungia* group, but not with their respective counterparts within the *Trichoplax* group. Within the *Hoilungia* group, block D–E–F–G–H–K*–J*–I*–L–M–A–B is shared between all clades. Furthermore, *clades VI* and *III* share an overall identical cluster sequence, and so does the subgroup formed by *clades IV*, *V*, and *VII*.

### Short Repetitive Motifs

Our dotplot analyses revealed the presence of short repeat sequences in the placozoan mitogenomes (supplementary fig. S2, Supplementary Material online). These short repeat sequences were abundant and shared especially between haplotypes in *clade I* (H1, H2, and H17) (table 2). On the other hand, H3 (belonging to *clade II*, the sister group to *clade I*) had many fewer short repeat sequences than haplotypes in *clade I*, and only a few repeat sequences were shared between these two clades (supplementary fig. S2, Supplementary Material online, and tables 1 and 2). In *clade V*, short repeats were shared between all of the haplotypes characterized so far (i.e., H4, H9, H13, and H15). Remarkably, the *clade V* haplotypes also shared short repeats with H24 (*clade VII*) (supplementary fig. S2, Supplementary Material online). In *clade III*, fewer short repeats were observed, but many of them were shared between H8 and H23 (supplementary fig. S2, Supplementary Material online). Compared with *clades I*, *V*, and *VII*, all the other clades contained fewer short repeats (supplementary fig. S2, Supplementary Material online).

**Table 2:** SIRs in 14 Placozoan and 11 Nonplacozoan Mitogenomes

		6 Nucleotides of Stem on the Gap Side	The Number of SIR																				
			Placozoa										Fungi	Porifera			Ctenophora	Cnidaria			Bilateria		
			H1	H17	H2	H3	H8	H19	H4	H9	H15	H11	*Monosiga brevicollis*	*Vazella pourtalesi*	*Oscarella pearsei*	*Lubomirskia baicalensis*	*Mnemiopsis leidyi*	*Metridium senile*	*Alatina alata*	*Turritopsis dohrnii*	*Xenoturbella japonica*	*Drosophila melanogaster*	*Homo sapiens*
Six stem nucleotide sequences adjacent to the loop	Types of SIR identified at least ten on placozoan mitogenome	AAAAAA	1	0	1	4	15	12	10	4	4	4	5	0	1	0	1	1	0	2	0	0	0
		AAAGAT	9	11	13	0	0	0	1	2	1	1	6	0	0	0	0	0	0	2	0	1	0
		CAAAAG	8	8	13	2	3	2	2	3	3	3	0	0	0	0	0	0	0	1	1	0	0
		CCGTAC	22	24	8	1	1	1	0	0	0	0	0	0	0	0	0	0	0	0	0	0	0
		CGAACG	0	0	0	23	0	0	0	0	0	0	0	0	0	0	0	0	0	0	0	0	0
		CGCCCC	2	2	2	2	0	0	2	11	9	8	0	0	0	0	0	0	0	0	0	0	0
		GAACCC	1	1	1	1	0	1	12	0	0	0	0	0	0	0	0	0	0	0	1	0	0
		GAATCC	2	2	2	0	0	5	11	11	14	12	0	0	0	0	0	0	0	1	0	0	0
		GAGCCC	4	6	5	1	0	3	5	16	17	16	0	0	0	0	0	0	0	0	0	0	0
		GATCCA	13	13	21	2	0	2	0	0	0	0	0	0	0	0	0	0	0	0	0	0	0
		GCGCCC	8	8	20	2	1	1	1	6	7	7	0	0	0	0	0	0	0	0	0	0	0
		GCGCCG	9	5	13	2	0	0	0	4	3	2	0	0	0	1	0	0	0	0	0	0	0
		GGACCC	6	6	3	1	2	5	15	10	11	12	0	0	0	0	0	0	0	0	0	0	0
		GGATCC	53	47	48	9	26	24	32	31	25	24	0	0	0	0	0	0	0	0	0	0	0
		GGCGCC	155	160	207	26	7	7	12	40	43	39	0	0	0	1	0	0	0	0	0	0	0
		GGGCCC	31	29	35	11	3	4	2	3	4	6	0	0	0	0	0	0	0	0	0	0	0
		GGGGGG	18	12	16	5	5	0	3	20	9	11	0	0	0	0	0	0	1	0	0	0	0
		GTCTAG	11	9	2	0	0	0	0	0	0	0	0	0	0	0	0	0	0	0	0	0	0
		TACCGT	12	13	5	0	0	0	0	0	0	0	0	0	0	0	0	0	0	0	0	0	0
		TTCGGC	10	10	12	0	1	0	0	0	0	0	0	0	0	0	0	1	0	0	0	0	0
		TTGGGG	23	28	24	2	3	1	0	0	1	1	0	0	0	0	0	2	0	0	0	0	0
		TTTTTT	9	9	8	11	3	6	13	11	10	10	8	1	0	0	3	1	0	1	0	2	0
		AAAAAT	5	5	6	5	15	9	7	5	5	5	48	1	0	0	0	2	0	2	0	4	0
		TTTTAA	5	5	5	4	4	8	11	10	8	8	17	1	2	3	3	0	0	1	0	2	0
		TTTTTA	3	5	7	4	9	11	12	9	11	10	37	4	1	2	4	1	0	1	0	2	0
		AAAATA	2	2	2	0	5	4	0	1	1	1	36	2	1	1	2	1	0	0	2	5	0
		AAAATT	3	3	4	4	9	4	3	2	1	2	58	2	0	1	2	2	0	0	0	5	0
		AAATAA	2	2	2	3	3	1	2	3	5	5	25	2	0	1	0	1	8	1	1	4	0
		AAATAT	0	0	0	0	2	0	2	0	1	1	44	2	1	1	1	0	0	0	1	3	0
		AAATTA	0	0	0	1	0	2	0	0	0	0	52	2	0	0	1	1	16	3	1	3	0
		AAATTT	0	0	0	0	1	1	0	1	1	1	36	3	1	0	2	2	0	1	1	4	0
		AAGTAA	1	1	1	1	1	0	1	1	1	1	1	1	0	0	0	0	16	0	0	1	0
		AATAAA	6	6	5	3	2	1	1	3	1	1	38	1	2	2	6	0	1	1	0	5	1
		AATAAT	4	4	4	4	5	5	6	9	7	7	48	5	1	0	2	1	1	1	1	7	1
		AATATA	1	1	1	1	2	0	0	0	0	0	34	0	2	0	2	1	0	1	0	0	0
		AATATT	7	7	7	3	2	4	4	5	4	4	55	3	3	3	2	0	1	1	0	2	0
		AATTAA	0	0	0	0	0	2	1	1	1	0	40	1	0	0	0	1	0	2	1	2	0
		AATTAT	2	2	2	1	3	3	3	3	2	2	45	0	2	2	6	1	0	3	1	4	0
		AATTTA	1	1	1	3	3	1	1	4	4	4	69	2	1	1	4	1	8	0	0	3	0
		AATTTT	0	0	0	1	2	4	4	7	5	5	27	0	1	0	7	1	0	1	3	5	0
		ACCGAC	0	0	0	0	0	0	0	0	0	0	0	0	0	16	0	0	0	0	0	0	0
		ATAAAA	1	1	1	3	1	9	4	4	4	4	46	2	1	0	3	2	0	1	0	4	0
		ATAAAT	0	0	0	1	0	2	1	4	2	2	65	1	0	2	2	0	1	2	0	4	0
		ATAATA	1	3	2	1	3	3	1	6	4	4	51	2	2	1	1	0	1	1	0	1	0
		ATAATT	0	0	0	1	4	4	2	1	1	1	53	1	2	1	5	0	0	3	0	4	0
		ATATAA	1	1	1	0	1	2	1	2	1	1	47	0	2	0	2	1	1	0	0	2	0
		ATATAC	0	0	0	0	0	0	0	0	0	0	2	0	0	11	0	0	0	0	0	0	0
		ATATAT	1	1	1	1	3	2	2	3	4	4	27	2	1	0	1	1	0	1	0	1	0
		ATATCC	0	0	0	0	0	0	0	0	0	0	0	0	0	16	0	0	0	0	0	0	0
		ATATTA	4	4	4	3	2	4	3	7	6	7	44	1	3	3	1	0	2	0	1	4	1
		ATATTT	1	1	0	0	0	2	0	3	1	1	55	2	0	0	2	0	0	3	1	1	0
		ATTAAA	4	4	4	2	5	4	3	5	4	4	25	0	2	1	9	1	0	2	1	6	1
		ATTAAT	1	1	1	1	2	1	2	3	3	3	80	4	4	2	2	2	1	1	1	5	0
		ATTATA	3	3	3	4	1	1	1	2	1	2	40	2	0	3	1	0	0	1	0	3	0
		ATTATT	1	1	1	2	3	3	3	3	2	2	76	3	2	0	2	0	0	3	0	2	0
		ATTCCC	0	0	0	0	0	0	0	0	0	0	0	0	0	0	0	0	16	0	0	0	0
		ATTTAA	2	2	3	3	0	0	1	0	1	1	47	1	1	2	3	0	0	0	0	3	1
		ATTTAT	1	1	1	0	1	2	2	6	4	4	63	2	0	0	4	1	1	0	1	3	0
	Types of SIR identified at least ten on nonplacozoan mitogenome	ATTTTA	2	2	3	2	5	7	6	7	7	7	48	1	2	0	5	0	0	5	0	5	0
		ATTTTT	1	2	2	4	1	2	4	1	1	2	27	0	1	1	4	1	16	2	0	5	0
		CAGGAT	2	2	2	2	1	1	1	1	1	1	0	0	0	16	0	0	0	0	0	0	0
		CCACCT	0	0	0	0	0	0	0	0	0	0	1	0	0	27	0	0	0	0	0	0	0
		CTATGA	0	0	0	0	0	0	0	0	0	0	0	0	0	12	0	0	0	0	0	0	0
		GGCATC	0	0	0	0	0	0	0	0	0	0	0	0	0	21	0	0	0	0	0	0	0
		TAAAAA	1	1	1	1	2	6	3	2	3	3	28	2	3	0	4	0	1	4	1	4	0
		TAAAAT	2	2	2	3	2	1	2	2	2	2	44	0	2	0	2	1	8	3	3	3	0
		TAAATA	1	1	1	1	1	3	0	4	2	2	45	2	2	0	2	0	2	2	0	3	0
		TAAATT	1	1	0	0	1	1	0	1	1	1	42	1	2	14	2	0	0	2	0	4	0
		TAATAA	1	1	1	2	2	2	4	4	3	3	97	2	1	0	5	1	1	6	1	5	0
		TAATAT	3	3	2	2	2	2	1	3	3	3	58	2	3	1	3	0	0	2	0	3	0
		TAATTA	1	1	1	2	2	0	1	1	6	5	55	0	1	1	1	1	0	3	1	4	0
		TAATTT	2	3	2	2	1	2	1	4	2	2	34	0	0	0	3	1	0	1	0	4	1
		TAGTTT	1	1	1	1	1	1	1	1	1	1	11	4	0	0	0	0	0	0	1	0	0
		TATAAA	1	1	1	1	2	2	3	3	3	3	62	1	1	1	3	0	0	2	1	4	0
		TATAAT	4	4	4	1	1	1	2	5	4	4	53	2	1	0	1	2	1	0	0	1	1
		TATATA	3	3	3	5	4	6	6	8	8	8	52	2	1	1	2	0	0	0	0	0	0
		TATATT	2	2	3	1	0	0	0	1	0	0	53	0	2	2	3	1	0	3	0	0	0
		TATTAA	2	2	2	2	2	0	1	3	2	2	86	1	0	0	2	0	2	0	0	12	0
		TATTAT	4	4	4	3	3	5	4	6	8	8	37	2	2	0	4	2	0	1	0	1	0
		TATTTA	4	4	4	3	3	3	3	5	3	3	31	2	1	0	1	2	0	1	0	4	0
		TATTTT	6	6	5	3	2	3	6	4	5	5	28	2	0	0	4	0	8	1	1	2	0
		TTAAAA	2	2	2	2	3	2	1	3	1	1	26	1	2	0	1	2	0	0	3	7	1
		TTAAAT	3	3	3	2	0	2	0	3	1	1	41	0	0	1	5	0	0	1	0	5	0
		TTAATA	3	3	4	1	4	4	2	3	4	4	56	1	1	0	7	1	0	0	1	7	0
		TTAATT	1	1	1	1	0	2	0	0	1	1	48	1	1	1	3	0	0	3	0	3	1
		TTATAA	1	1	1	0	0	2	0	3	2	2	63	4	2	1	6	1	0	2	2	2	0
		TTATAT	3	3	2	4	1	1	1	3	2	2	36	1	1	1	4	0	1	1	0	0	0
		TTATTA	1	1	1	1	0	2	1	4	1	2	41	0	0	1	3	0	1	2	0	6	1
		TTATTT	6	7	5	5	2	2	2	2	2	2	36	1	1	2	2	0	0	5	0	3	0
		TTTAAA	1	1	2	3	5	3	3	7	3	3	36	0	0	1	2	0	0	1	1	2	0
		TTTAAT	3	3	3	2	3	3	2	3	3	3	49	1	0	4	5	1	1	6	0	9	1
		TTTATA	2	0	0	1	6	4	2	0	1	1	53	1	3	0	7	1	0	2	1	1	0
		TTTATT	4	5	5	4	1	2	7	4	5	5	32	1	0	0	1	0	0	2	0	10	0
		TTTTAT	2	3	4	0	3	10	4	3	4	3	27	1	1	2	5	0	1	3	0	6	0
		*Total*	546	549	605	232	230	260	279	389	352	348	2886	95	76	188	188	47	118	110	36	232	11

Note.—Total numbers of SIRs at the bottom are the sum of all SIRs listed above.

Twenty-eight different types of SIRs were identified with more than ten occurrences in at least one of the 13 *Trichoplax* and *Hoilungia* mitogenomes, and their abundance varied greatly between haplotypes (table 2). The three haplotypes (H1, H2, and H17) in *clade I* had over 400 SIRs in their mitogenomes each, with H2 having the most (494). Type “GGCGCC” and “GGATCC” SIRs were observed at least ten times from 10 and 12 out of 13 haplotypes, respectively. Type “GGCGCC” SIRs were observed more than 150 times in all three haplotypes in *clade I*, whereas no other SIR type was found more than 100 times. On the other hand, SIR “CGAACG” was only observed in H3. The *P. mediterranea* mitogenome had the least number of palindromic sequences among placozoans, and no particular type of SIR was detected more than ten times (tables 1 and 2).

Among the 11 analyzed nonplacozoan metazoans, *Lubomirskia baicalensis* (Porifera; Demospongiae), *Beroe forskalii* (Ctenophora; Nuda), *M. leidyi* (Ctenophora; Tentaculata), *Alatina alata* (Cnidaria; Cubozoa), and *Drosophila melanogaster* (Arthropoda; Hexapoda) were found to contain specific types of short palindromic sequences more than ten times in their mitogenomes (table 2).

Tandem repeat sequences were also observed in placozoan mitogenomes, but their numbers and total lengths were much smaller than those of SIRs (table 1). The abundant SIR types “GGCGCC” and “GGATCC” (table 2) may also be detected as tandem repeats from their stem sequences, but most of them were undetectable using Tandem Repeat Finder (fig. 1 and table 1).

### Gene Length and GC Content in the Placozoan Mitogenomes

The lengths of *atp6*, *cob*, *cox2*, *cox3*, *nad1*, *nad2*, *nad3*, *nad4*, *nad5*, *nad6*, 16S, and 12S of the haplotypes in *Trichoplax* and *Hoilungia* were significantly (*P *<* *0.05) larger than those of other metazoans (supplementary fig. S3, Supplementary Material online). The lengths of *cox3*, *nad1*, *nad2*, *nad4*, *nad5*, *nad6*, 16S, and 12S of *P. mediterranea* were smaller than those of the other placozoan haplotypes (supplementary fig. S3, Supplementary Material online). The GC contents of *Trichoplax* and *Hoilungia atp6* were significantly (*P *<* *0.05) lower than those of other metazoans, whereas those of *nad2*, *nad6*, 16S, and 12S were significantly (*P *<* *0.05) higher (supplementary fig. S3, Supplementary Material online). The GC contents of *cob*, *cox3*, *nad1*, *nad2*, *nad4*, *nad5*, *nad6*, 16S, and 12S of *P. mediterranea* were smaller than those of the other haplotypes (supplementary fig. S3, Supplementary Material online). Indels containing SIRs were observed in the nucleotide alignments of some protein-coding and rRNA genes (supplementary fig. S4, Supplementary Material online), including SIRs starting from 70th, 635th, and 2,583th column of the alignment in *atp6*, *cox3*, and 16S, respectively.

### Phylogenetic Relationships of the 14 Placozoan Haplotypes

**Fig. 3:**
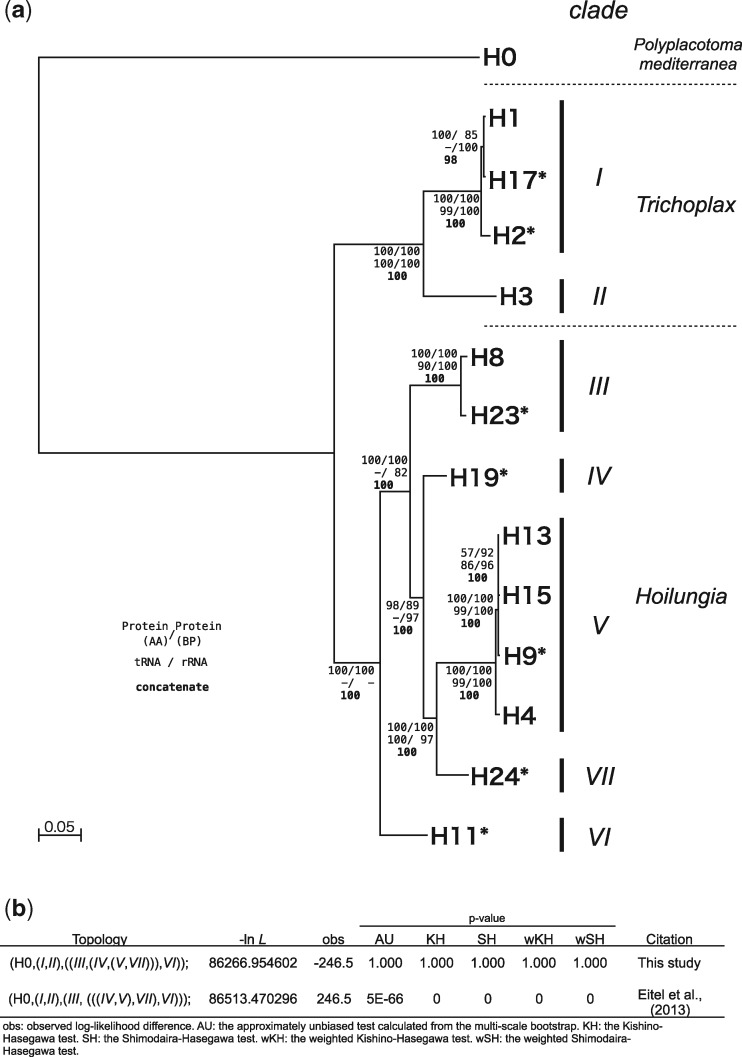
Phylogenetic relationships of 14 placozoan haplotypes based on complete mitogenome data. (*a*) Phylogenetic tree based on maximum-likelihood analyses of the concatenated alignment. Bootstrap support values for the different analyses are shown at each node. Mitogenomes newly sequenced in this study are marked with asterisks. (*b*) Statistical testing of the two major hypotheses on the relationships of haplotypes.

Phylogenetic analyses were performed using either the 12 mitochondrial protein-coding genes (amino acid or nucleotide), 24 tRNAs, two rRNAs, or the concatenated nucleotide sequence of all the above. Except for the analyses based on tRNAs and rRNAs, all phylogenetic analyses supported the same tree topologies (fig. 3*a* and supplementary fig. S5, Supplementary Material online), in which H11 (*clade VI*) was the sister group to all the other haplotypes in *Hoilungia*, instead of H8 + H23 (*clade III*). This topology conflicts with the 16S-based scenario, as previously suggested by Eitel et al. (2013). Except for the analyses based on tRNAs, *clade V* was the sister clade to *clade VII*. This is in disagreement with previous 16S-based studies, which supported *clade IV* as a sister to *clade V* (Eitel and Schierwater 2010; Eitel et al. 2013). The 16S-based phylogenetic tree topology suggested by Eitel et al. (2013) was rejected by statistical tests (AU, KH, SH, wKH, and wSH) implemented in CONSEL (*P *<* *0.01) (fig. 3*b*). *p*-Distance calculations of the four data sets (nucleotide alignments of 12 protein-coding genes, 24 tRNAs, two rRNAs, and whole mitogenomes, respectively) revealed *p*-distance values below 0.5 (supplementary fig. S6, Supplementary Material online), with values gradually corresponding to the observed phylogenetic relationships.

In the complete mitogenomes of the 13 *Trichoplax* and *Hoilungia* haplotypes (but not H0), eight neighboring gene pairs with IGRs longer than 100 bp were present (i.e., *cob*‒*nad4*, *cox1* exon 7‒*cox3*, *nad2*‒*nad5* exon 2, *nad2*‒16Sb, *nad6*‒trnR, trnF‒*nad5* exon 1, trnR‒trnD, and trnT‒trnK), which can be used to supplement the phylogenetic analyses of typical mitochondrial coding regions. Phylogenetic analyses based on the concatenated IGR sequences also strongly supported the relationship of the *Hoilungia* haplotypes, as described above (supplementary fig. S5*e*, Supplementary Material online).

## Discussion

### Phylogenetic Relationships among the Haplotypes

Based on the mitochondrial 16S sequences, 22 haplotypes have been reported from placozoans. The haplotypes were divided into three genera: *Polyplacotoma* with a single haplotype H0, *Hoilungia* with 17 haplotypes divided into five clades, and *Trichoplax* with four haplotypes divided into two clades (Eitel et al. 2013, 2018; Osigus et al. 2019; this study). Within *Hoilungia*, *clades IV*, *V*, *VII*, and *VI* have been suggested to form subgroup A2, whereas *clade III* has been suggested to form subgroup A1 (Eitel and Schierwater 2010; Eitel et al. 2013). Although the relationships of haplotypes within *clades I* and *V* were consistent compared with a previous study (Eitel et al. 2013), on the other hand, our phylogenetic analyses strongly support *clade VI* (consisting only of H11) as the sister to the remaining *Hoilungia* clades, rejecting the monophyly of subgroup A2 (fig. 3 and supplementary fig. S5, Supplementary Material online). In addition, our analyses support *clade VII*, not *clade IV*, as the sister clade to *clade V* (fig. 3 and supplementary fig. S5, Supplementary Material online).

In summary, the implementation of new mitochondrial molecular data resulted in a substantially improved phylogenetic framework for the phylum Placozoa. The most commonly used short 16S fragment (if used alone) possesses some severe weaknesses in robustly resolving placozoan relationships. Therefore, we recommend using the 16S fragment for haplotype identification and clade assignment only, whereas mitochondrial protein-coding genes should be used to infer placozoan relationships between clades or genera.

### Gene Fragmentation Patterns of *cox1, nad5*, and 16S

**Fig. 4:**
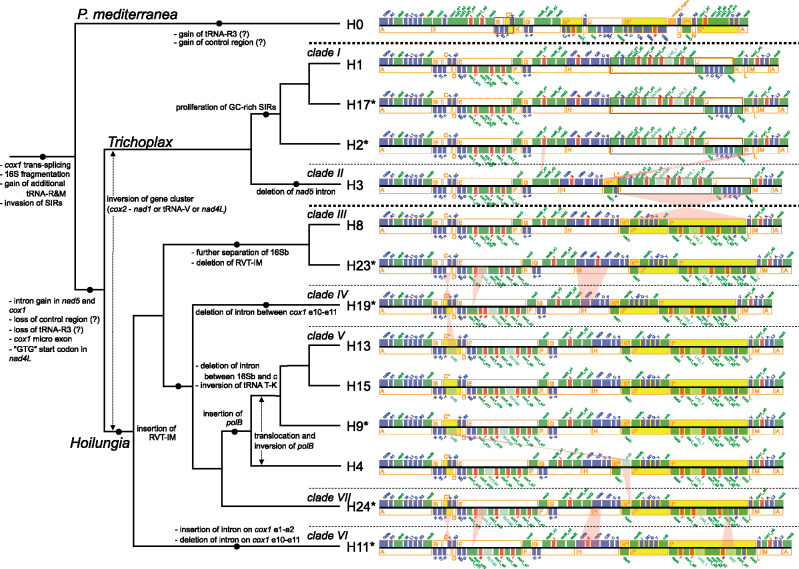
Summary of the putative succession of the evolution of placozoan mitogenome traits and gene orders. Inferred trait changes are mapped on the placozoan cladogram (left). Gene orders are shown on the right and rearrangements between neighboring haplotypes are indicated in pink. Mitochondrial genes linked in all known placozoans have been merged into gene sections A–N (orange) (see main text for details). Gene sections with identical gene content but with deviating gene orientations compared with *clade I* are indicated by an asterisk and highlighted in yellow. Specific blocks in H0, H1, H2, H3, and H17, which are explicitly discussed in the text, are enclosed by black dotted lines. Corresponding amino acids of tRNA genes are given as single letter amino acid codes.

Concerning *cox1* fragmentation patterns, an ORF-containing group II intron between exons 1 and 2 is only present in H11 (fig. 2). We therefore deduce that this insertion occurred exclusively in this lineage. Within *Hoilungia*, all clades except *clade III* possessed an ORF and a group II intron between *cox1* exons 9 and 10 (figs. 1 and 2). These ORFs all show high sequence similarities with the previously reported hypothetical protein in the H4 mitogenome at this site (RVT-IM in fig. 1, accession number: ABI53784.1) (supplementary table S2, Supplementary Material online). Given the placozoan phylogenetic relationships presented in this study, we deduce that this intron and ORF may be inserted in the mitogenome of the common ancestor of *Hoilungia* and may be secondarily lost in *clade III* (fig. 4). *Cox1* exons 10 and 11 are linked in H0, H11, and H19, whereas a group I intron is present in the other 11 mitogenomes (fig. 2). Because 168 out of 995 loci in the introns were identical among H4, H23, and H24 (supplementary fig. S1*a*, Supplementary Material online), and multiple regions were conserved among the 11 haplotypes (supplementary fig. S1*a*, Supplementary Material online), we consider that this intron may have been inserted in the common ancestor of *Trichoplax* and *Hoilungia* and was secondarily lost independently in H11 and H19 (fig. 4).

The presence of a *nad5* intron is shared by all haplotypes except H0 and H3. Some highly conserved intron regions (supplementary fig. S1*b*, Supplementary Material online) and the obtained tree topology (fig. 3) suggested the insertion of the *nad5* intron in the last common ancestor of *Trichoplax* and *Hoilungia* and a secondary loss of this intron in haplotype H3 (fig. 4).

The fragmentation of the 16S gene into two major fragments is one of the prominent characteristics of placozoan mitogenomes (e.g., Dellaporta et al. 2006; Signorovitch et al. 2007). A further subfragmentation due to the insertion of a group II intron within 16Sb has previously been reported in H0, H1, H3, and H8 (figs. 1 and 2) (Signorovitch et al. 2007; Burger et al. 2009; Osigus et al. 2019). This fragmentation pattern and the insertion of an intron were also observed in H2 and H17, belonging to *Trichoplax*, as well as in H11, the sister haplotype to the remaining *Hoilungia* haplotypes (figs. 1 and 2). Remarkably, *clades IV*, *V*, and *VII* mitogenomes lacked any intron in 16Sb (figs. 1 and 2), suggesting that this intron was lost in the common ancestor of these clades. Finally, the observed fragmentation of the 16S gene into four exons in *clade III* likely represents a highly derived state of this gene.

In summary, the multiple independent losses of introns observed in *cox1*, *nad5*, and 16S imply the selective secondary recompaction of the respective placozoan mtDNAs. Furthermore, it highlights the dynamic nature of intron evolution in the placozoan mitogenomes. Finally, it also highlights the importance of the implementation of multiple characteristics in comparative placozoan mitogenome analyses.

### Distribution of ORFs, Group I and II Introns, and IGRs

In the H4 mitogenome, an ORF showing similarity with a fungal DNA-directed DNA polymerase type B (*polB*) is present between *nad1* and *nad4L* (Signorovitch et al. 2007), whereas a similar ORF can be found between trnT and trnK in the H13 and H15 mitogenomes, suggesting that an inversion and translocation of this ORF occurred within *clade V* (Miyazawa et al. 2012; Eitel et al. 2018; blue circles in supplementary fig. S2, Supplementary Material online). The mitogenome of H9 had a similar ORF (BlastX *e*-value = 0.0) between trnT and trnK, identical in position and orientation with *polB* in H13 (fig. 1). Because the monophyletic relationship of H9, H13, and H15 within *clade V* is well supported (Eitel et al. 2013; fig. 3), this specific inversion and translocation likely occurred after the branching off of H4 within *clade V*.

In the same region between trnT and trnK, an inversion of about 1,000 bp was found within the IGR of H13 compared with H15 and H9 (red circles in supplementary fig. S2, Supplementary Material online). The short branch length leading to H13 (fig. 3) suggests a rather recent divergence of the H13 lineage that was accompanied by the IGR inversion. Because short (∼100 bp) sequences with high similarity were found at both ends of the inverted region (red arrows in supplementary fig. S2, Supplementary Material online), we deduce that a repeat-mediated intragenome recombination occurred in the H13 mitogenome.

The mitogenomes of the haplotypes in *clade V* were more than 3 kb larger than those of the only known members of the closely related *clade VII* (H24) (table 1). The most prominent difference between both clades is the presence of an ∼3.5 kb region containing a putative fungal DNA-directed DNA polymerase type B (*polB*), which is only found in *clade V* mitogenomes (fig. 1). A comparable increase in mitogenome size due to an ORF insertion has previously been suggested, for instance, in cnidarians (e.g., Chi et al. 2018). Accordingly, we suggest that the insertions of ORFs, introns, and IGRs contributed to the increase in mitogenome size in *clade V*, likely via horizontal gene transfer.

Another major finding of our study is the identification of a second type of a LAGLIDADG endonuclease candidate in a *cox1* intron in H23 (*clade III*) and H24 (*clade VII*) (LAG_2 in fig. 1 and supplementary table S2, Supplementary Material online). Given the phylogenetic relationships of the respective clades (fig. 3), the patchy occurrence of this second type of a LAGLIDADG endonuclease in only two clades indicates that this specific endonuclease was originally present in other *Hoilungia* clades but was secondarily lost in the course of evolution. However, more complete mitogenome data from *Hoilungia clades IV*, *V*, and *VI* are needed to test this hypothesis.

Finally, it should be highlighted that the functionality of some ORFs, which are predicted within the 16S rRNA gene of H23 and H24, respectively, have to be experimentally verified in future studies, because additional ORFs which are located within mitochondrial rRNA genes are highly unusual.

### Gene Cluster and Gene Order Analyses

Although mitochondrial gene order analyses are ill-suited to resolve deep metazoan relationships (due to missing data on ancestral gene orders in most animal phyla), they nevertheless are a powerful tool to infer evolutionary pathways within a phylum (e.g., Kayal et al. 2012). The nine provisional placozoan mitochondrial multigene clusters defined in this study (fig. 4) are shared between all of the characterized placozoans and therefore represent candidate gene clusters that were presumably present in the last common ancestor of modern placozoans. The complex fragmentation pattern of the intron-containing placozoan *nad5*, *cox1*, and 16S genes within these conserved gene clusters nevertheless highlights the exceptionally high dynamics of placozoan mitogenome evolution.

*Polyplacotoma mediterranea* possesses the most unique mitochondrial gene order, mirroring its sister relationships to all other extant placozoan haplotypes. This isolated position is also displayed by the presence of an additional unique tRNA (trnR3) (Osigus et al. 2019) (section N in fig. 4). However, the highly shuffled gene order does not allow us to draw any conclusions about the chronological order of rearrangement events in this species. Additional data from other *Polyplacotoma* species are needed to reconstruct the mitogenome evolution within this genus further (cf., Osigus et al. 2019). These data are also needed to clarify the extent to which the *Polyplacotoma* mitochondrial gene order represents an ancestral state within Placozoa. In any case, the data available for *P. mediterranea* reveal a conserved gene block (B–C*) (fig. 4), which can also be found in *clades IV*, *V*, and *VII*. Given the rather derived position of these clades, the sharing of this block between the distantly related taxa may be the result of multiple rearrangement events, which finally resulted in identical gene orders.

A pairwise comparison of the two clades of the *Trichoplax* group revealed that only the I–J block, as well as the *nad1* (K/K*) and the trnV (L/L*) sections, was reshuffled between both clades. Although only *clade I* (but not *clade II*) shared the relative position of trnV (section L) with *clade VI*, only *clade II* (but not *clade I*) shared the relative position of *nad1* (section K*) with *clade VI* (which is the sister clade to the remaining *Hoilungia* group and therefore was taken as a reference). However, we were unable to reconstruct whether the gene order of *clades I*, *II*, or *VI* (or none) potentially represents an ancestral gene order within the *Trichoplax*–*Hoilungia* group. Likewise, the gene order present in the last common ancestor of the *Trichoplax* group remains unclear. With a special focus on the *Hoilungia* group, the observed tree topology suggests that the gene order observed in both *clades VI* and *III* represents the ancestral gene order for this group. The only deviation from this gene order (i.e., the transition of cluster C to cluster C*) (fig. 4) likely occurred in the last common ancestor of *clades IV*, *V*, and *VII*.

Taken together, our results provide new and surprising insights into the evolution of mitochondrial gene orders in Placozoa. Furthermore, mitochondrial gene orders seem to be a valuable resource for future taxonomic approaches to define new systematic ranks in the phylum Placozoa.

### Effects of SIRs on the Placozoan Mitogenomes

Protein-coding genes have previously been reported to be longer in *T. adhaerens* (H1) than in other metazoans (Dellaporta et al. 2006). Our analyses using all available *Trichoplax* and *Hoilungia* data showed that *atp6*, *cob*, *cox2*, *cox3*, *nad1*, *nad2*, *nad3*, *nad4*, *nad5*, *nad6*, 16S, and 12S were significantly (*P *<* *0.05) longer in these genera than in other metazoans (supplementary fig. S3, Supplementary Material online). Our analyses also showed that the GC contents of *nad2*, *nad6*, 16S, and 12S were significantly higher than in other metazoans (*P *<* *0.05) (supplementary fig. S3, Supplementary Material online). Many insertions of SIRs into coding genes were detected in the *Trichoplax* and *Hoilungia* mitogenomes, such as SIRs starting from the 59th column in the *atp6* alignment and from the 607th column in the *nad6* alignment, as shown in supplementary figure S4, Supplementary Material online. Thus, we hypothesized that SIR insertions contribute to an increased length and GC content in the coding genes of these two genera.

The two new *clade I* mitogenomes sequenced in this study (i.e., from H2 and H17) were both more than 6 kb larger than the only known one from the sister clade, *clade II* (represented by H3). This observation suggests that a large mitogenome size (>43 kb) is an exclusive characteristic of *clade I* (fig. 1). Additionally, both the total size and number of SIRs in *clade I* were considerably larger than those in *clade II* (tables 1 and 2). In particular, the numbers of SIRs with stem sequences, such as “GATCCA,” “GGCGCC,” “GGATCC,” “GGGCCC,” and “TTGGGG,” were much higher in *clade I* than in any other haplotypes (table 2). Accordingly, the GC contents of the three mitogenomes in *clade I* were higher than those of other haplotypes (table 1). Therefore, we suggest that the proliferation of these GC-rich SIRs has contributed to the exceptionally large size and high GC contents of the mitogenomes in *clade I*.

Looking at the whole phylum, the *P. mediterranea* mitogenome is the smallest (23,462 bp) with the lowest GC content (32.9%) among placozoans (Osigus et al. 2019) (table 1). It lacks the GC-rich SIRs “GGCGCC,” “GGATCC,” and “GGGCCC,” which are present in the mitogenomes of all other haplotypes (table 2). In the 16S gene, the insertions of similar SIRs at the same position were detected in both the *Trichoplax* and *Hoilungia* clades, but not in *P. mediterranea* (SIRs starting from the 1,951th column in the alignment; supplementary fig. S4, Supplementary Material online). In *nad2*, the insertions of identical SIRs were observed in the three haplotypes, H9, H13, and H15 (SIRs starting from the 610th column in alignment; supplementary fig. S4, Supplementary Material online). In *cox3*, an insert containing an SIR was only present in H17 (SIRs starting from “G” at the 636th column in the *cox3* alignment; supplementary fig. S4, Supplementary Material online), suggesting that its insertion occurred after the separation of H17 and its closest relative H1. Similar examples can be found in the *Trichoplax* and *Hoilungia* mitogenomes (supplementary fig. S4, Supplementary Material online). These observations suggest that the proliferation of SIRs in the mitogenomes of the *Trichoplax* and *Hoilungia* lineages has occurred continuously since their divergence from *P. mediterranea* and have contributed to the increased sizes and the high GC content of these mitogenomes.

The absence of GC-rich hairpin structures belonging to the 5′-GGVBCC-(N)_3_-GGVBCC-3′ hairpin family in *P. mediterranea* in the first instance suggests a primary absence of this hairpin family in *Polyplacotoma.* However, GC-rich stem-loop structures can nevertheless sporadically be found in the *P. mediterranea* mitogenome. One such example is the variable stem-loop region in trnS (uga) (supplementary fig. S7, Supplementary Material online)*.* Remarkably, placozoans from *clades V* and *VII* possess at the corresponding position an intact 5′-GGCGCC-(N)_3_-GGCGCC-3′ hairpin in their trnS (uga) (supplementary fig. S7*b*, Supplementary Material online). We hypothesized that multiple independent insertions of different GC-rich hairpins at corresponding positions in the trnS (uga) of different placozoans are nonparsimonious, and therefore, unlikely. Instead, we deduced that an intact 5′-GGCGCC-(N)_3_-GGCGCC-3′ hairpin may have been integrated in the trnS (uga) of the last common placozoan ancestor before proliferating in some, but not all, placozoan taxa. The molecular mechanisms leading to the differential proliferation patterns of GC-rich hairpins in placozoans, however, await further investigation. In addition, it would be interesting to test the overall impact of SIRs, for instance, on the lifespan and aging (Yang et al. 2013) of different placozoan lineages. In mammalian mitogenomes, a strong negative correlation between the inverted repeat frequency and lifespan has been reported (Yang et al. 2013). If SIRs are selfish elements, their extensive proliferation may have negative effects on the fitness of placozoans. However, the placozoan haplotype H2, whose mitogenome possesses the most SIRs among placozoan mitogenomes (table 2), has the broadest global distribution (Eitel et al. 2013). Further studies are necessary to better understand the potential impact of SIRs on placozoan fitness and global distribution.

The evolutionary origin of numerous SIRs in the *Trichoplax* and *Hoilungia* mitogenomes remains unknown. However, it is possible that some of them originated from the putative control region of the last common ancestor of placozoans, because our analysis revealed several SIRs in the putative control region of the *P. mediterranea* mitogenome (fig. 1). Another possible scenario is that they originated from other organisms and were inserted into the placozoan mitogenomes via horizontal transfer. In any case, such profound proliferation of SIRs is a remarkable observation within metazoans. So far, similar characteristics have sporadically been reported, for example, from a few freshwater sponges (Lavrov et al. 2012), as well as from more distantly related taxa (see e.g., Shamanskiy et al. 2019). The complex occurrence and proliferation pattern of diverse SIRs in distantly related taxa indicate multiple independent, so far poorly characterized, underlying molecular mechanisms that will need to be investigated further.

The insertions and deletions in placozoan mitochondrial coding genes related to SIRs have so far only been studied between different 16S haplotypes within a clade (supplementary fig. S4, Supplementary Material online). It would be interesting to determine the extent to which variation caused by SIRs affects the overall genetic diversity within 16S haplotypes (i.e., at the population level). Cosmopolitan haplotypes, such as H2 and H4, are promising candidates for such comparative approaches, which may reveal hidden genetic diversity between global populations and provide a better genetic resolution at the respective intraspecific levels.

### Early Evolution of the Placozoan Mitochondrial Genomes

The first characterized placozoan mitogenome of *T. adhaerens* (H1) shares several characteristics with the mitogenomes of the choanoflagellates, the closest living relatives to metazoans, including substantial noncoding regions, large gene sizes, and several ORFs of unknown function, as well as introns (Dellaporta et al. 2006). Similar characteristics have also been reported in the placozoan haplotypes H3, H4, H8, H13, and H15 (Signorovitch et al. 2007; Miyazawa et al. 2012; Eitel et al. 2018) but were not found to this extent in other metazoans (Lavrov and Pett 2016). Taking into account traditional hypotheses on the early evolution of metazoans, it was deduced that the above-mentioned characteristics were present in the mitogenome of the last common ancestor of choanoflagellates and metazoans, and that placozoans are the only extant metazoan phylum to retain these characteristics (Dellaporta et al. 2006; Osigus et al. 2013). However, recent molecular phylogenetic analyses (e.g., Moroz et al. 2014; Pisani et al. 2015; Whelan et al. 2015; Simion et al. 2017; Laumer et al. 2018, 2019) have cast doubts on this scenario, as these studies do not support a sister group relationship between placozoans and all other metazoans.

The sister to all the other extant placozoan haplotypes, namely *P. mediterranea*, has recently been reported to possess a small mitogenome (23,462 bp) with only a small number of group I introns and short IGRs (Osigus et al. 2019) (figs. 1 and 2 and table 1). In the present follow-up study, we revealed that the proliferation of SIRs occurred in all placozoan haplotypes, but not in *P. mediterranea* (table 2). Taking into account the general mitogenome characteristics from all nonbilaterian phyla, our new interpretation of currently available mitogenome data suggests that the last common ancestor of metazoans possessed a small mitogenome unlike choanoflagellates, implying that the shared characteristics in choanoflagellate and derived placozoan mitogenomes are not retained from common ancestry. Accordingly, the last common ancestor of placozoans may have kept the small mitogenome of the last common metazoan ancestor, but insertions of ORFs, introns, and SIRs likely contributed to the expansion of the size and GC content of the mitogenomes in *Trichoplax* and *Hoilungia* after the branching off of *P. mediterranea*.

## Supplementary Material

Supplementary data are available at *Genome Biology and Evolution* online.

## Supplementary Material

evaa213_Supplementary_Data
