# Supplementary material for: Mitochondrial Genome Evolution of Placozoans: Gene Rearrangements and Repeat Expansions
Source: Genome Biol Evol. 2020 Oct 8;13(1):evaa213. doi: 10.1093/gbe/evaa213 (PMC7813641; doi:10.1093/gbe/evaa213)
Supplement: evaa213_Supplementary_Data [file evaa213_supplementary_data.zip › SuppleFigureTables_Legends.docx]

**Supporting Information Legends**

**Figure S1.** Alignments of non-coding regions. (a) Alignments of group I introns present in the intron between *cox1* exons 10 and 11. Loci shared by H4, H23, and H24 are denoted by “.”, while loci shared by all aligned haplotypes are denoted by “*”. (b) Alignments of the *nad5* intron. Loci shared by all aligned haplotypes are denoted by “*”.

**Figure S2.** Dotplot analyses of placozoan mitogenomes. The lower left corner in each box corresponds to the top of each mitogenome in Fig. 1. Blue circles indicate the inversion and translocation between H4 and H13, H15, or H9, respectively. Red circles indicate the inversion between H13 and H9 or H15. Red arrows indicate small repeat sequences with inversed directions.

**Figure S3.** Boxplot of length and GC contents of protein coding and rRNA genes. *P*-values are shown at the top of each gene. The lines within the boxes represent the median, and the boxes represent the 25^th^ and the 75^th^ percentiles of distribution.

**Figure S4.** Alignments of protein coding and rRNA genes. SIRs detected 10 times or more from at least one placozoan or non-placozoan mitogenome are enclosed in red and blue lines, respectively. Loci shared by all placozoan haplotypes are symbolized by “*”.

**Figure S5.** Phylogenetic analyses of placozoan haplotypes based on (a) amino acid sequences of protein coding genes, (b) nucleotide sequences of protein coding genes, (c) tRNA, (d) rRNA, and (e) long intergenic regions. Analysis using long intergenic regions (e) was conducted without *P. mediterranea* (H0), since the species lacks these regions.

**Figure S6.** Boxplots of pairwise genetic distances of coding regions (protein, tRNA, and rRNA), as well as whole mitogenomes in Placozoa. The “*Trichoplax”* boxplots indicate the distances between the four *Trichoplax* haplotypes, while the “*Hoilungia”* boxplots illustrate the distances between the nine *Hoilungia* haplotypes. The “ALL” boxplots indicate the distances between all 14 placozoan haplotypes from *Trichoplax*, *Hoilungia*, and *Polyplacotoma*, respectively. Red dots indicate the distances between *P. mediterranea* and the other 13 haplotypes. Green dots indicate distances between the *Trichoplax* and *Hoilungia* haplotypes. Blue dots indicate the distances between the haplotypes of different clades within the same genus. Purple dots indicate the distances between haplotypes within the same clades (applicable for *clade* *I* in *Trichoplax* and clades *III* and *V* in *Hoilungia*). The black horizontal lines within the white boxes represent the median, and the white boxes represent the 25^th^ and 75^th^ percentiles of distribution. (P: *Polyplacotoma*; T: *Trichoplax*; H: *Hoilungia*).

**Figure S7.** Secondary structure and alignments of the placozoan trnS (uga). (a) Secondary structure of trnS (uga) of H0 predicted by RNAfold web server. GC-rich stem-loop structure is enclosed in green lines. (b) Alignments of trnS (uga). GC-rich stem-loop structures of H0 and SIRs of other haplotypes are enclosed in green and red lines, respectively.

**Table S1.** List of primers used: (a) universal primers and (b) specific primers. See main text for details.

**Table S2.** Similarity search results of selected ORFs with unknown function in the newly sequenced placozoan mitogenomes. Only ORFs showing similarities with an e-value <1.0 are shown.
