# Supplementary material for: Mitochondrial Genome Evolution of Placozoans: Gene Rearrangements and Repeat Expansions
Source: Genome Biol Evol. 2020 Oct 8;13(1):evaa213. doi: 10.1093/gbe/evaa213 (PMC7813641; doi:10.1093/gbe/evaa213)
Supplement: evaa213_Supplementary_Data [file evaa213_supplementary_data.zip › SuppleFigureTables.pdf]

a) Alignments of group I intron present in the intron between *cox1* Exon10 and Exon11

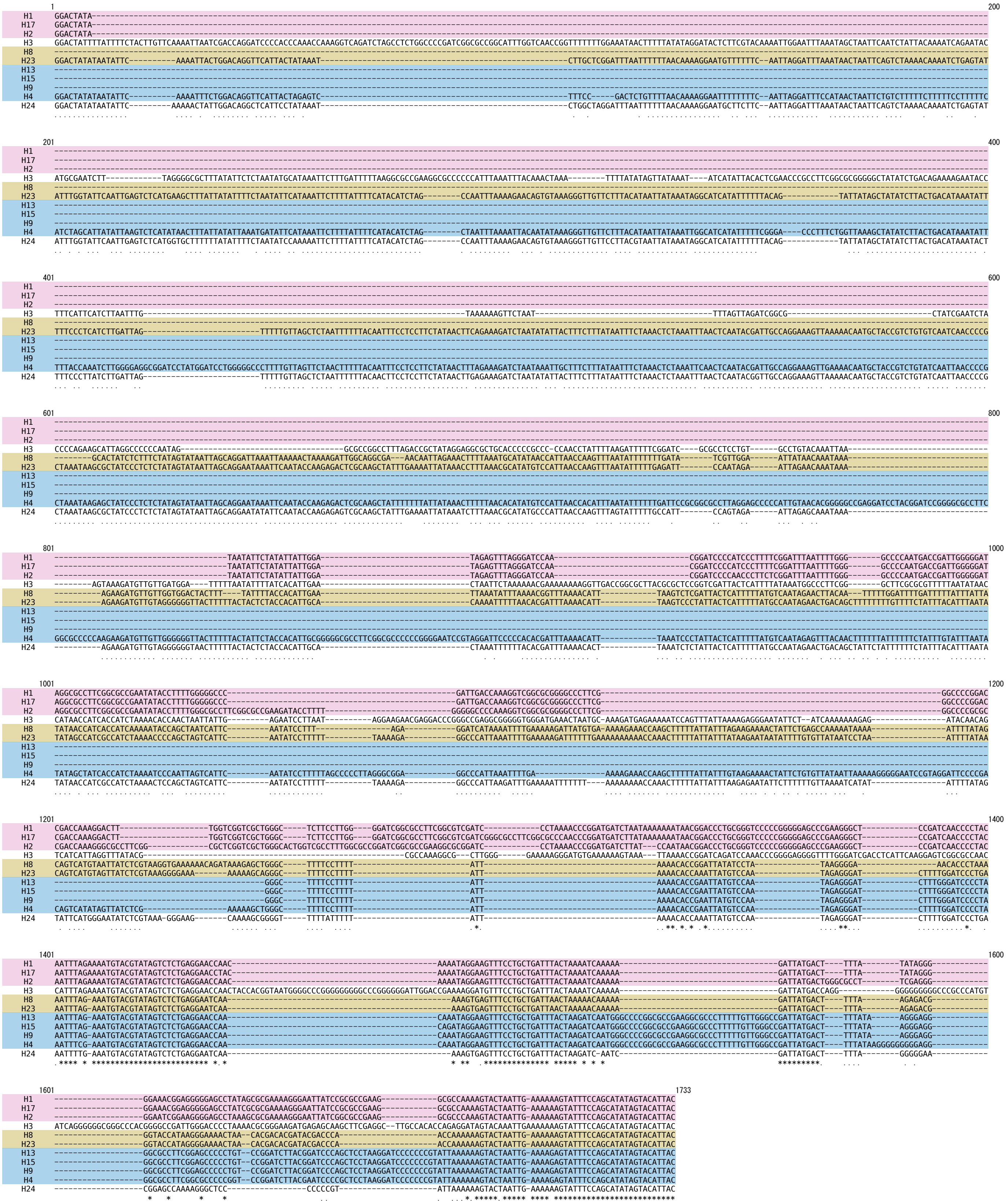

b) Alignments of the intron sequences between *nad5* exons

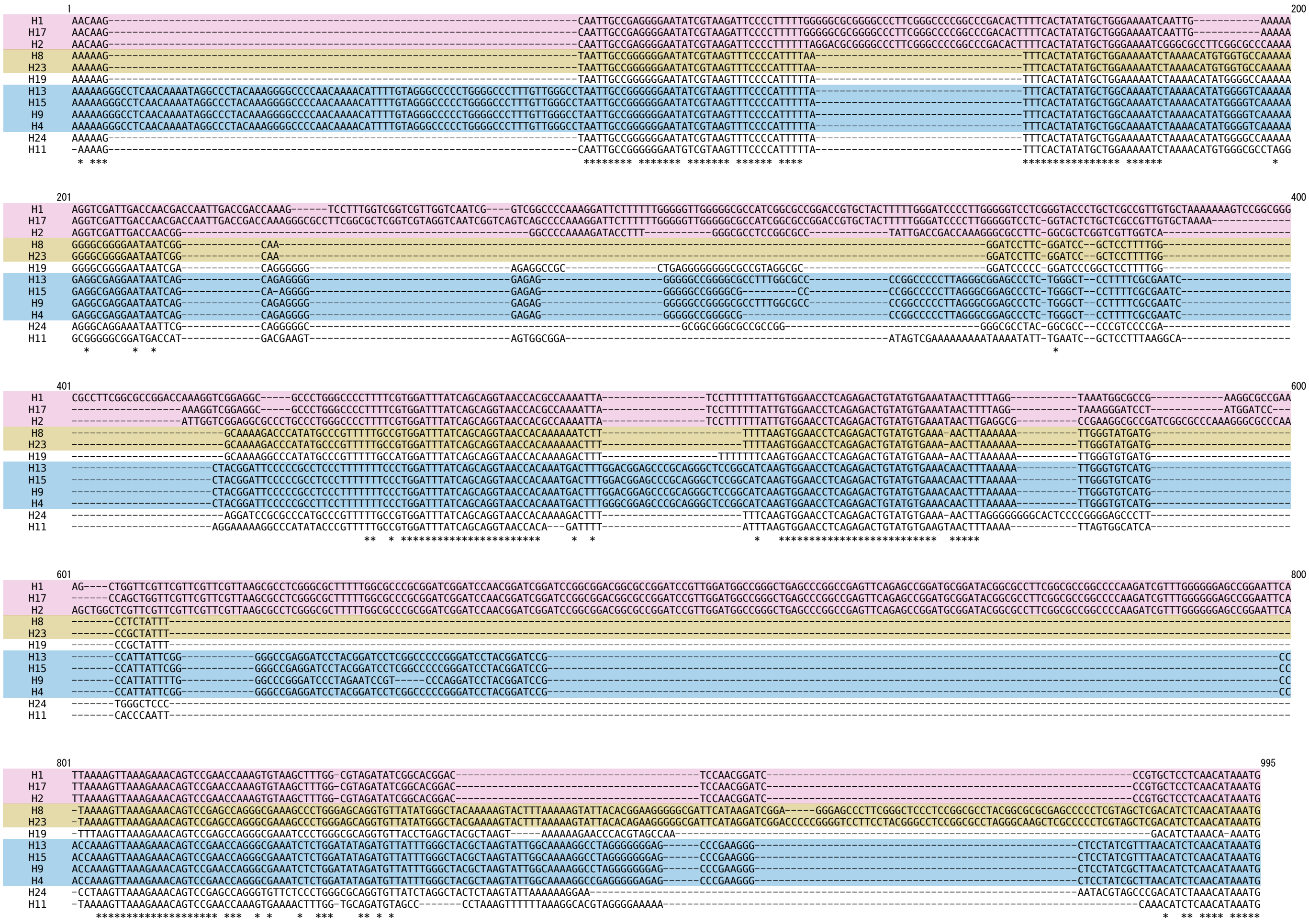

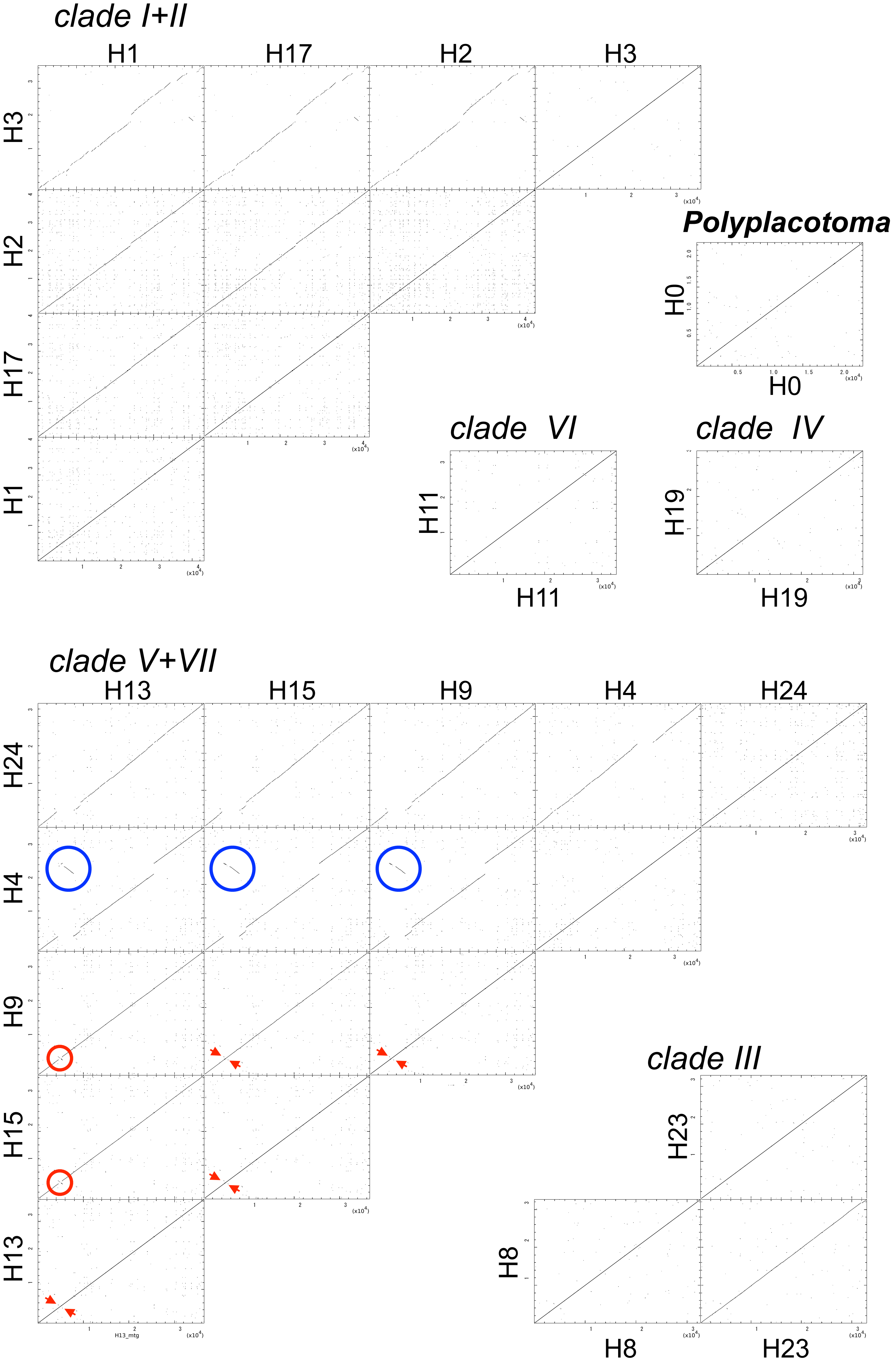
clade I+II
H1
H17
H2
H3
Polyplacotoma
H0
clade VI
H11
clade IV
H19
clade V+VII
H13
H15
H9
H4
H24
clade III
H23
H8
H13_mtg

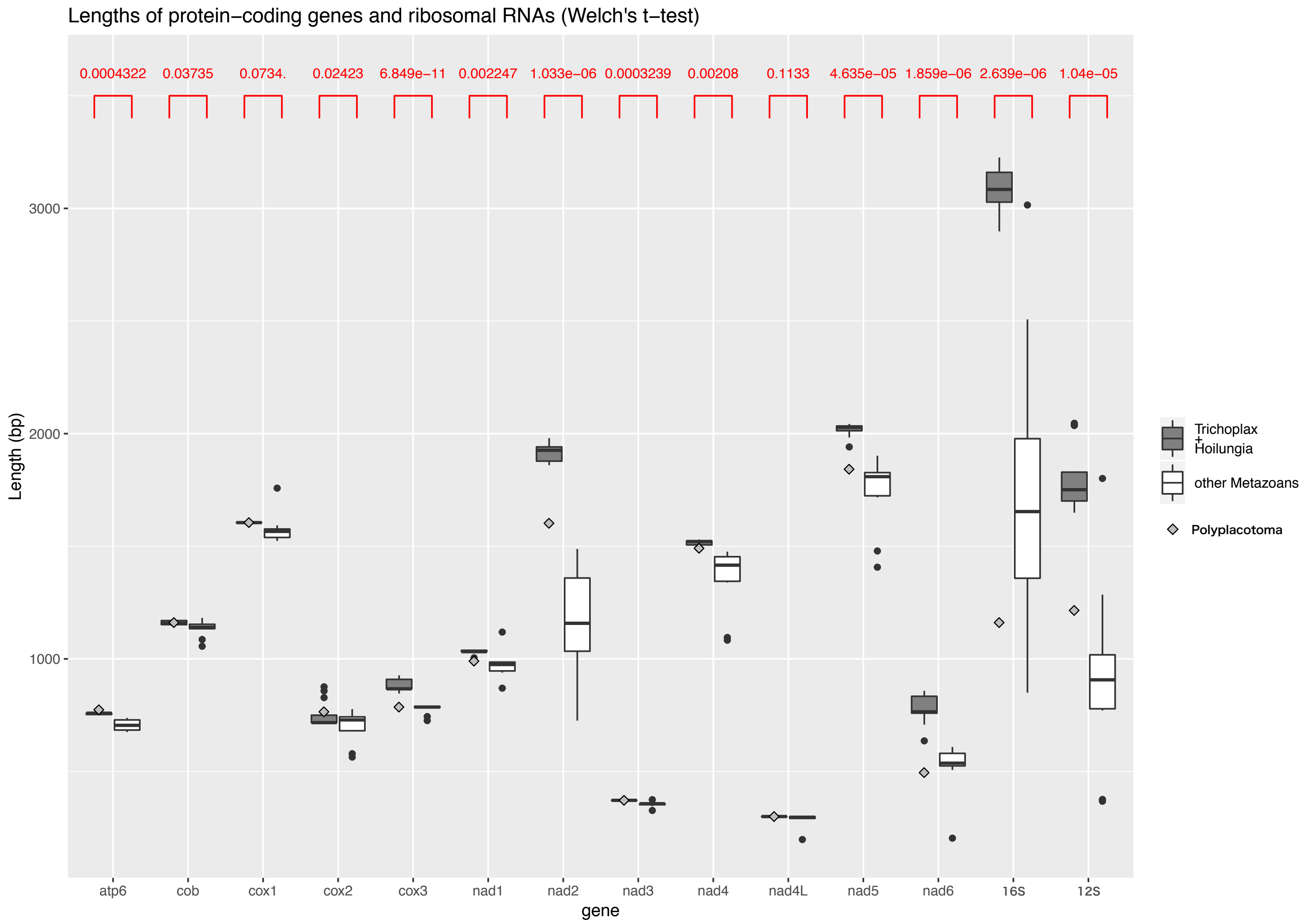
Lengths of protein−coding genes and ribosomal RNAs (Welch's t−test)
0.0004322
0.03735
0.0734.
0.02423
6.849e−11
0.002247
1.033e−06
0.0003239
0.00208
0.1133
4.635e−05
1.859e−06
2.639e−06
1.04e−05
Length (bp)
3000
2000
1000
atp6
cob
cox1
cox2
cox3
nad1
nad2
nad3
nad4
nad4L
nad5
nad6
16S
12S
gene
Trichoplax + Hoilungia
other Metazoans
Polyplacotoma

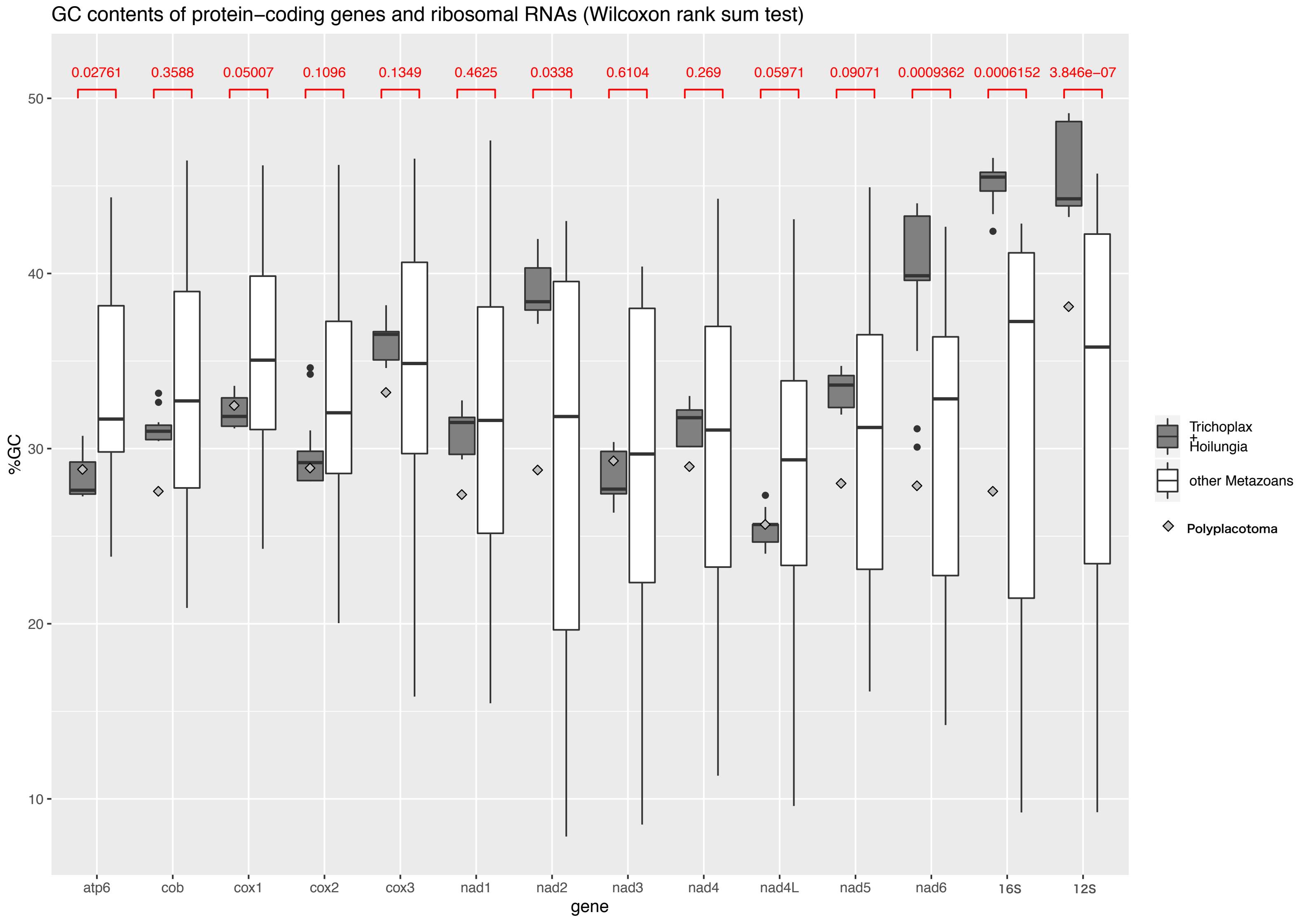
GC contents of protein−coding genes and ribosomal RNAs (Wilcoxon rank sum test)
0.02761
0.3588
0.05007
0.1096
0.1349
0.4625
0.0338
0.6104
0.269
0.05971
0.09071
0.0009362
0.0006152
3.846e−07
%GC
50
40
30
20
10
atp6
cob
cox1
cox2
cox3
nad1
nad2
nad3
nad4
nad4L
nad5
nad6
16S
12S
gene
Trichoplax + Hoilungia
other Metazoans
Polyplacotoma

cob

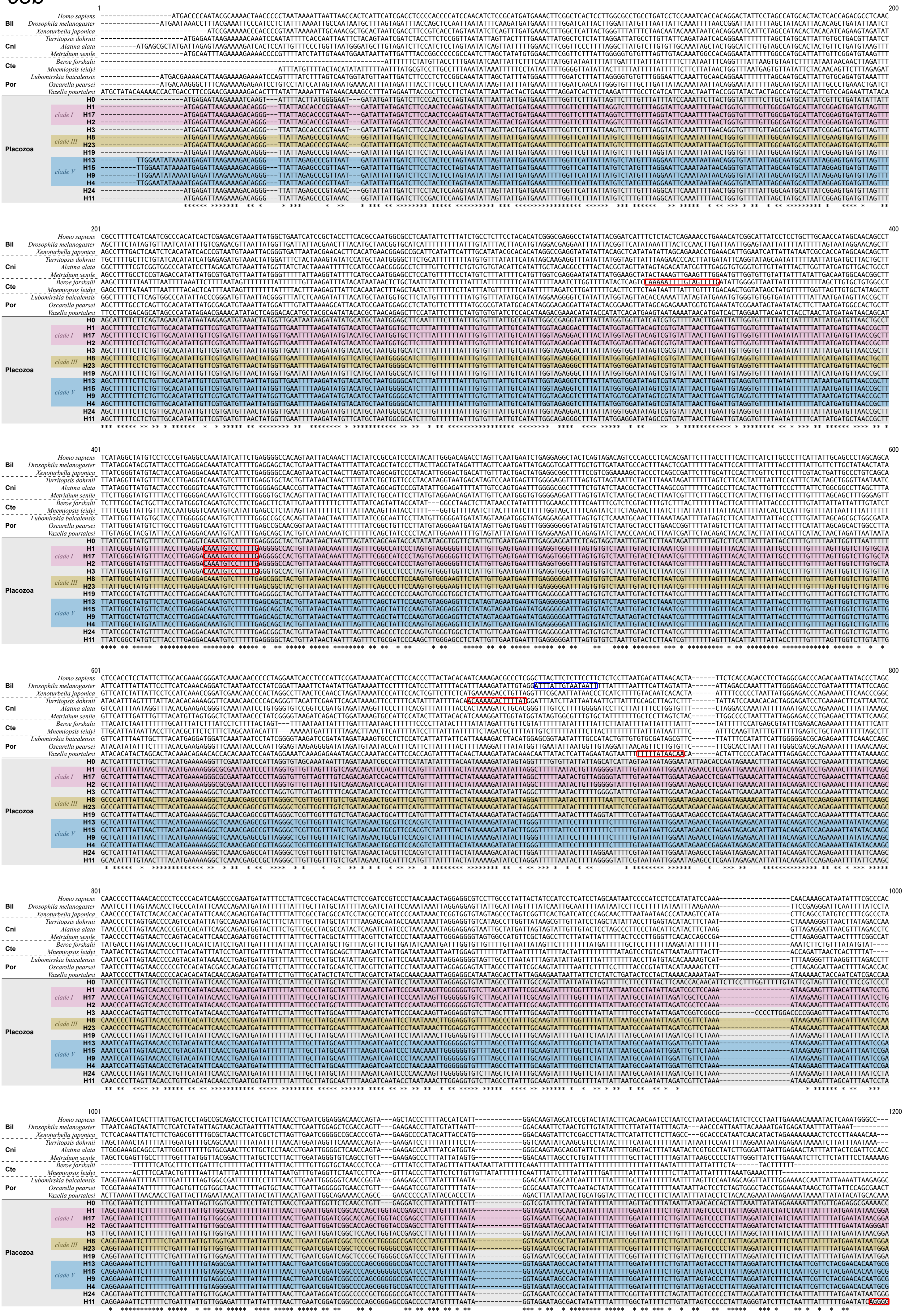

1
200
201
400
401
600
601
800
801
1000
1001
1200
Homo sapiens
Drosophila melanogaster
Xenoturbella japonica
Turritopsis dohrnii
Alatina alata
Metridium senile
Beroe forskalii
Mnemiopsis leidyi
Lubomirskia baicalensis
Oscarella pearsei
Vazella pourtalesi
Bil
Cni
Cte
Por
Placozoa
clade I
clade III
clade V
H0
H1
H17
H2
H3
H8
H23
H19
H13
H15
H9
H4
H24
H11

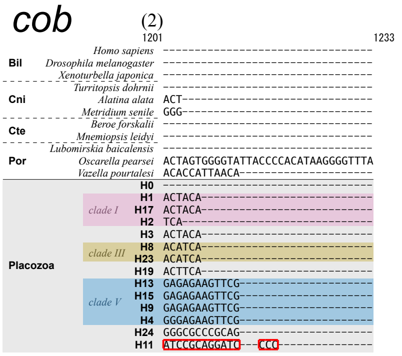
cob
(2)
1201
1233
Bil
Homo sapiens
Drosophila melanogaster
Xenoturbella japonica
Cni
Turritopsis dohrnii
Alatina alata
Metridium senile
Cte
Beroe forskalii
Mnemiopsis leidyi
Por
Lubomirskia baicalensis
Oscarella pearsei
Vazella pourtalesi
Placozoa
clade I
clade III
clade V
H0
H1
H17
H2
H3
H8
H23
H19
H13
H15
H9
H4
H24
H11

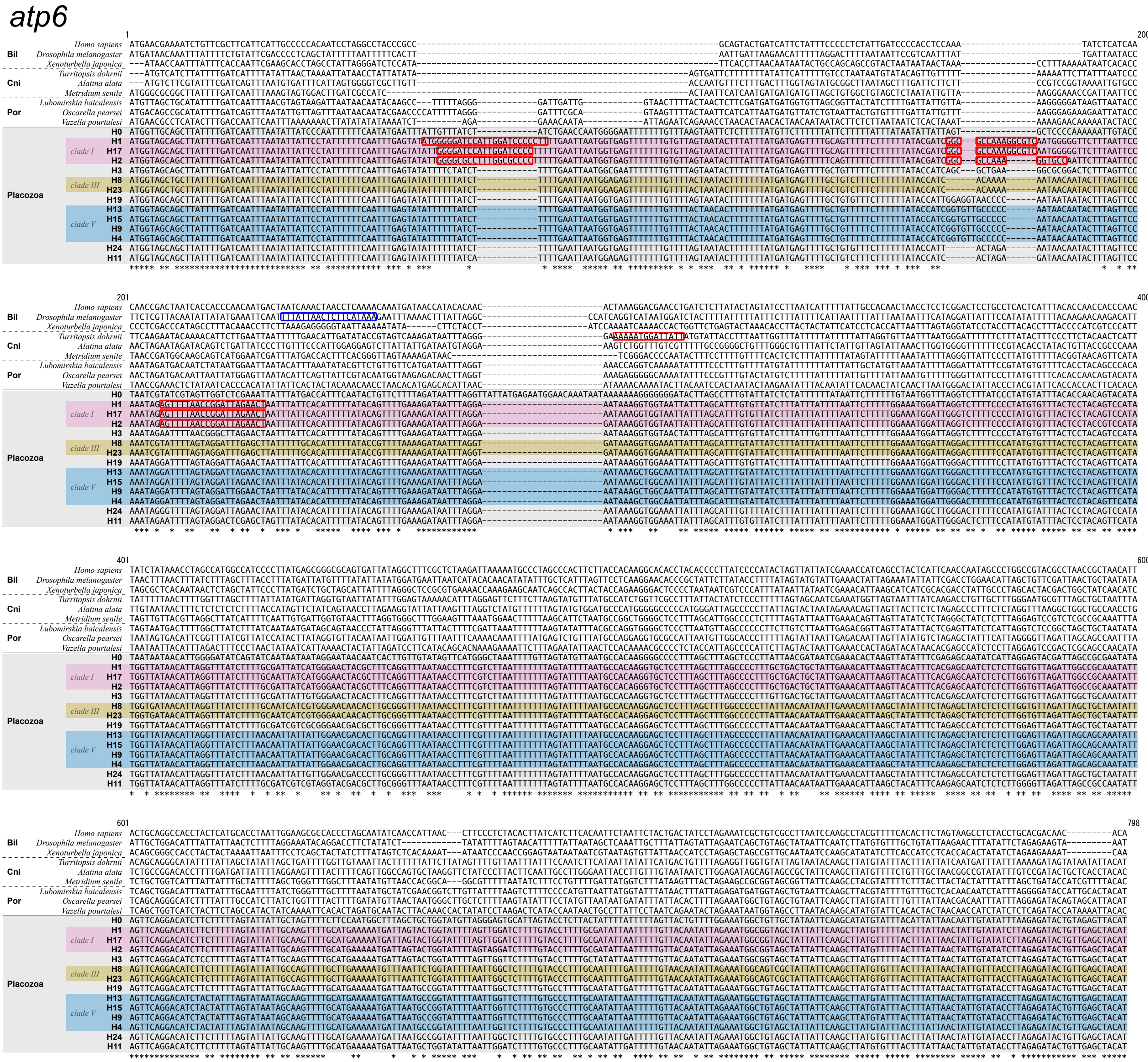
atp6
1
200
201
400
401
600
601
798
Bil
Homo sapiens
Drosophila melanogaster
Xenoturbella japonica
Cni
Turritopsis dohrnii
Alatina alata
Metridium senile
Por
Lubomirskia baicalensis
Oscarella pearsei
Vazella pourtalesi
Placozoa
clade I
clade III
clade V
H0
H1
H17
H2
H3
H8
H23
H19
H13
H15
H9
H4
H24
H11

cox1

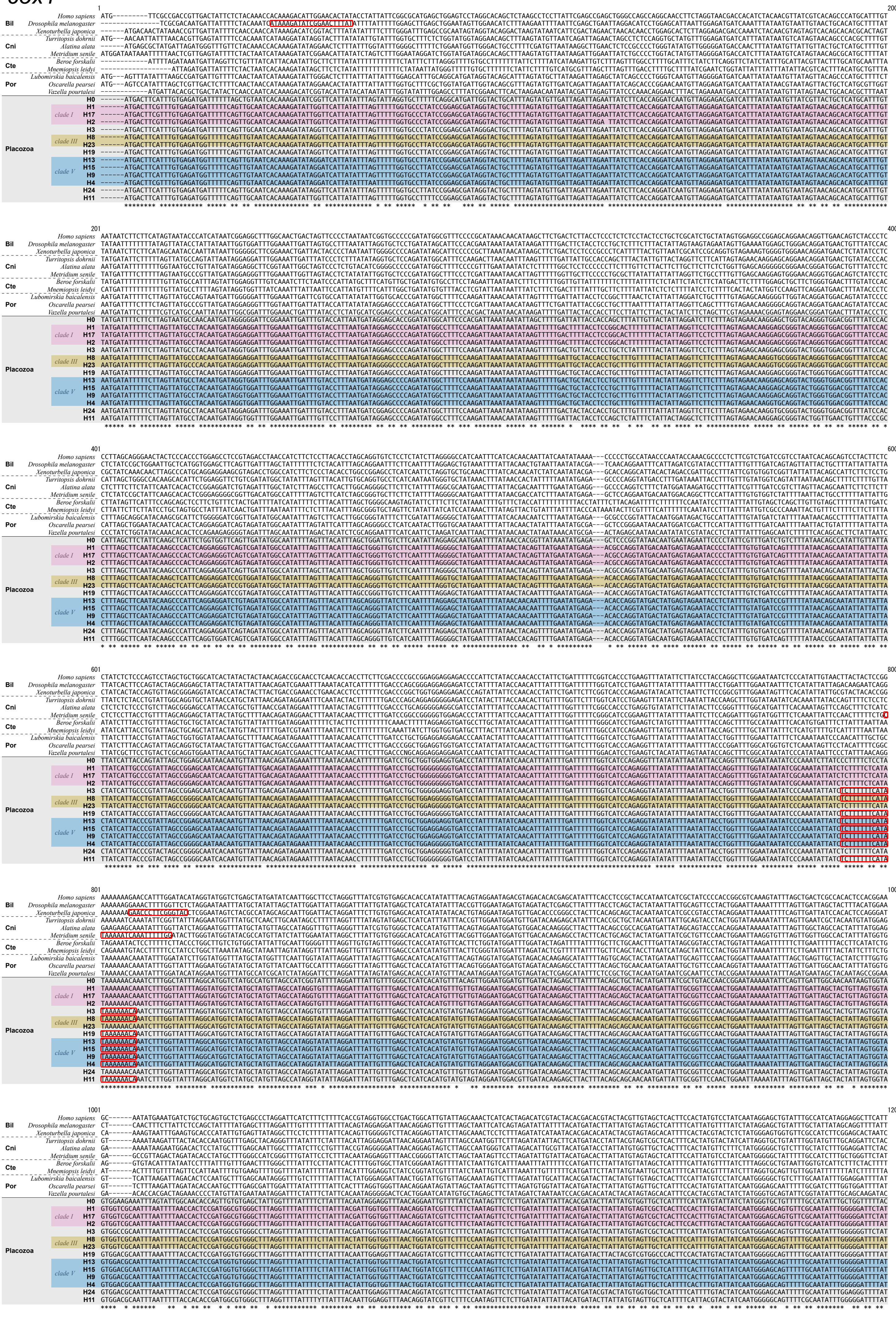
Homo sapiens
Bil
Drosophila melanogaster
Xenoturbella japonica
Turritopsis dohrnii
Cni
Alatina alata
Metridium senile
Beroe forskalii
Cte
Mnemiopsis leidyi
Lubomirskia baicalensis
Por
Oscarella pearsei
Vazella pourtalesi
Placozoa
clade I
clade III
clade V
H0
H1
H17
H2
H3
H8
H23
H19
H13
H15
H9
H4
H24
H11

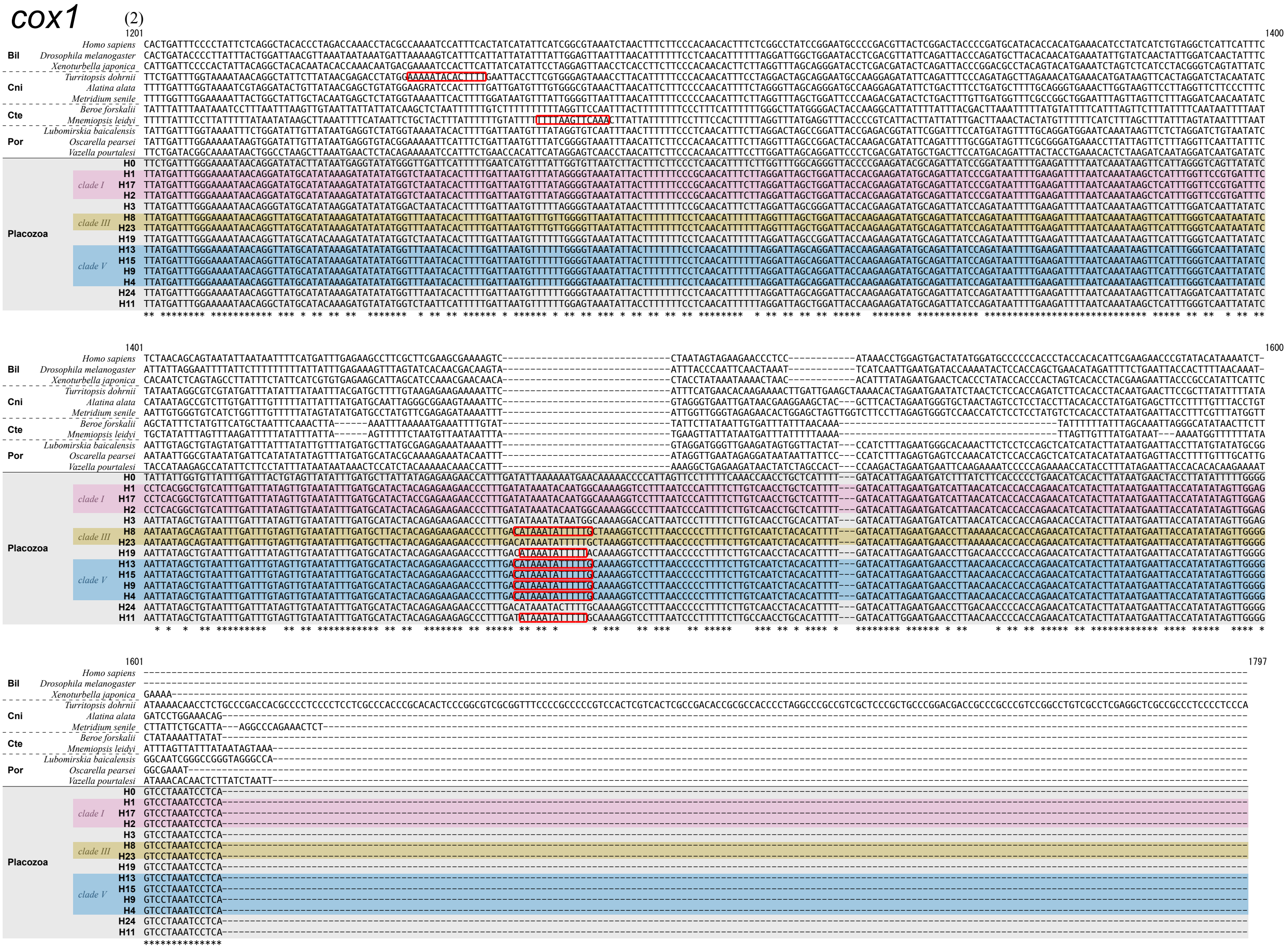
cox1
(2)
1201
1400
1401
1600
1601
1797
Bil
Cni
Cte
Por
Placozoa
Homo sapiens
Drosophila melanogaster
Xenoturbella japonica
Turritopsis dohrnii
Alatina alata
Metridium senile
Beroe forskalii
Mnemiopsis leidyi
Lubomirskia baicalensis
Oscarella pearsei
Vazella pourtalesi
H0
H1
H17
H2
H3
H8
H23
H19
H13
H15
H9
H4
H24
H11
clade I
clade III
clade V

cox2

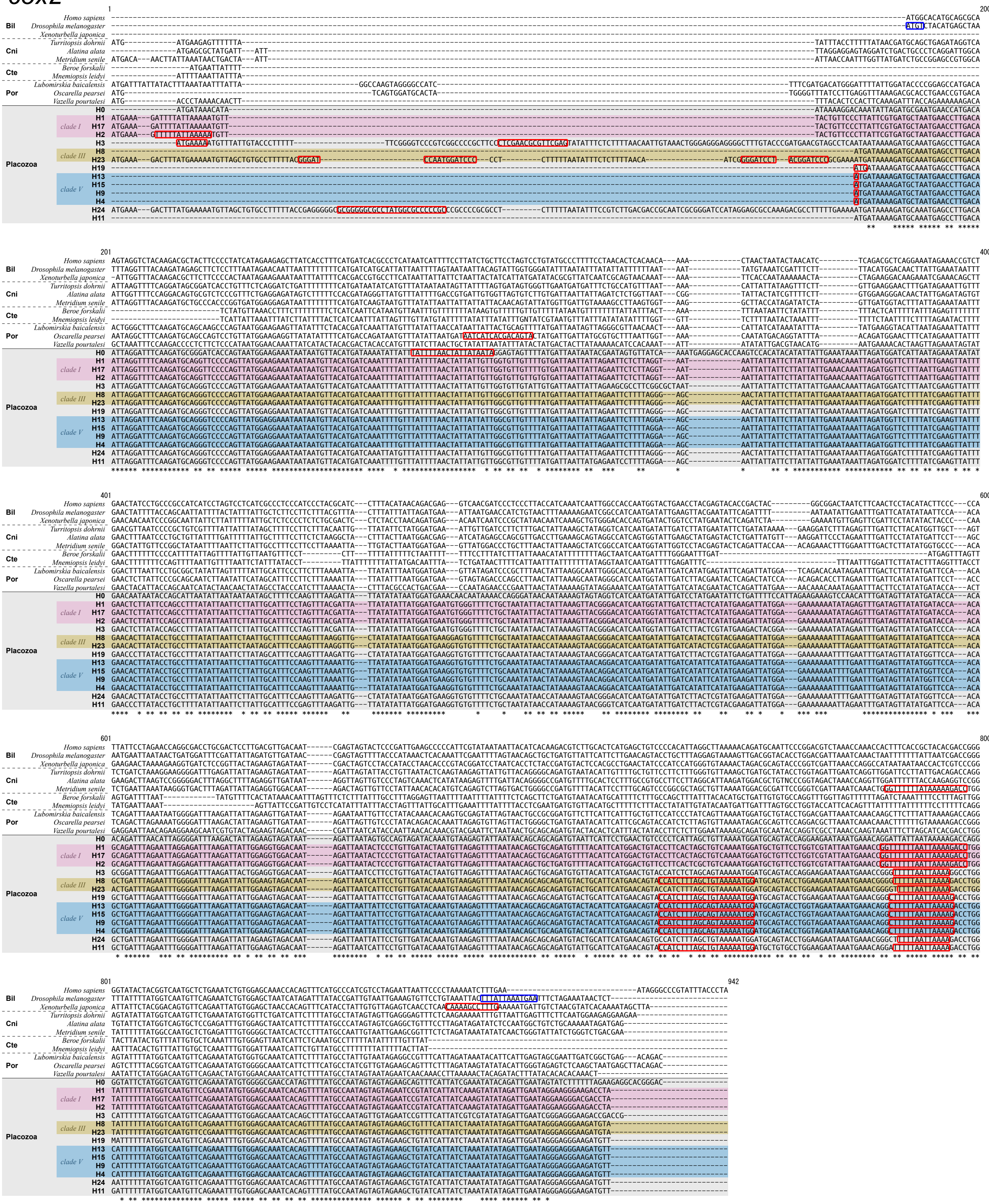

Bil
Homo sapiens
Drosophila melanogaster
Xenoturbella japonica
Cni
Turritopsis dohrnii
Alatina alata
Metridium senile
Cte
Beroe forskalii
Mnemiopsis leidyi
Por
Lubomirskia baicalensis
Oscarella pearsei
Vazella pourtalesi
Placozoa
H0
clade I
H1
H17
H2
H3
clade III
H8
H23
H19
clade V
H13
H15
H9
H4
H24
H11
1
200
201
400
401
600
601
800
801
942

cox3

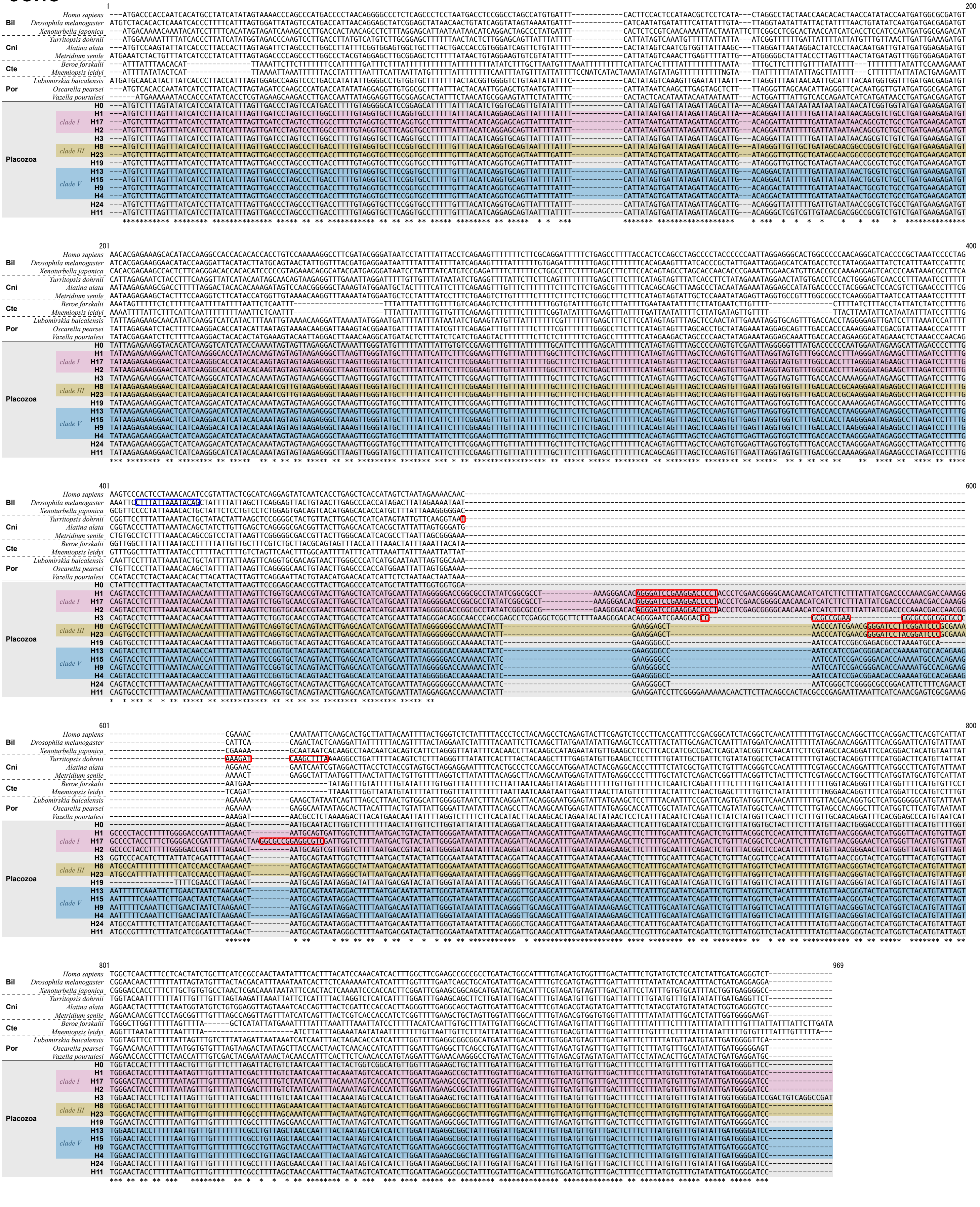

Homo sapiens
Bil
Drosophila melanogaster
Xenoturbella japonica
Turritopsis dohrnii
Cni
Alatina alata
Metridium senile
Beroe forskalii
Cte
Mnemiopsis leidyi
Lubomirskia baicalensis
Por
Oscarella pearsei
Vazella pourtalesi
Placozoa
clade I
clade III
clade V
H0
H1
H17
H2
H3
H8
H23
H19
H13
H15
H9
H4
H24
H11
1
200
201
400
401
600
601
800
801
969

nad1

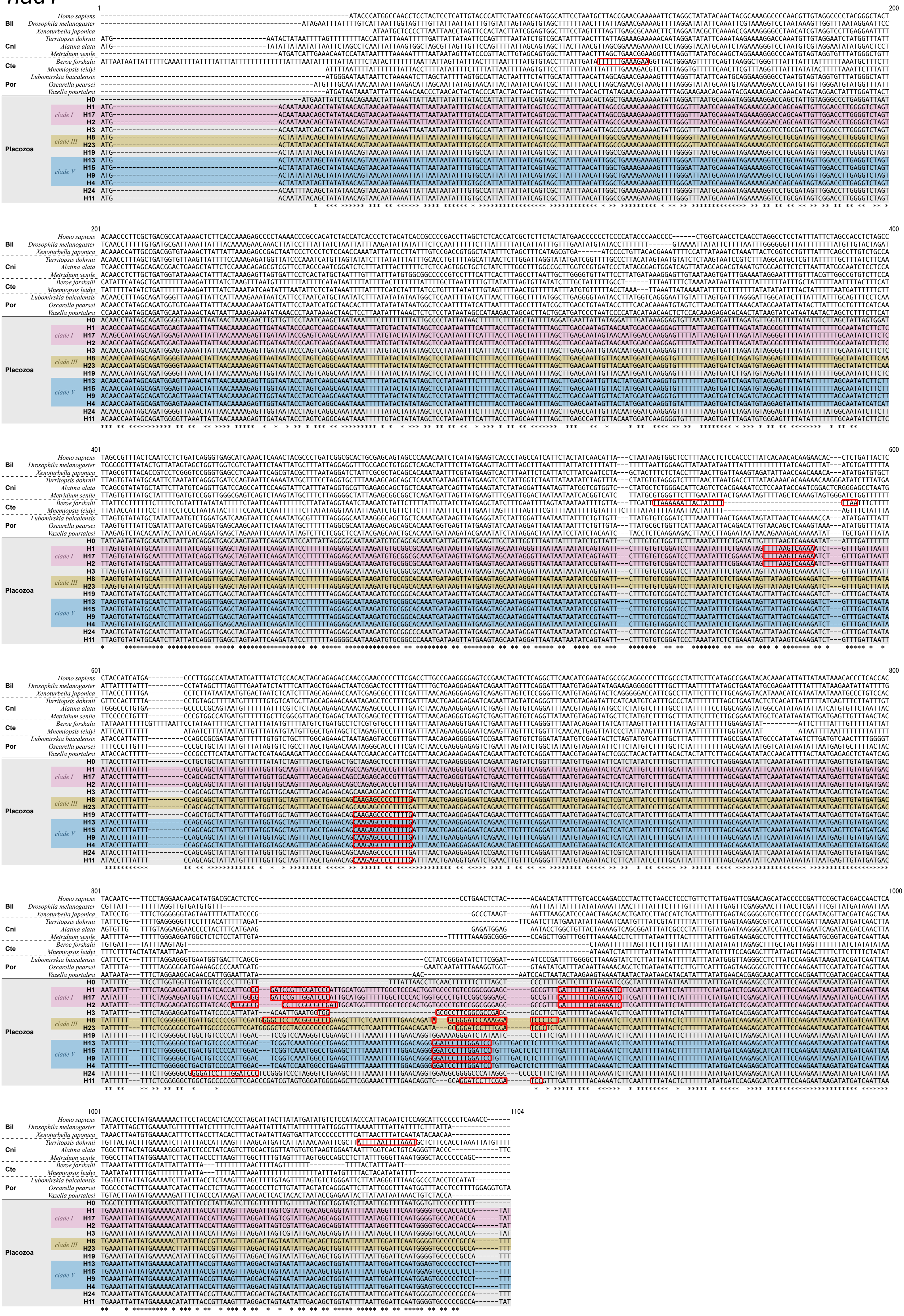
Bil
Homo sapiens
Drosophila melanogaster
Xenoturbella japonica
Cni
Turritopsis dohrnii
Alatina alata
Metridium senile
Cte
Beroe forskalii
Mnemiopsis leidyi
Por
Lubomirskia baicalensis
Oscarella pearsei
Vazella pourtalesi
Placozoa
H0
H1
H17
H2
H3
H8
H23
H19
H13
H15
H9
H4
H24
H11
clade I
clade III
clade V

nad2

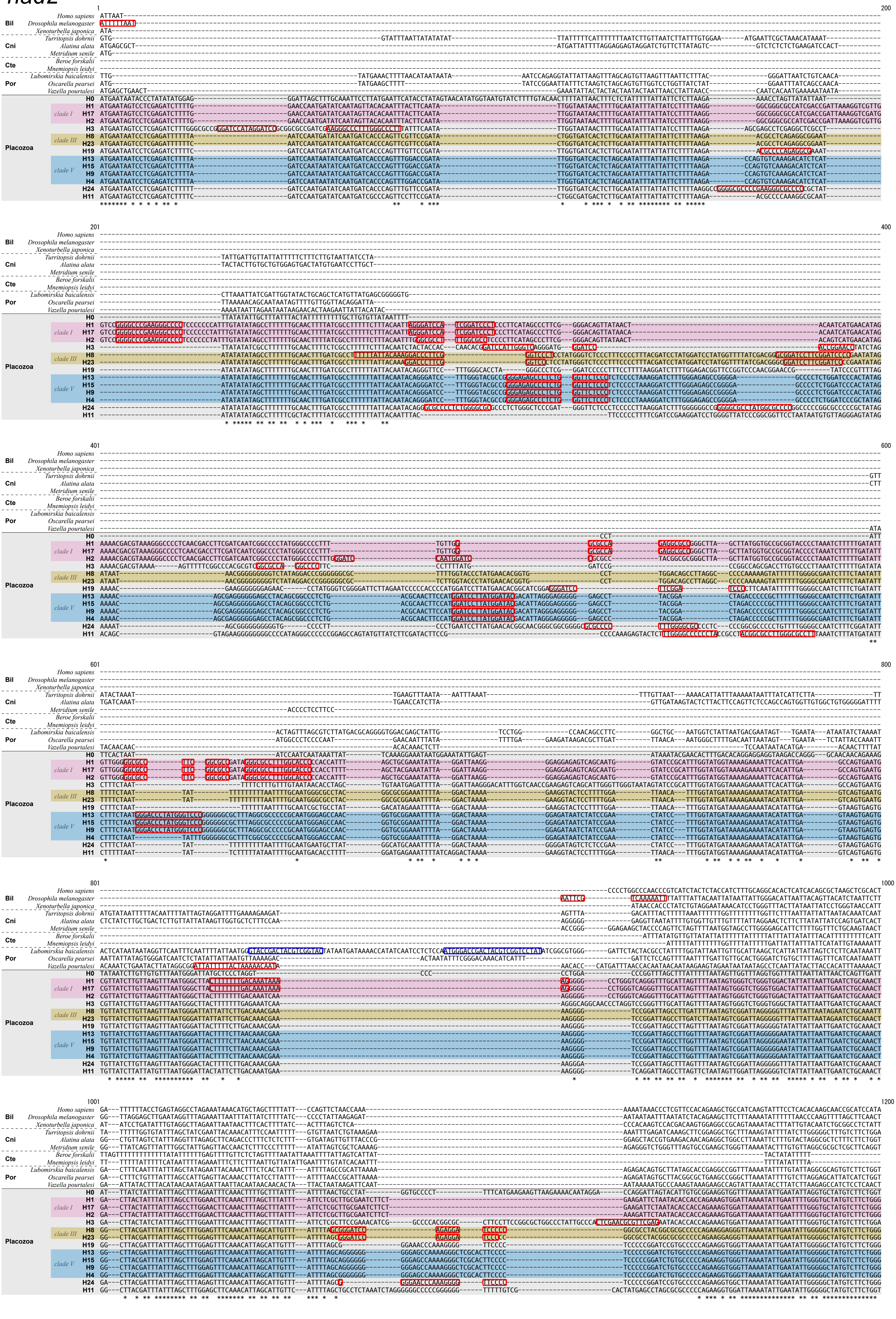
Bil
Cni
Cte
Por
Placozoa
clade I
clade III
clade V
Homo sapiens
Drosophila melanogaster
Xenoturbella japonica
Turritopsis dohrnii
Alatina alata
Metridium senile
Beroe forskalii
Mnemiopsis leidyi
Lubomirskia baicalensis
Oscarella pearsei
Vazella pourtalesi

nad2 (2)

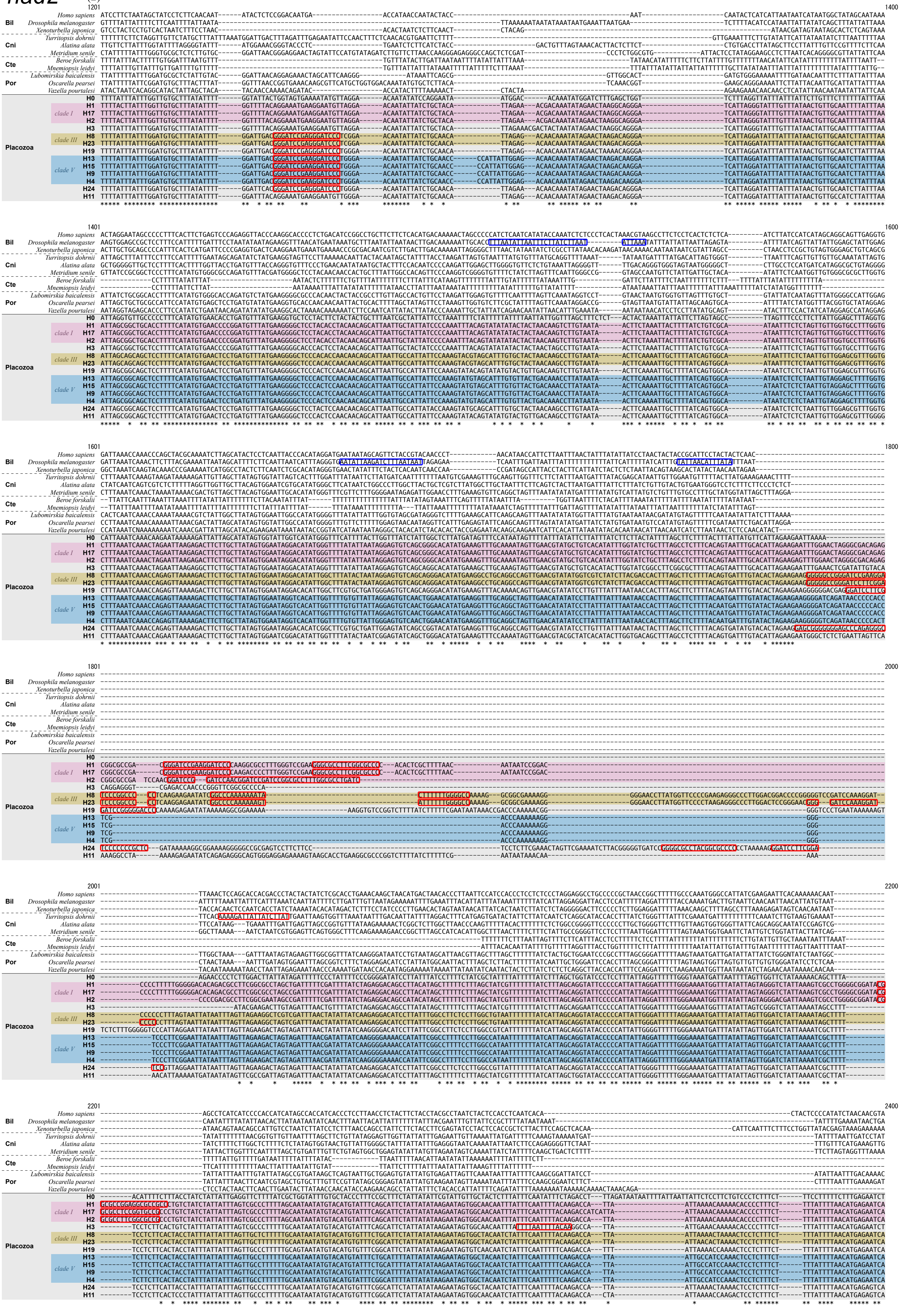

Homo sapiens
Drosophila melanogaster
Xenoturbella japonica
Turritopsis dohrnii
Alatina alata
Metridium senile
Beroe forskalii
Mnemiopsis leidyi
Lubomirskia baicalensis
Oscarella pearsei
Vazella pourtalesi
Bil
Cni
Cte
Por
Placozoa
clade I
clade III
clade V

nad2 (3)

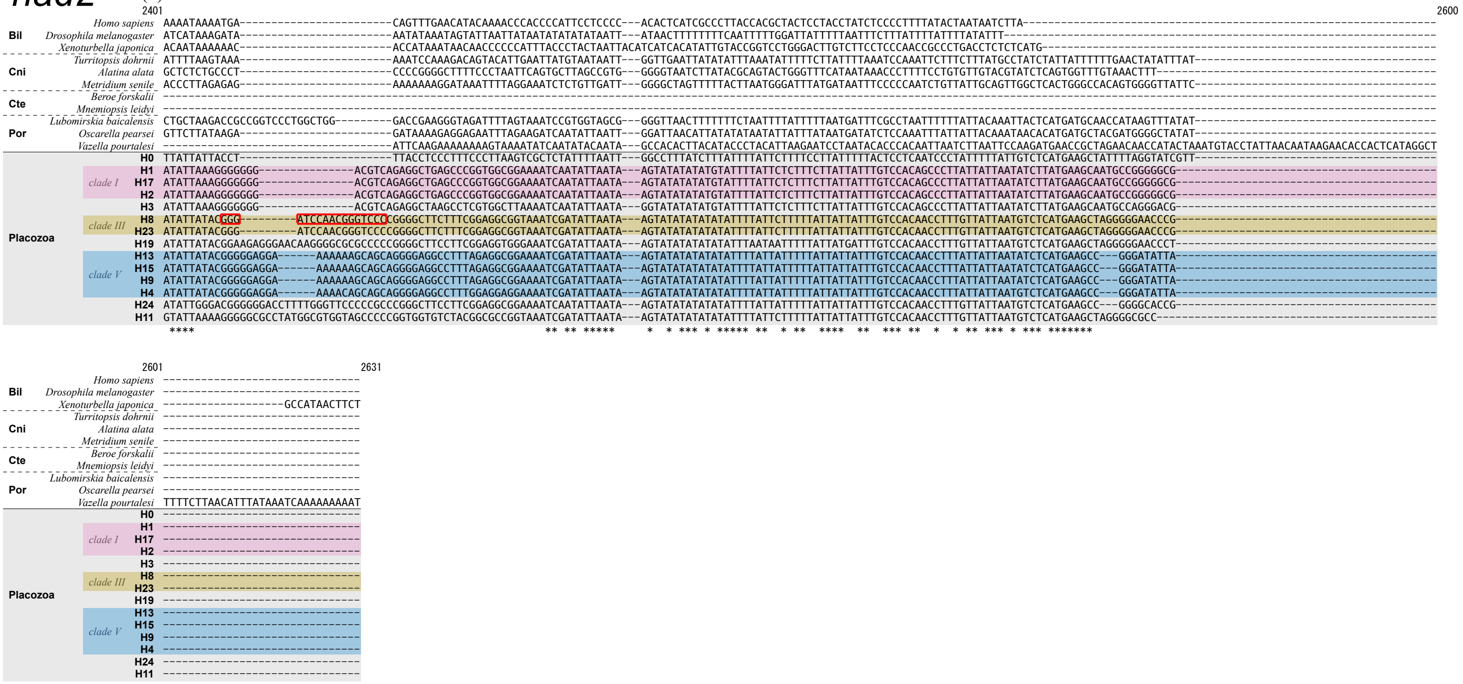
2401
2600
2601
2631
Homo sapiens
Drosophila melanogaster
Xenoturbella japonica
Turritopsis dohrnii
Alatina alata
Metridium senile
Beroe forskalii
Mnemiopsis leidyi
Lubomirskia baicalensis
Oscarella pearsei
Vazella pourtalesi
Bil
Cni
Cte
Por
Placozoa
clade I
clade III
clade V
H0
H1
H17
H2
H3
H8
H23
H19
H13
H15
H9
H4
H24
H11

nad3

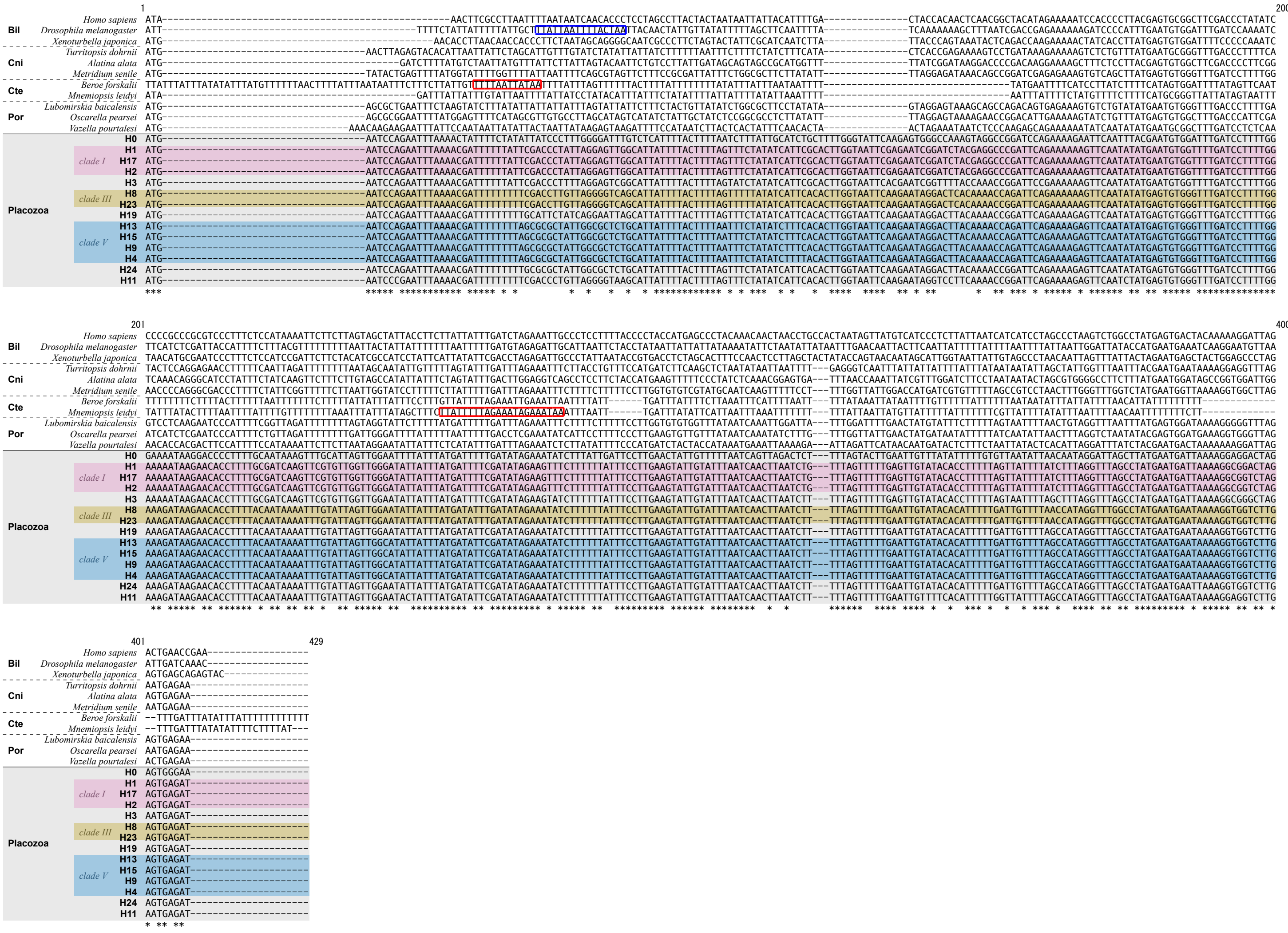
1
200
201
400
401
429
Homo sapiens
Drosophila melanogaster
Xenoturbella japonica
Turritopsis dohrnii
Alatina alata
Metridium senile
Beroe forskalii
Mnemiopsis leidyi
Lubomirskia baicalensis
Oscarella pearsei
Vazella pourtalesi
Bil
Cni
Cte
Por
Placozoa
clade I
clade III
clade V
H0
H1
H17
H2
H3
H8
H23
H19
H13
H15
H9
H4
H24
H11

nad4

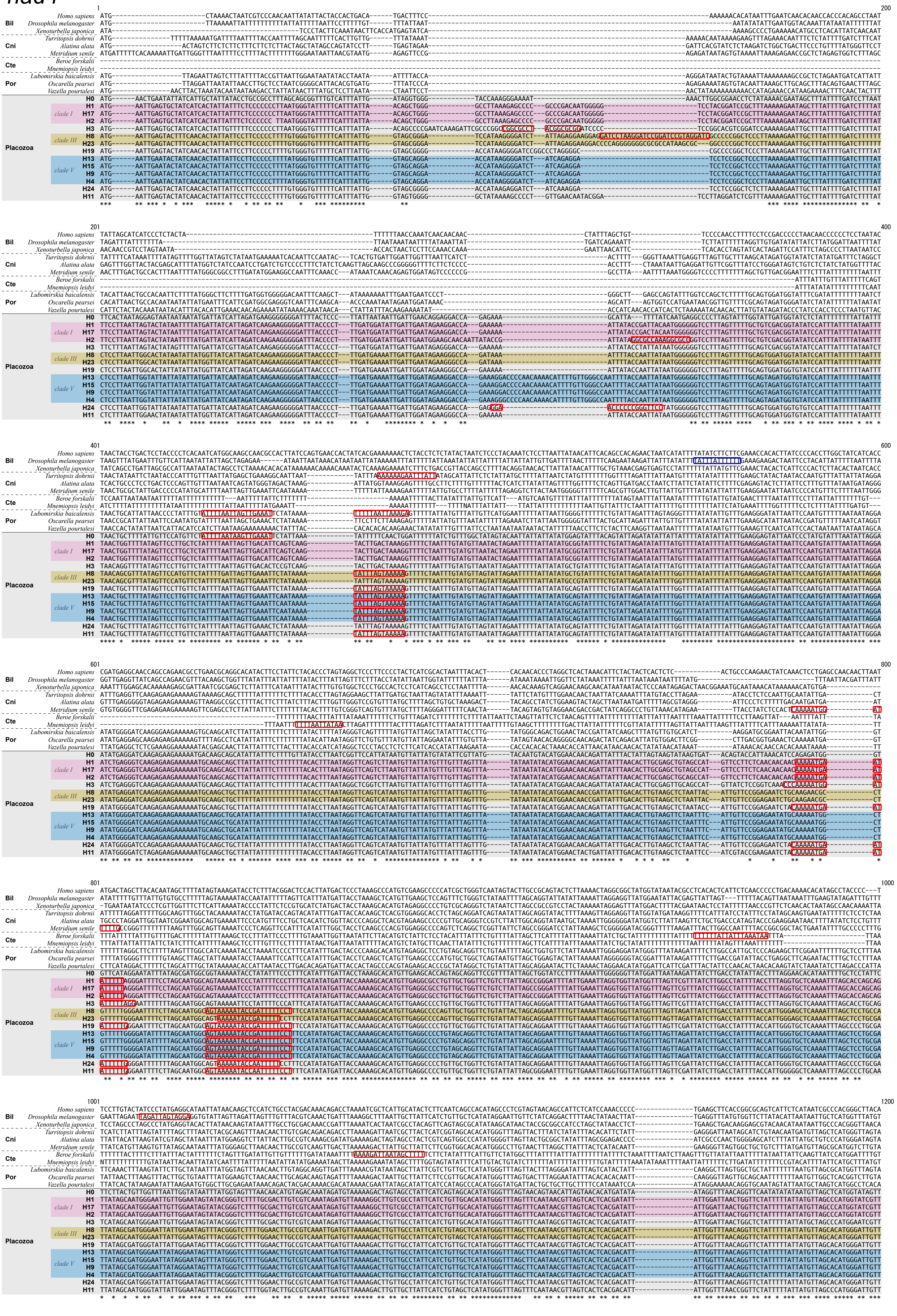

Bil
Homo sapiens
Drosophila melanogaster
Xenoturbella japonica
Cni
Turritopsis dohrnii
Alatina alata
Metridium senile
Cte
Beroe forskalii
Mnemiopsis leidyi
Por
Lubomirskia baicalensis
Oscarella pearsei
Vazella pourtalesi
Placozoa
clade I
clade III
clade V
H0
H1
H17
H2
H3
H8
H23
H19
H13
H15
H9
H4
H24
H11
1
200
201
400
401
600
601
800
801
1000
1001
1200

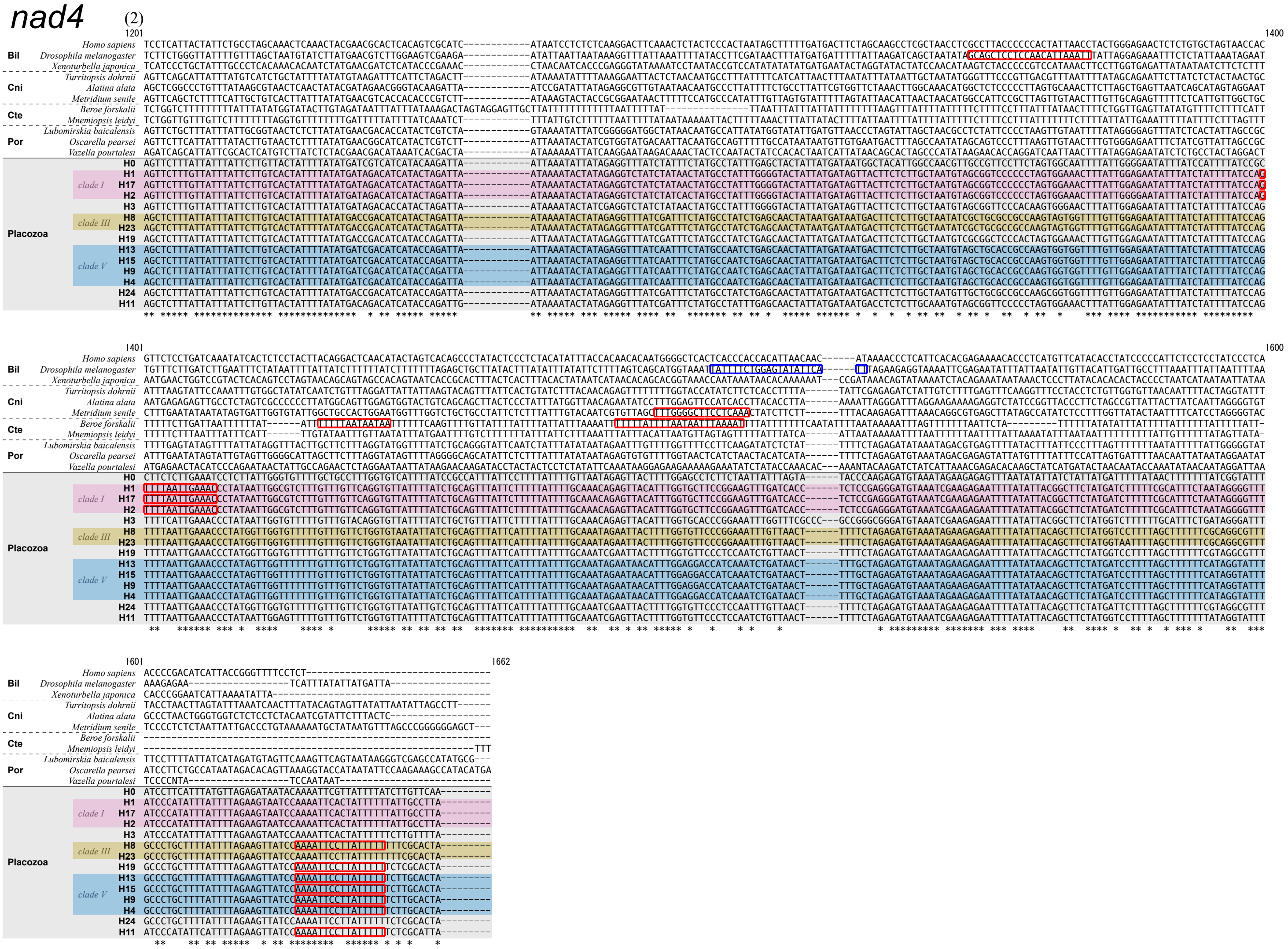
nad4 (2)
1201
1400
1401
1600
1601
1662
Homo sapiens
Drosophila melanogaster
Xenoturbella japonica
Turritopsis dohrnii
Alatina alata
Metridium senile
Beroe forskalii
Mnemiopsis leidyi
Lubomirskia baicalensis
Oscarella pearsei
Vazella pourtalesi
Bil
Cni
Cte
Por
Placozoa
clade I
clade III
clade V
H0
H1
H17
H2
H3
H8
H23
H19
H13
H15
H9
H4
H24
H11

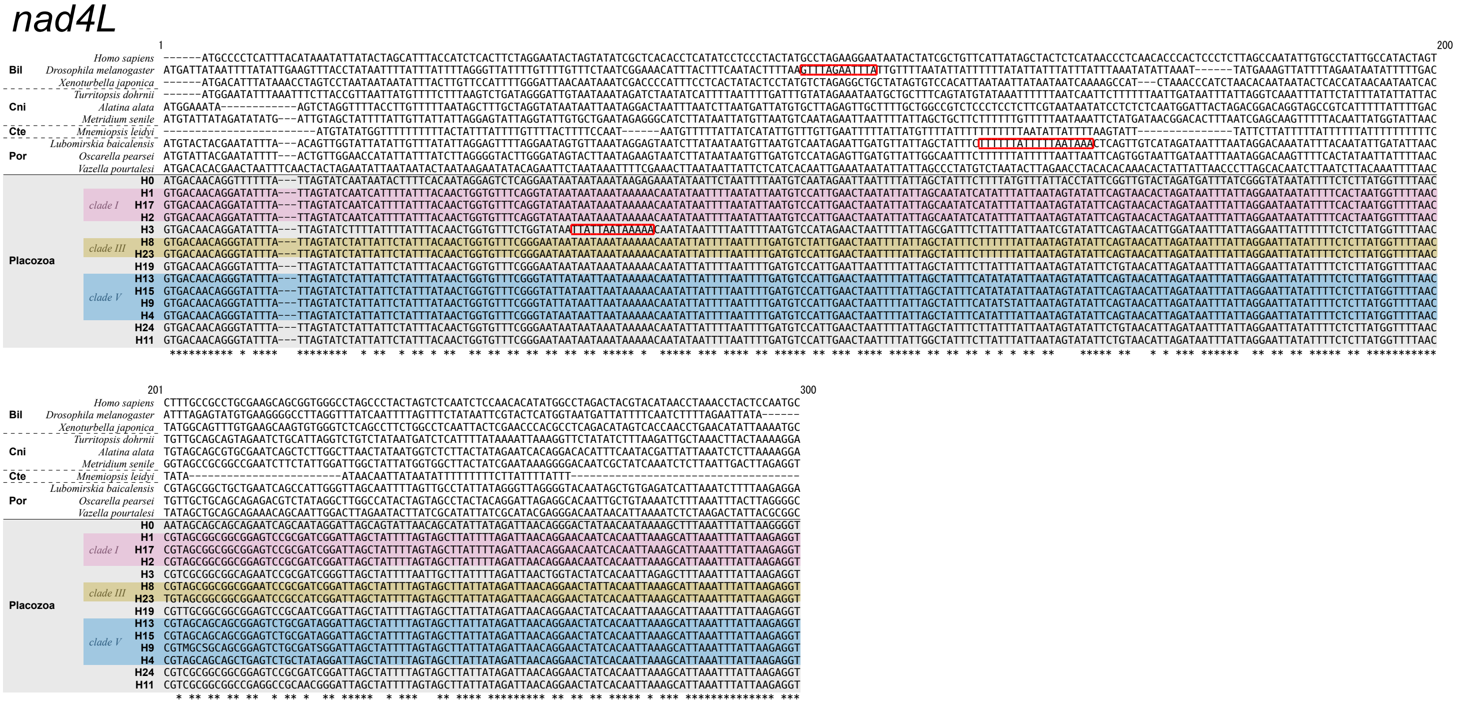
nad4L
1
200
201
300
Homo sapiens
Drosophila melanogaster
Xenoturbella japonica
Turritopsis dohrnii
Alatina alata
Metridium senile
Mnemiopsis leidyi
Lubomirskia baicalensis
Oscarella pearsei
Vazella pourtalesi
Bil
Cni
Cte
Por
Placozoa
clade I
clade III
clade V
H0
H1
H17
H2
H3
H8
H23
H19
H13
H15
H9
H4
H24
H11

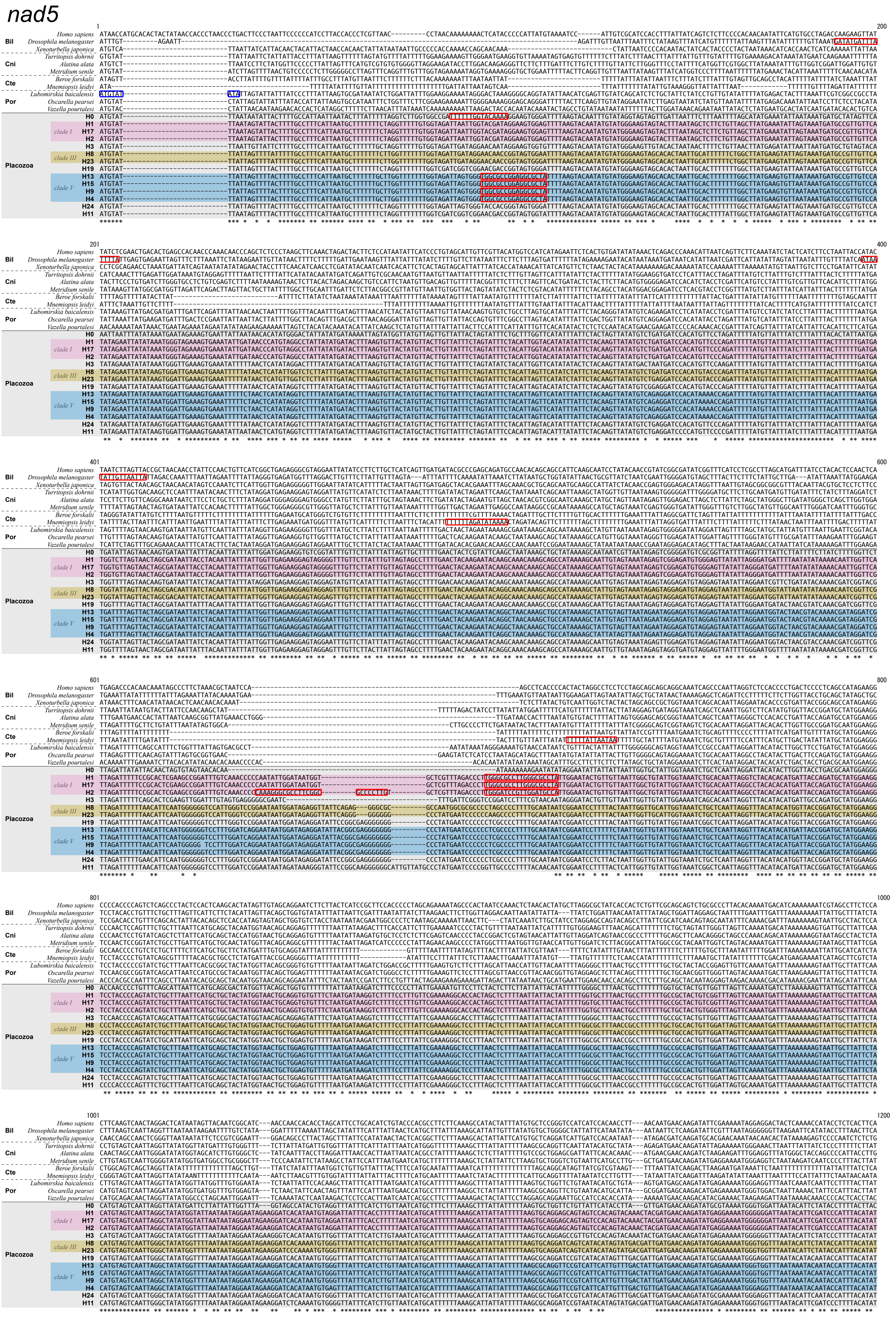
nad5
Homo sapiens
Bil
Drosophila melanogaster
Xenoturbella japonica
Turritopsis dohrnii
Cni
Alatina alata
Metridium senile
Beroe forskalii
Cte
Mnemiopsis leidyi
Lubomirskia baicalensis
Por
Oscarella pearsei
Vazella pourtalesi
Placozoa
clade I
clade III
clade V
H0
H1
H17
H2
H3
H8
H23
H19
H13
H15
H9
H4
H24
H11
1
200
201
400
401
600
601
800
801
1000
1001
1200

nad5 (2)

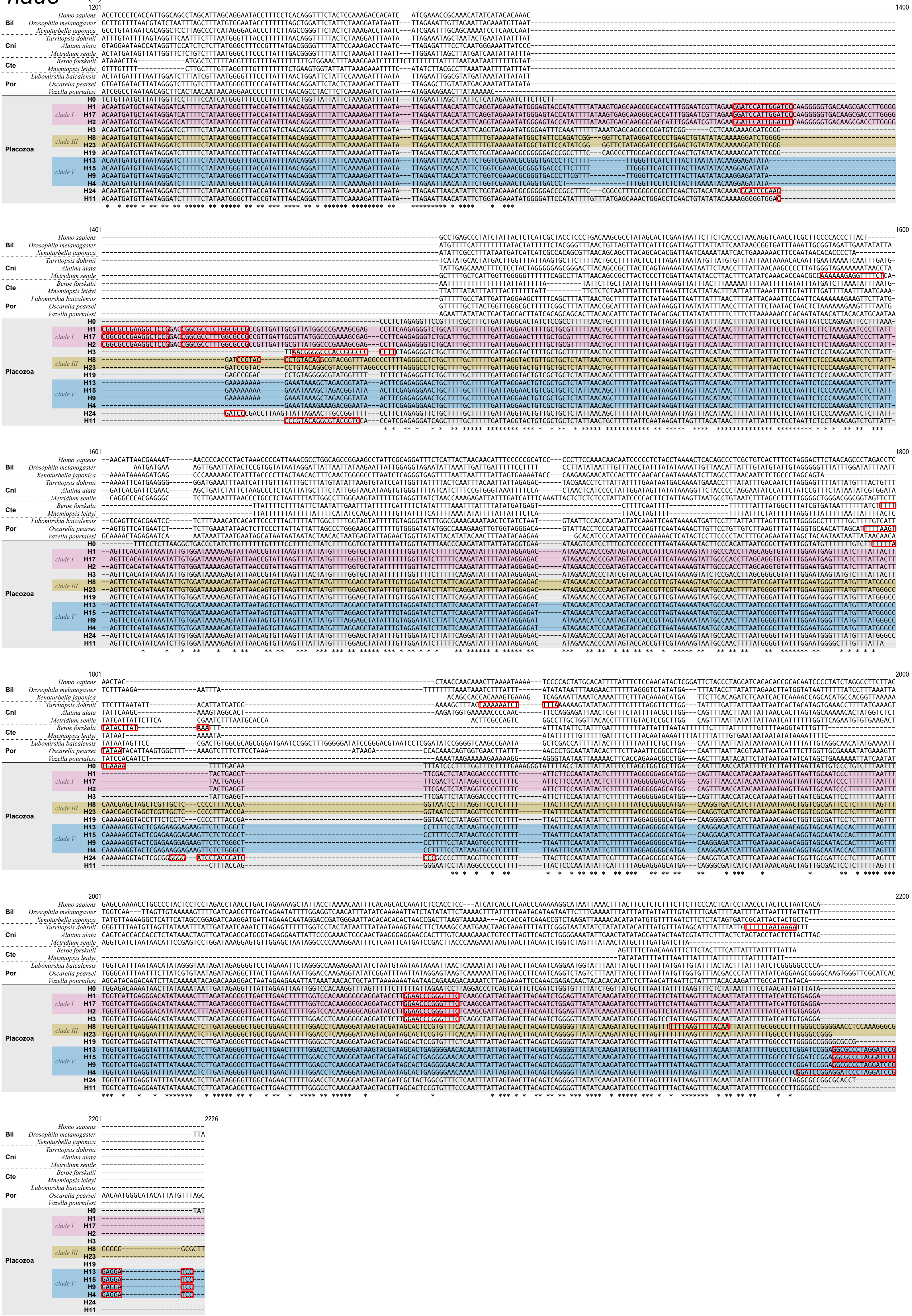
1201
1400
1401
1600
1601
1800
1801
2000
2001
2200
2201
2226
Homo sapiens
Drosophila melanogaster
Xenoturbella japonica
Turritopsis dohrnii
Alatina alata
Metridium senile
Beroe forskalii
Mnemiopsis leidyi
Lubomirskia baicalensis
Oscarella pearsei
Vazella pourtalesi
Bil
Cni
Cte
Por
Placozoa
clade I
clade III
clade V
H0
H1
H17
H2
H3
H8
H23
H19
H13
H15
H9
H4
H24
H11

nad6

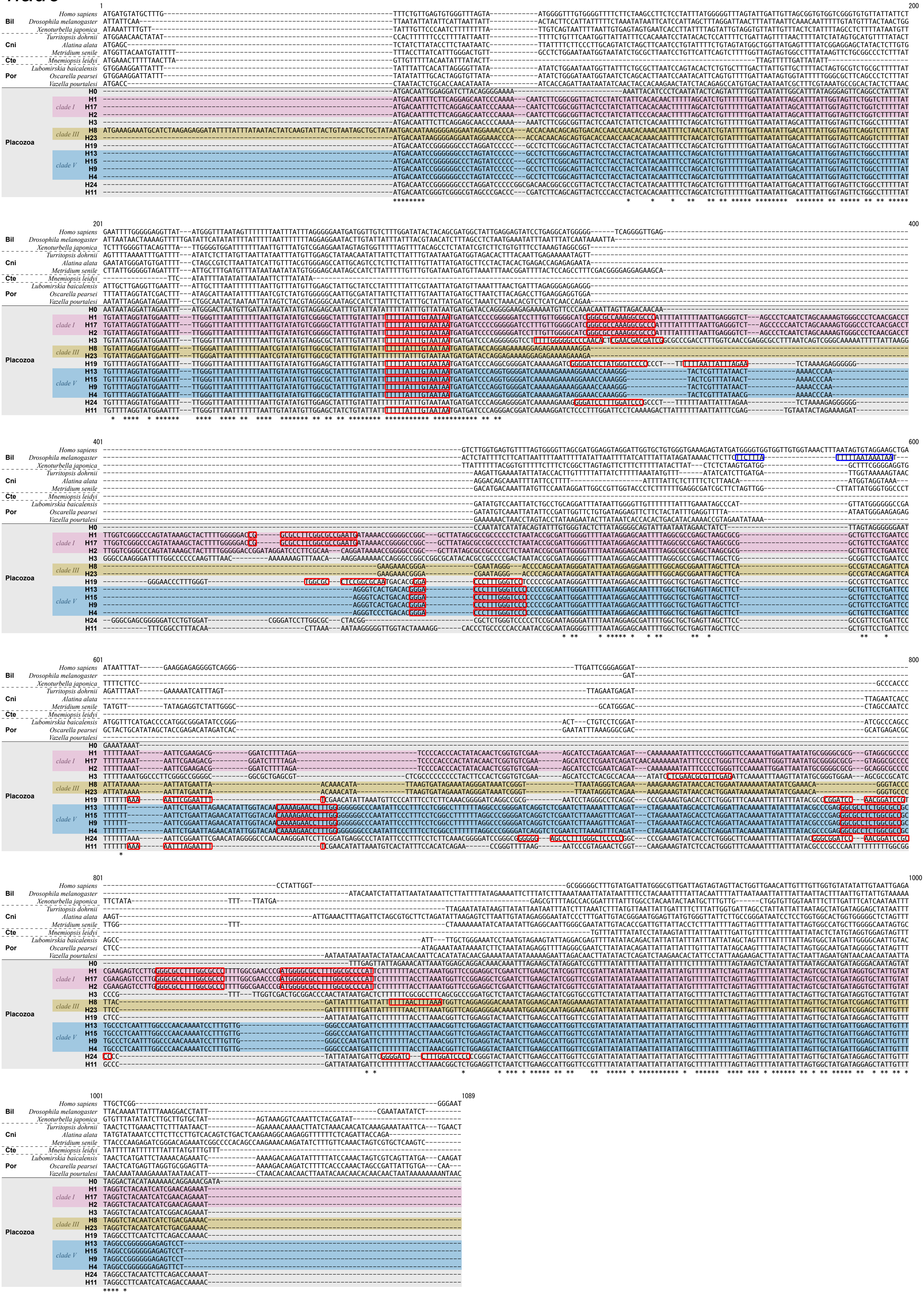

Homo sapiens
Drosophila melanogaster
Xenoturbella japonica
Turritopsis dohrnii
Alatina alata
Metridium senile
Mnemiopsis leidyi
Lubomirskia baicalensis
Oscarella pearsei
Vazella pourtalesi
Bil
Cni
Cte
Por
Placozoa
clade I
clade III
clade V
H0
H1
H17
H2
H3
H8
H23
H19
H13
H15
H9
H4
H24
H11

16S

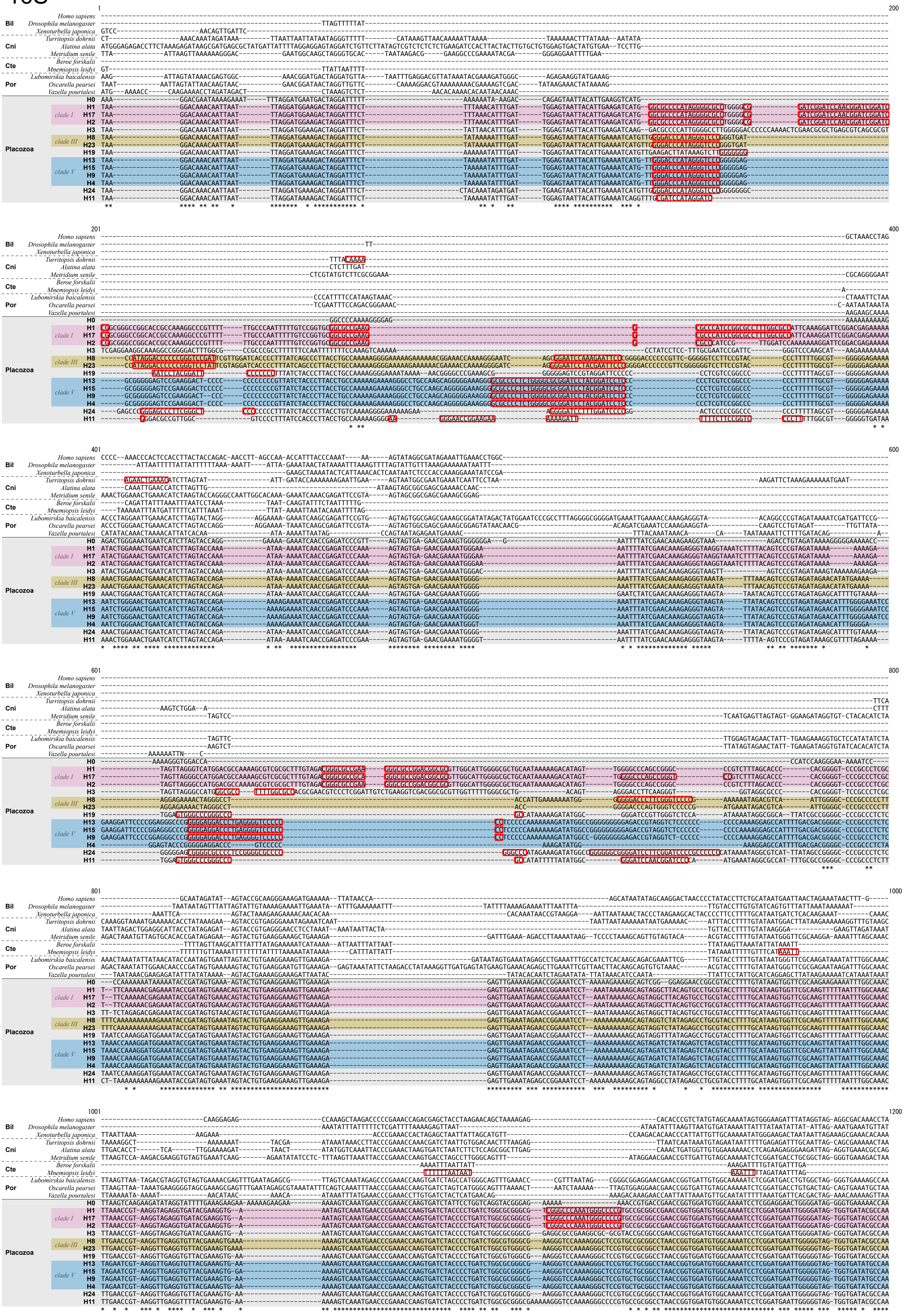
Homo sapiens
Drosophila melanogaster
Xenoturbella japonica
Turritopsis dohrnii
Alatina alata
Metridium senile
Beroe forskalii
Mnemiopsis leidyi
Lubomirskia baicalensis
Oscarella pearsei
Vazella pourtalesi
Bil
Cni
Cte
Por
Placozoa
clade I
clade III
clade V
H0
H1
H17
H2
H3
H8
H23
H19
H13
H15
H9
H4
H24
H11

16S

(2)

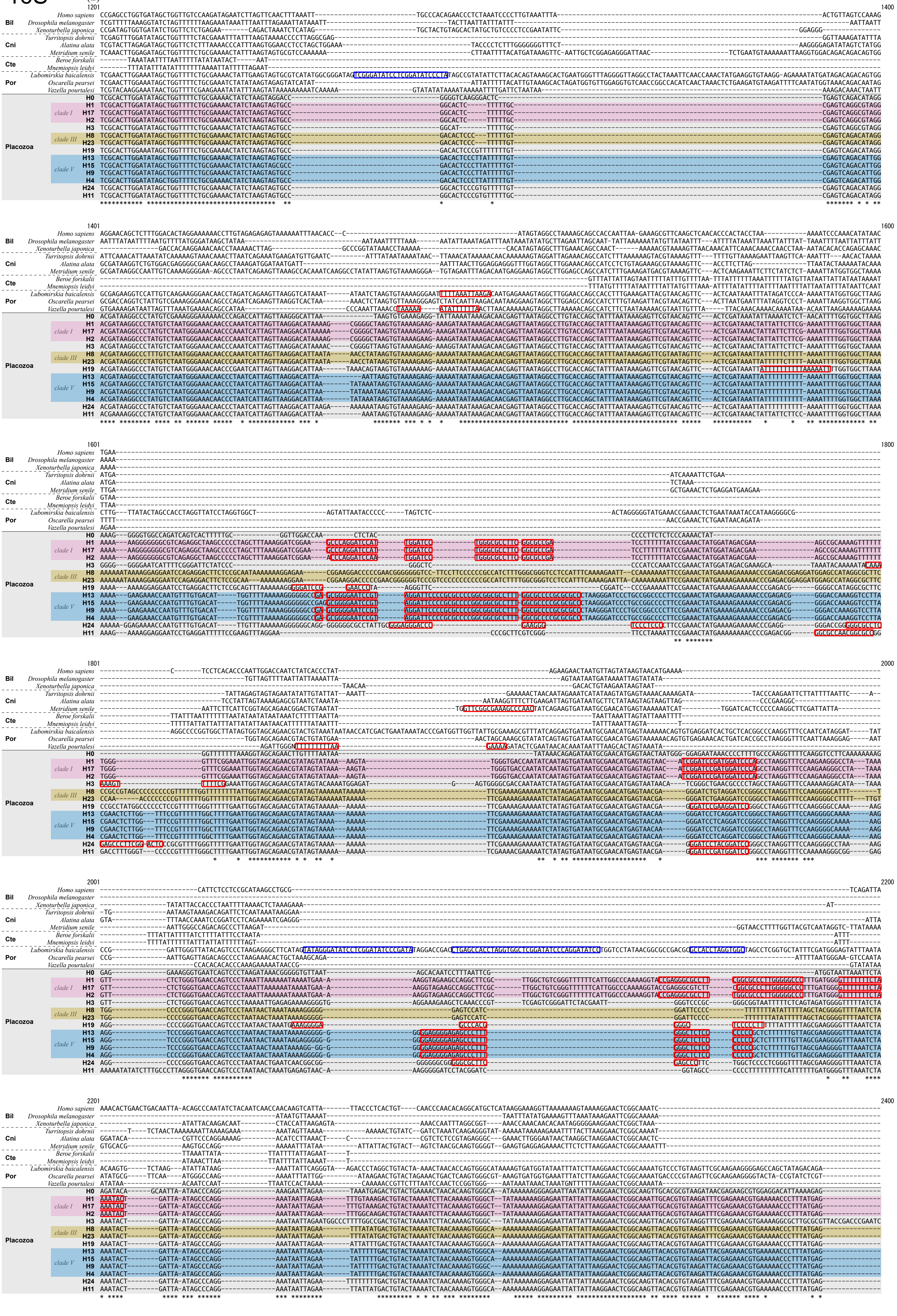

Homo sapiens
Drosophila melanogaster
Xenoturbella japonica
Turritopsis dohrnii
Alatina alata
Metridium senile
Beroe forskalii
Mnemiopsis leidyi
Lubomirskia baicalensis
Oscarella pearsei
Vazella pourtalesi
Bil
Cni
Cte
Por
Placozoa
clade I
clade III
clade V
H0
H1
H17
H2
H3
H8
H23
H19
H13
H15
H9
H4
H24
H11
1201
1400
1401
1600
1601
1800
1801
2000
2001
2200
2201
2400

16S (3)

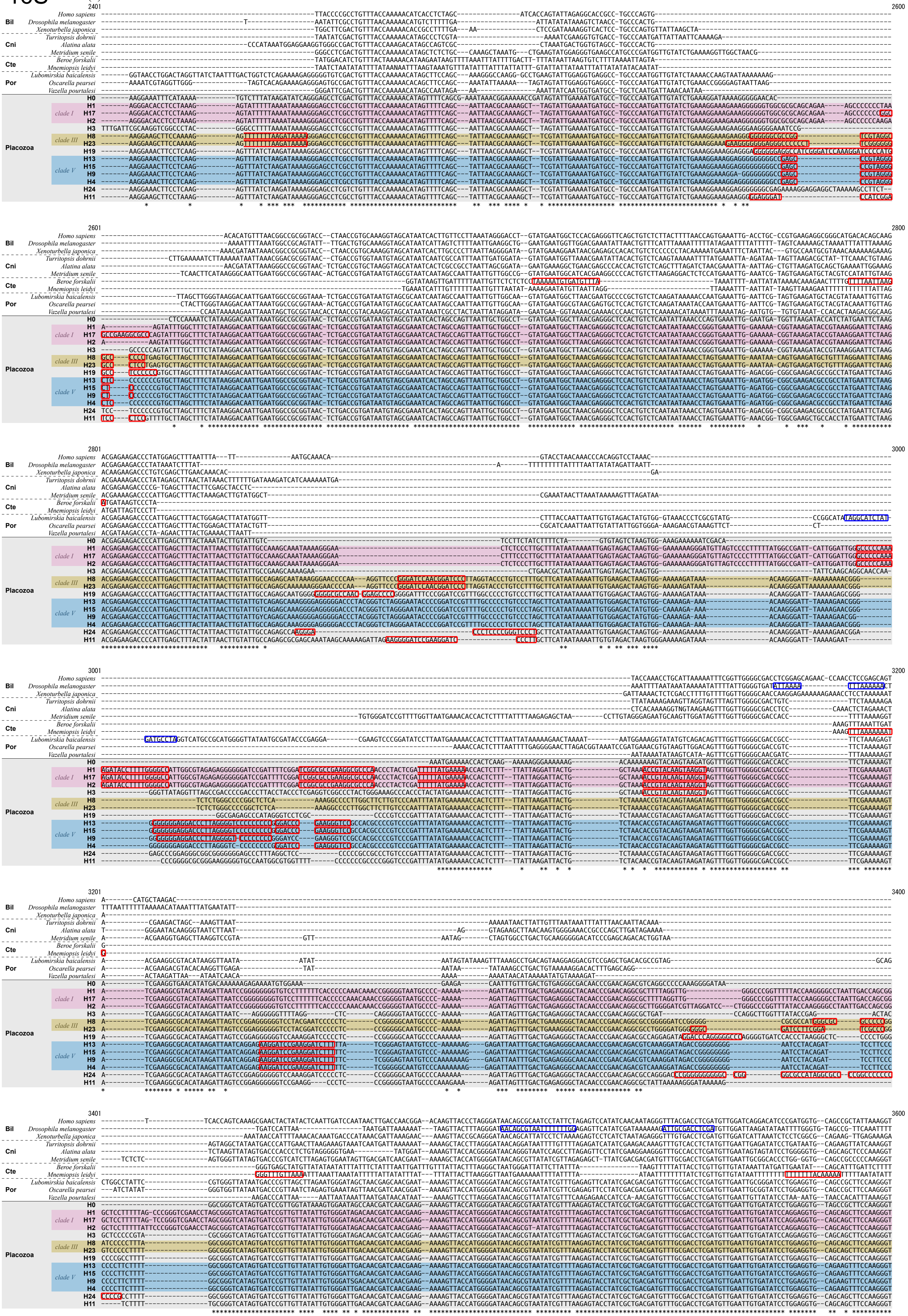
Homo sapiens
Drosophila melanogaster
Xenoturbella japonica
Turritopsis dohrnii
Alatina alata
Metridium senile
Beroe forskalii
Mnemiopsis leidyi
Lubomirskia baicalensis
Oscarella pearsei
Vazella pourtalesi
Bil
Cni
Cte
Por
Placozoa
clade I
clade III
clade V
H0
H1
H17
H2
H3
H8
H23
H19
H13
H15
H9
H4
H24
H11

16S

(4)

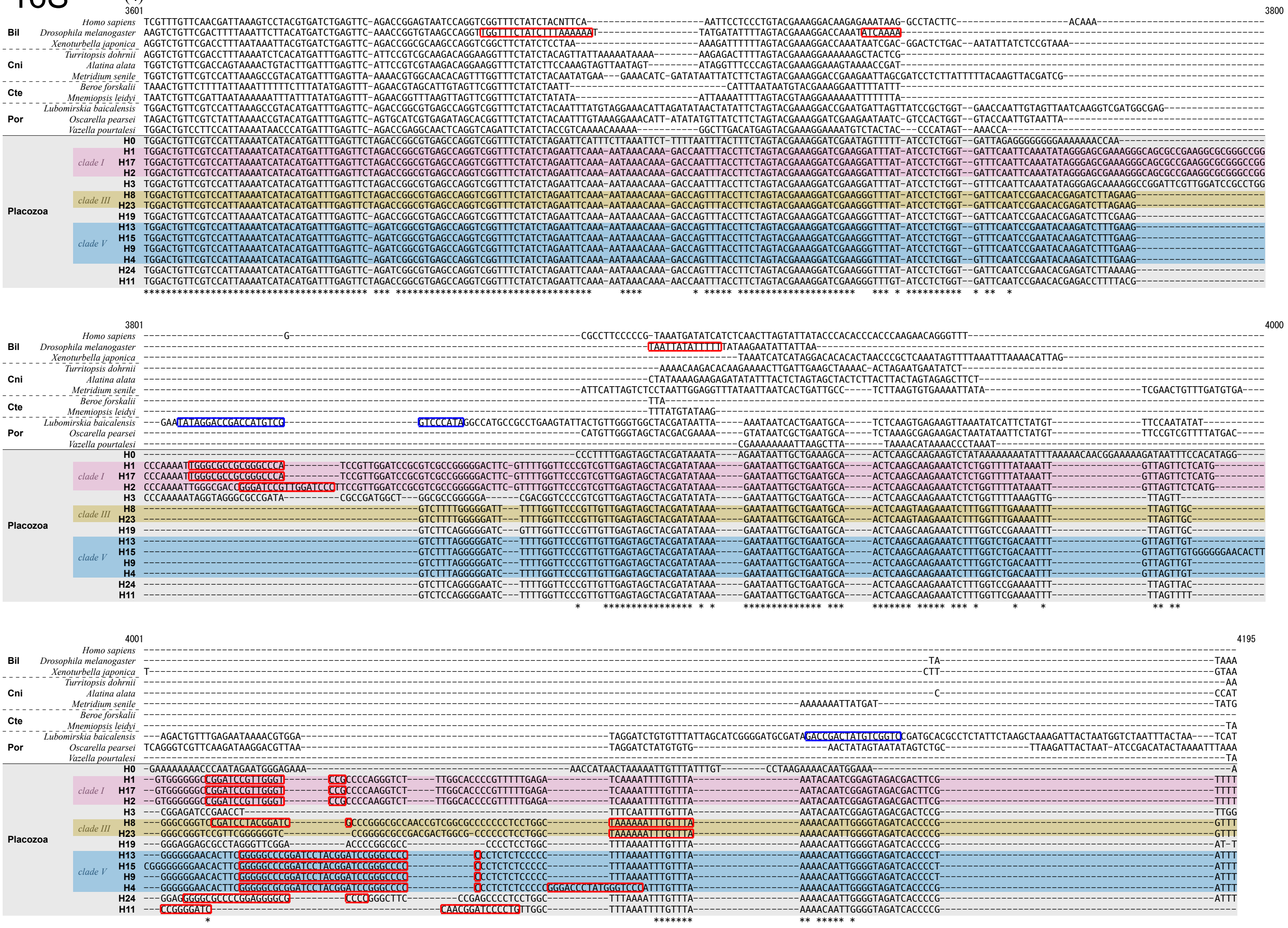

3601
3800
Homo sapiens
Bil
Drosophila melanogaster
Xenoturbella japonica
Turritopsis dohrnii
Cni
Alatina alata
Metridium senile
Beroe forskalii
Cte
Mnemiopsis leidyi
Lubomirskia baicalensis
Por
Oscarella pearsei
Vazella pourtalesi
H0
H1
clade I
H17
H2
H3
H8
clade III
H23
H19
Placozoa
H13
H15
clade V
H9
H4
H24
H11
3801
4000
4001
4195

12S

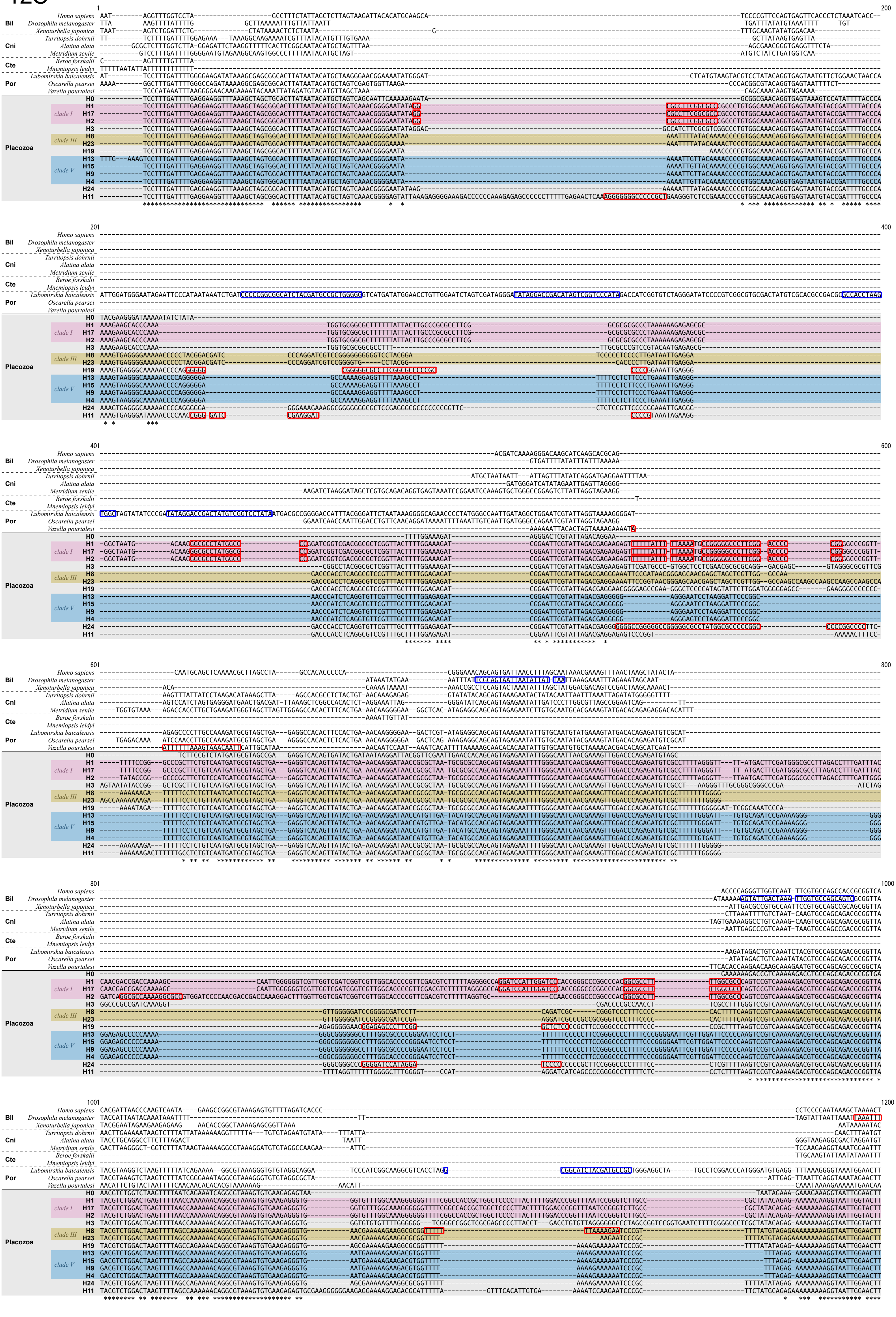
Homo sapiens
Drosophila melanogaster
Xenoturbella japonica
Turritopsis dohrnii
Alatina alata
Metridium senile
Beroe forskalii
Mnemiopsis leidyi
Lubomirskia baicalensis
Oscarella pearsei
Vazella pourtalesi
Bil
Cni
Cte
Por
Placozoa
clade I
clade III
clade V
H0
H1
H17
H2
H3
H8
H23
H19
H13
H15
H9
H4
H24
H11

12S (2)

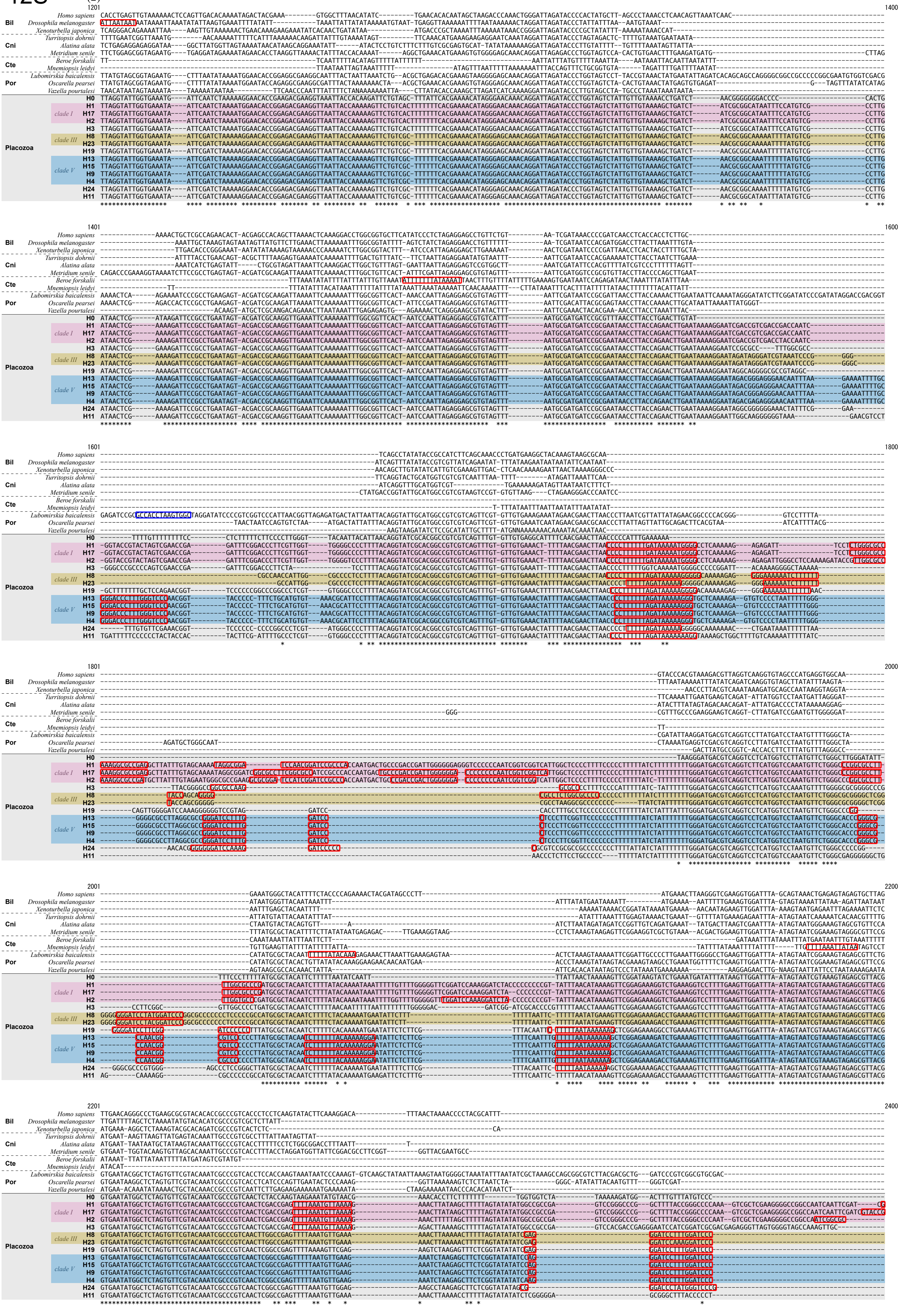
Homo sapiens
Bil
Drosophila melanogaster
Xenoturbella japonica
Turritopsis dohrnii
Cni
Alatina alata
Metridium senile
Beroe forskalii
Cte
Mnemiopsis leidyi
Lubomirskia baicalensis
Por
Oscarella pearsei
Vazella pourtalesi
Placozoa
clade I
clade III
clade V
H0
H1
H17
H2
H3
H8
H23
H19
H13
H15
H9
H4
H24
H11
1201
1400
1401
1600
1601
1800
1801
2000
2001
2200
2201
2400

12S

(3)

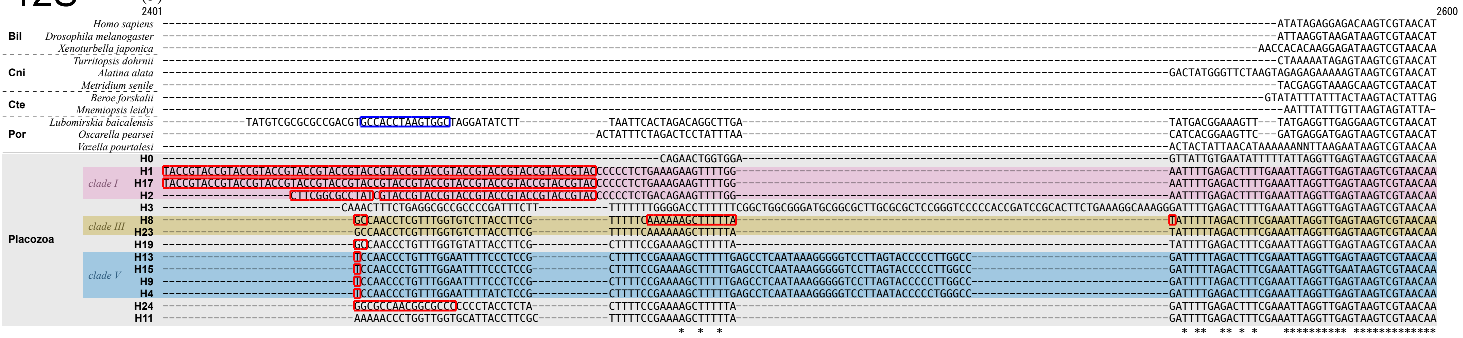
2401
2600
Bil
Homo sapiens
Drosophila melanogaster
Xenoturbella japonica
Cni
Turritopsis dohrnii
Alatina alata
Metridium senile
Cte
Beroe forskalii
Mnemiopsis leidyi
Por
Lubomirskia baicalensis
Oscarella pearsei
Vazella pourtalesi
Placozoa
H0
H1
H17
H2
H3
H8
H23
H19
H13
H15
H9
H4
H24
H11
clade I
clade III
clade V

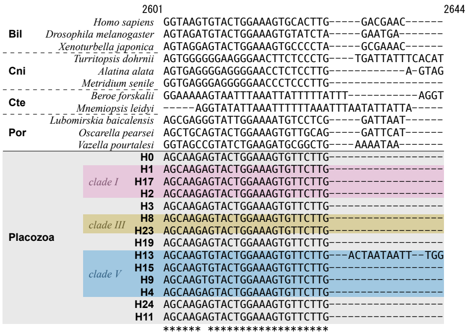
2601
2644
Bil
Homo sapiens
Drosophila melanogaster
Xenoturbella japonica
Cni
Turritopsis dohrnii
Alatina alata
Metridium senile
Cte
Beroe forskalii
Mnemiopsis leidyi
Por
Lubomirskia baicalensis
Oscarella pearsei
Vazella pourtalesi
Placozoa
H0
H1
H17
H2
H3
H8
H23
H19
H13
H15
H9
H4
H24
H11
clade I
clade III
clade V

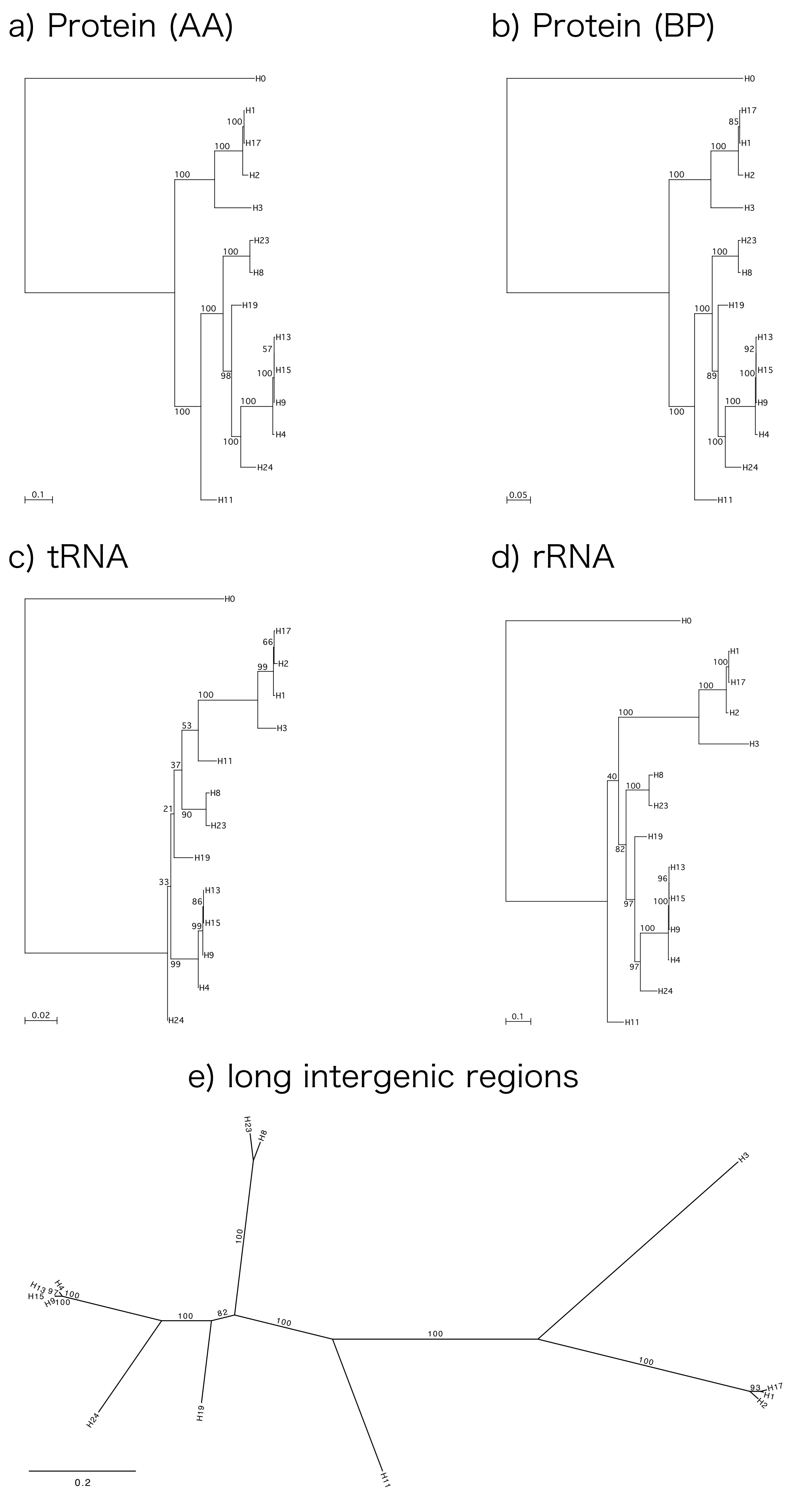
a) Protein (AA)
H0
H1
H17
H2
H3
H23
H8
H19
H13
H15
H9
H4
H24
H11
0.1
b) Protein (BP)
0.05
c) tRNA
0.02
d) rRNA
0.1
e) long intergenic regions
0.2

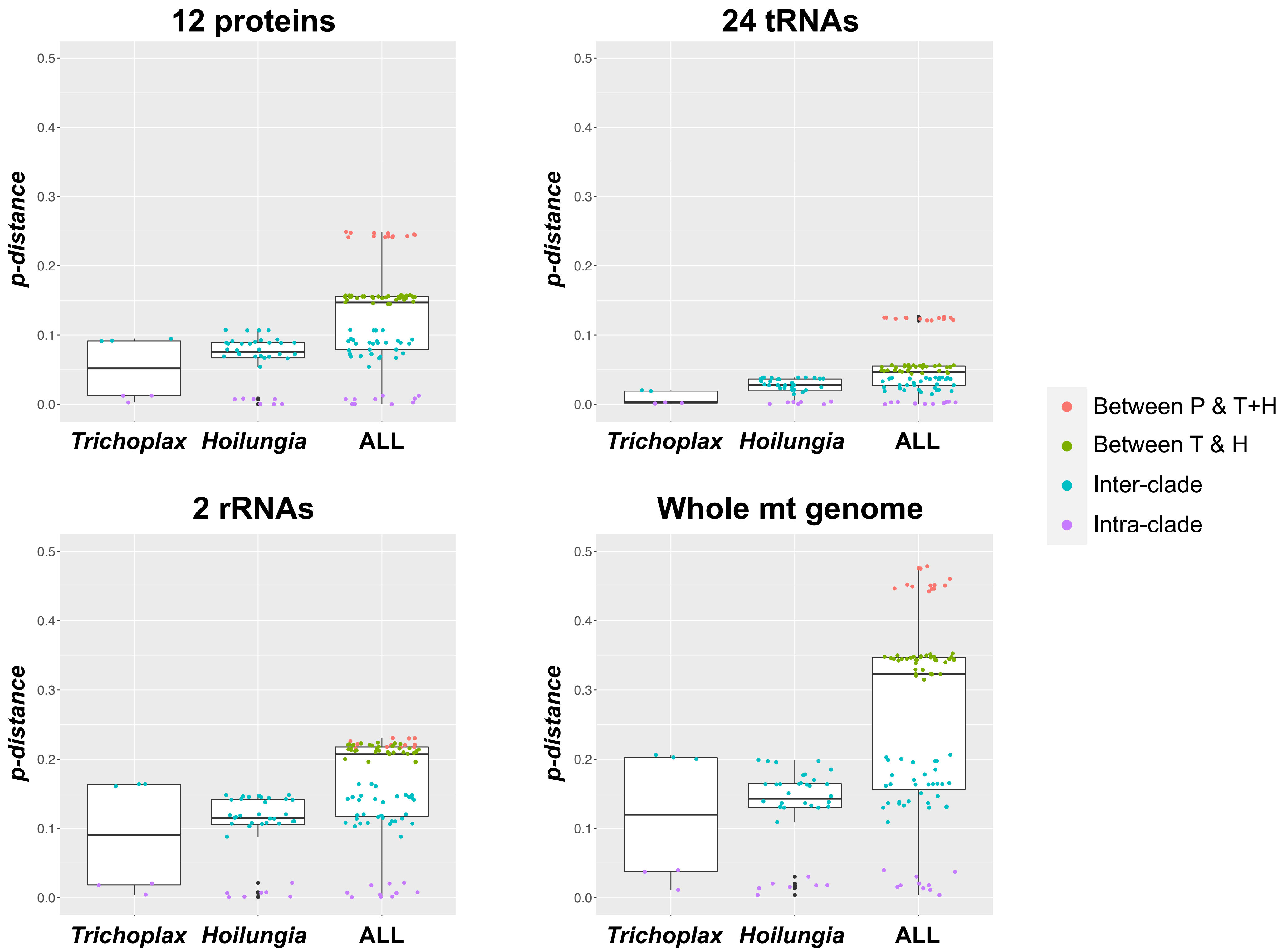
12 proteins
24 tRNAs
2 rRNAs
Whole mt genome
p-distance
0.0
0.1
0.2
0.3
0.4
0.5
Trichoplax
Hoilungia
ALL
Between P & T+H
Between T & H
Inter-clade
Intra-clade

a) Secondary structure of tRNA Ser of H0

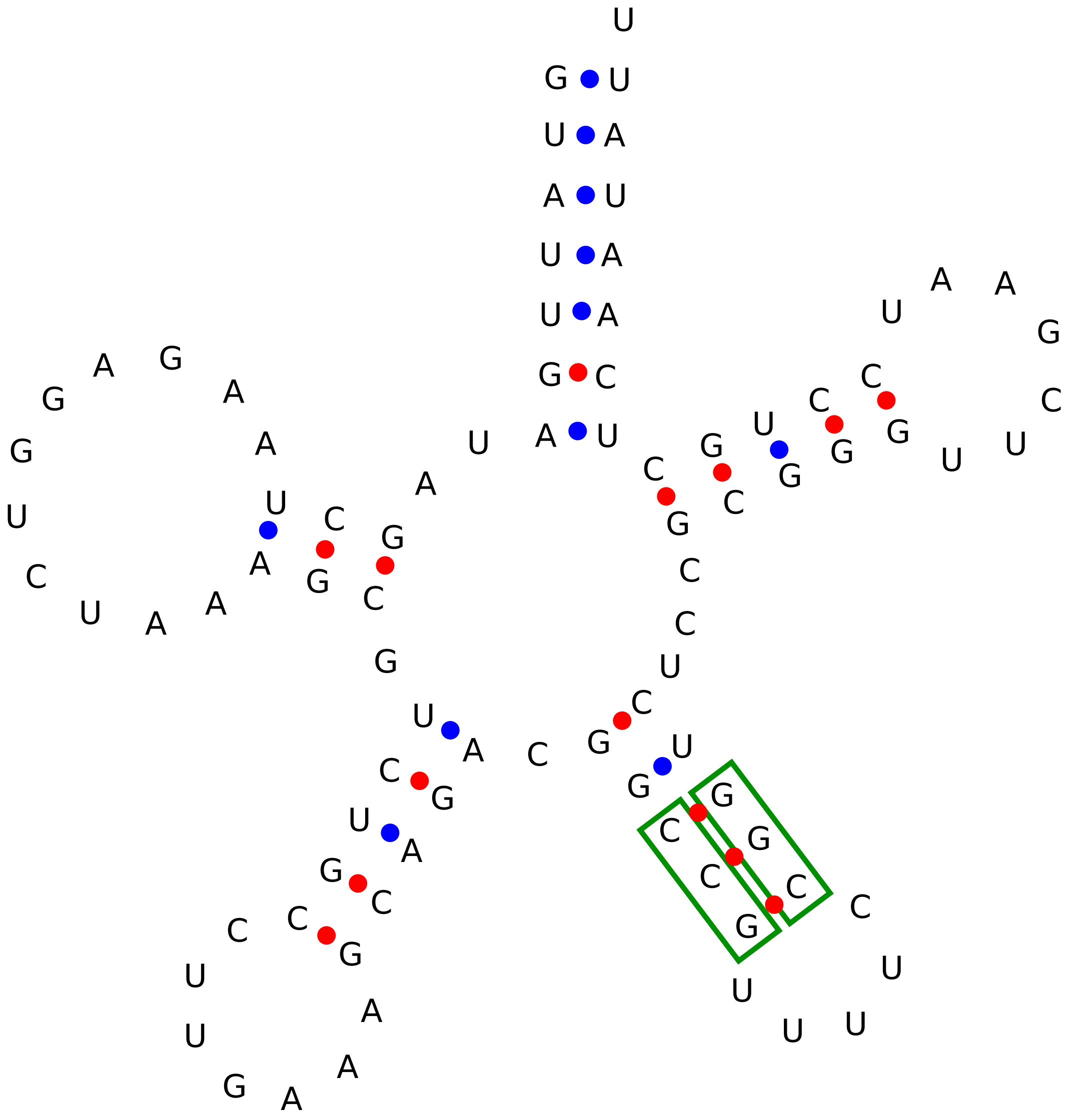

b) Alignments of tRNA Ser

	1 … 93
H0	GTATTGATAGCTAAG--AGGTCTAAAGCGTCTGCCTTGAAAGCAGACGGCCGTT---TTCCGGTCTCCGCGGGTTCGAATCCTGCTCAATATT
H1	GTATTGATAGCTAAG--CGGTCTAAAGCGTCTGCCTTGAAAGCAGGCGGGGCG-----TCGCCCCTACGCGGGTTCGAATCCTGCTCAATATT
H17	GTATTGATAGCTAAG--CGGTCTAAAGCGTCTGCCTTGAAAGCAGGCGGGGCG-----TCGCCCCTACGCGGGTTCGAATCCTGCTCAATACT
H2	GTATTGATAGCTAAG--CGGTCTAAAGCGTCTGCCTTGAAAGCAGGCGGGGCG-----TCGCCCCTACGCGGGTTCGAATCCTGCTCAATACT
H3	GTATTGATAGCTAAG--CGGTCTACAGCGTCTGCCTTGAAAGCAGGCGGGGCGGGAATCCGCCCCTACGCGGGTTCGAATCCTGCTCAATACT
H8	GTATTGATAGCTAAGTCCGGTTCAAAGCGTCTGCCTTGAAAGCAGACCGGCGCGG--AAAGCGCCCACGCGGGTTCGAATCCTGCTCAATACT
H23	GTATTGATAGCTAAGTCCGGTTCAAAGCGTCTGCCTTGAAAGCAGACCGGCGCGG--AAAGCGCCCACGCGGGTTCGAATCCTGCTCAATACT
H19	GTATTGATAGCTAAGTCTGGTTTAAAGCGTCTGCCTTGAAAGCAGGCGGGGCTG----ATGCCCCAACGCGGGTTCGAATCCTGCTCAATACT
H13	GTATTGATAGCTAAGTCCGGTTTAAAGCGTCTGCCTTGAAAGCAGGCCGGCGCCG--TTGGCGCCCACGCGGGTTCGAATCCTGCTCAATACT
H15	GTATTGATAGCTAAGTCCGGTTTAAAGCGTCTGCCTTGAAAGCAGGCCGGCGCCG--TTGGCGCCCACGCGGGTTCGAATCCTGCTCAATACT
H9	GTATTGATAGCTAAGTCCGGTTTAAAGCGTCTGCCTTGAAAGCAGGCCGGCGCCG--TTGGCGCCCACGCGGGTTCGAATCCTGCTCAATACT
H4	GTATTGATAGCTAAGTCCGGTTTAAAGCGTCTGCCTTGAAAGCAGGCCGGCGCCG--TTGGCGCCCACGCGGGTTCGAATCCTGCTCAATACT
H24	GTATTGATAGCTAAGTCCGGTTTAAAGCGTCTGCCTTGAAAGCAGGCCGGCGCCG--TTGGCGCCCACGCGGGTTCGAATCCTGCTCAATACT

Supplementary Table 1. Primer list

a) Universl Primers

Gene, Region	Primer name	sequence (5'-3')	applied haplotypes	Citation
ORF	LagR1	AAGGAGTCATTGAATAAAAAAATAGC	H9,	Miyazawa *et al.*, 2012
16S	S2006F	CGAGAAGACCCCATTGAGCTTTACTA	H2, H9, H11, H7, H19	Signorovitch *et al.*, 2006
16S	S2006R	TACGCTGTTATCCCCATGGTAACTTT	H2, H9, H11, H7, H19	Signorovitch *et al.*, 2006
nad5	nad5F1	TAGAAATAACAAGTAACATAGTAACAC	H9, H11, H17, H19	This study
atp6	atp6R1	TTTTAATTGGTTCTTTTGTGCCCTT	H9, H11	Miyazawa *et al.*, 2012
cob	cobF1	CTAATAACCCTGTCTTTCTTAATCTCAT	H2, H9, H11, H17, H19	Miyazawa *et al.*, 2012
cob	cobR1	CCTTTTGTTTTAGTTGGTCTTGTATT	H2, H17,	This study
*cox1*e1	cox1e1F1	GCTTTTAGTATGTTGATTAGATTGGATT	H2, H17,	Miyazawa *et al.*, 2012
*cox1*e6	cox1e6R1	ACAAATCTTTGGTTATTTAGGCATG	H11, H19	Miyazawa *et al.*, 2012
*cox1*e9	cox1e9F1	TTTTATGTTGCCAATGACTATCTGAAT	H9, H11, H17	This study
*cox1*e10	cox1e10R1	TAAGAAAATAAAACCTAAAGCCCAC	H9, H11, H17	This study
*cox1*e11	cox1e11R1	AGTTGGGGGTCCTAAATCCTCATA	H2, H9,	Miyazawa *et al.*, 2012
*cox1*e12	cox1e2R1	GTCATACCTGGTGTTCTCATATTC	H2, H17,	Miyazawa *et al.*, 2012
cox3	cox3R1	TTTATGTGTTTGTATATTGATGGGGAT	H9, H11, H17, H19	Miyazawa *et al.*, 2012
nad1	nad1R1	ACATAATAGCTGCTGGAAATAAAG	H2, H9, H11, H17, H19	Miyazawa *et al.*, 2012
nad3	nad3F1	CAATTCAAAAACTAAAAAGATTAAG	H19,	This study
nad4	nad4F1	TTATTTATTCTTGTCACTATTTTATATG	H2, H17,	Miyazawa *et al.*, 2012
nad6	nad6F1	CAAATAAAAAAATAATACAAATAGCGCC	H9,	Miyazawa *et al.*, 2012
nad6 - tRNA-N	nad6F2	GAAACGAGATTGAAAAGAATTGAACT	H2,	This study

b) Specific Primers for PacBio RSII

Haplotype	Primer name	sequence (5'-3')
H2	H2_S2006F	TGTGTATCAGTACATGCGAGAAGACCCCATTGAGCTTTACTA
H2	H2_S2006R	GAGTGCTACTCTAGTATACGCTGTTATCCCCATGGTAACTTT
H2	H2_cox14LR	TACTAGAGTAGCACTCGCTCAAACAATAAATCCTAATATACCTATG
H2	H2_cox3LF	GCAGAGTCATGTATAGCTAAATGATAAGGATGATAAACTAAAGAC
H2	H2_gCOX11F	ACACGCATGACACACTGATTTGGAAATTGATTTGTACCTTTAAT
H2	H2_gCOX11R	CATGTACTGATACACAAAATTTTCACCAAGTCATCACTAACTC
H2	H2_gCOX16R	ATGACGCATCGTCTGACCCAACCTAAATGTATTGATAGCTAAGT
H2	H2_gN6C16F	CTATACATGACTCTGCTTAAACTACAATCTCTCAAAGGAAGACC
H2	H2_gNAD1F	TCAGACGATGCGTCATCCCCCGATATAATAAATGACAATAAACA
H2	H2_gNAD1R	GCATATAGTAGAGATCCACTTAATAAAACTCCTTGGTCCATTGT
H2	H2_gNAD4F	GATCTCTACTATATGCTAGTGGAAACTTTATTGGGGAATATTTA
H2	H2_gNAD4R	AGTGTGTCATGCGTGTTATTAGAAATGCGAAAAAGATCATAGAA
H9	H9_ATP6LR	ATCTGTGCGAGACTACTTTTAATTGGTTCTTTTGTGCCCTT
H9	H9_C145bF2	GTAGTCTCGCACAGATTAGCATAAAAAAAAAACTTAGAGAG
H9	H9_LagLR2	ACGCGCACAGAGTCTCGCACCTTCCATCAGCCCTACGACTCAT
H9	H9_NAD51LF	CGAGCACGCGCGTGTGTAGAAATAACAAGTAACATAGTAACAC
H9	H9_NAD6LF	GAGACTCTGTGCGCGTCAAATAAAAAAATAATACAAATAGCGCC
H9	H9_V2004R	CACGCACACACGCGCGTTGATCGTTGTCTATCCCAC
H9	H9_cobLF	GAGATCGACAGTCTCGCTAATAACCCTGTCTTTCTTAATCTCAT
H9	H9_cox12LF	GCTCGACTGTGAGAGAGGAGGGGGTGATCCTATTTTATATCAAC
H9	H9_cox15bLR	CACACGCGCGTGCTCGTAAGAAAATAAAACCTAAAGCCCAC
H9	H9_n4lLR	TCTCTCACAGTCGAGCTGACAACAGGGTATTTATTAGTATCT
H9	H9_rnLbLR2	CGAGACTGTCGATCTCTAACTCTGACCGTGATAATGTAGCG
H9	H9_tPheLR	CGCGCGTGTGTGCGTGCTTCAGTTTAGTGCTCTACCATTTGA
H11	H11_S2006F	CAGCAGTATAGACTGTCGAGAAGACCCCATTGAGCTTTACTA
H11	H11_S200R	ATGATGTGCTACATCTTACGCTGTTATCCCCATGGTAACTTT
H11	H11_cobLF	ACAGTCTATACTGCTGCTAATAACCCTGTCTTTCTTAATCTCAT
H11	H11_cox15bLR	GTCGTAGAGCACGCAGTAAGAAAATAAAACCTAAAGCCCAC
H11	H11_gA6RLAR	AGTCATCGTATCGCGCTTTTCTGCGAAAACTATCTAAGTAGTGC
H11	H11_gCOX11F	GCGCACGCACTACAGAATCTTACCCTACCTTAGTTATGCCTACA
H11	H11_gCOX11R	CGATCAGCTGAGCGCGAGAGCCTAGTAATAGAAATAGAGCTGGA
H11	H11_gCOX15aF2	GCGCGATACGATGACTTTTACAGCAGCAACAATGATTAT
H11	H11_gN5C3R	AGATGTAGCACATCATATTTTAGTAAAGTGAAAGGAGAGAATCG
H11	H11_gNAD51F	CTGCGTGCTCTACGACATATAATTCTATATGAACAACGGCATCA
H11	H11_n4lLR	TCTGTAGTGCGTGCGCTGACAACAGGGTATTTATTAGTATCT
H17	H17_S2006F	TAGATGCGAGAGTAGACGAGAAGACCCCATTGAGCTTTACTA
H17	H17_S2006R	AGCTCTGAGTCTCTATTACGCTGTTATCCCCATGGTAACTTT
H17	H17_cox14LR	ATAGAGACTCAGAGCTGCTCAAACAATAAATCCTAATATACCTATG
H17	H17_cox3LF	TATACGTATATAGACGCTAAATGATAAGGATGATAAACTAAAGAC
H17	H17_gCOX11F	CATAGCGACTATCGTGGATTTGGAAATTGATTTGTACCTTTAAT
H17	H17_gCOX11R	TCTACTCTCGCATCTAAAATTTTCACCAAGTCATCACTAACTC
H17	H17_gCOX16R	GTCGCGACGTCAGTGTCCCAACCTAAATGTATTGATAGCTAAGT
H17	H17_gN6C16F	CGTCTATATACGTATATTAAACTACAATCTCTCAAAGGAAGACC
H17	H17_gNAD1F	ACACTGACGTCGCGACCCCCCGATATAATAAATGACAATAAACA
H17	H17_gNAD1R	ATCTAGCGTAGTGATGCACTTAATAAAACTCCTTGGTCCATTGT
H17	H17_gNAD4F	CATCACTACGCTAGATTAGTGGAAACTTTATTGGGGAATATTTA
H17	H17_gNAD4R	CACGATAGTCGCTATGTATTAGAAATGCGAAAAAGATCATAGAA
H19	H19_COX16LFF	CGTGTCGCGCATATCTAACAACACCACCAGAACATCATACTTA
H19	H19_N3C13LF	ATATCAGTCATGCATATGTACAGGACTCGAACCTATAATAAT
H19	H19_NAD6R2	TAGTCACAGCGTGTACGAAACGAGATTGAAAAGAATTGAACT
H19	H19_NAF51LF	GTACACGCTGTGACTATAGAAATAACAAGTAACATAGTAACAC
H19	H19_S2006F	TGCATGCACAGATGCGCGAGAAGACCCCATTGAGCTTTACTA
H19	H19_S2006R	TATGTGATCGTCTCTCTACGCTGTTATCCCCATGGTAACTTT
H19	H19_cobLF	CGCATCTGTGCATGCACTAATAACCCTGTCTTTCTTAATCTCAT
H19	H19_cox2R	AGATATGCGCGACACGGAACATTGACCATAAAAAATGCCA
H19	H19_n4lLR	TATGCATGACTGATATTGACAACAGGGTATTTATTAGTATCT
H19	H19_tPheLR	GAGAGACGATCACATACTTCAGTTTAGTGCTCTACCATTTGA

Clade	Haplotype	ORF	region	Result of BLAST search<br>Accession Number	Organism	name	evalue
I	H17	orf141	complement(10360..10785)	YP_654091.1 (ORF168 in Fig. 1)	*Trichoplax adhaerens* (H1)	hypothetical protein	3e-48
		RVT-IM	27214..27660	ABI53799.1	*Placozoan* sp. H3	hypothetical protein	6e-50
		RVT-IM	27750..29102	ABI53799.1	*Placozoan* sp. H3	hypothetical protein	1e-92
		LAG_1	29850..30662	YP_654097.1	*Trichoplax adhaerens* (H1)	hypothetical protein	4e-151
		LAG_1	30722..31402	YP_654097.1	*Trichoplax adhaerens* (H1)	hypothetical protein	3e-148
	H2	RVT-IM	28493..29674	ABI53799.1	*Placozoan* sp. H3	hypothetical protein	2e-73
		LAG_1	31510..32562	YP_654097.1	*Trichoplax adhaerens* (H1)	hypothetical protein	0.0
III	H23	LAG_2	complement(6097..6816)	QCQ69104.1	*Powellomyces hirtus*	LAGLIDADG endonuclease	3e-55
		LAG_1	complement(26822..28327)	ABI53769.1	*Placozoan* sp. H8	hypothetical protein	0.0
IV	H19	RVT-IM	complement(6116..7615)	ABI53784.1	*Placozoan* sp. H4	hypothetical protein	0.0
		RVT-IM	complement(7735..8157)	ABI53784.1	*Placozoan* sp. H4	hypothetical protein	1e-60
		LAG_1	complement(25960..27507)	ABI53769.1	*Placozoan* sp. H8	hypothetical protein	0.0
V	H9	RVT-IM	complement(10171..11370)	ABI53784.1	*Placozoan* sp. H4	hypothetical protein	9e-67
		RVT-IM	complement(10171..11370)	ABI53784.1	*Placozoan* sp. H4	hypothetical protein	1e-99
		LAG_1	complement(30717..31790)	YP_004327738.1	*Placozoan* sp. H15	hypothetical protein	0.0
		LAG_1	complement(31745..32290)	YP_004327738.1	*Placozoan* sp. H15	hypothetical protein	8e-120
		polB	complement(5478..7655)	YP_004327733.1	*Placozoan* sp. H15	DNA polymerase type B	0.0
VII	H24	LAG_2	complement(5782..6501)	YP_009463045.1	*Pleurotus platypus*	homing endonuclease	6e-57
		RVT-IM	complement(7270..9615)	ABI53784.1	*Placozoan* sp. H4	hypothetical protein	0.0
		orf143	complement(18247..18678)	YP_009350057.1	*Spongospora subterranea*	hypothetical protein	2e-12
		LAG_1	complement(27862..29529)	ABI53769.1	*Placozoan* sp. H8	hypothetical protein	1e-158
VI	H11	RVT-IM	complement(6271..8313)	ABI53784.1	*Placozoan* sp. H4	hypothetical protein	0.0
		LAG_1	complement(27091..28536)	ABI53769.1	*Placozoan* sp. H8	hypothetical protein	5e-153
		orf425	complement(29696..30973)	YP_009485446.1	*Halamphora coffeaeformis*	cox1I1a maturase	2e-09

S2007 = Signorovitch et al., 2007; M2012 = Miyazawa et al., 2012; RVT-IM = ORF containing a reverse transcriptase domain and a group II intron maturase domain; LAG = ORF containing a group I intron LAGLIDADG endonuclease domain; pol B = fungal DNA-directed DNA polymerase type B
